# Macrophage HM13/SPP Enhances Foamy Macrophage Formation and Atherogenesis

**DOI:** 10.1002/advs.202412498

**Published:** 2025-03-20

**Authors:** Yu Cao, Qirong Xie, Qiang Zheng, Jingping Zhang, Mengyu Yao, Zhongyong Du, Lujun Zhang, Tianyang Hu, Yunli Zhao, Jianlin Du, Yongyong Li, Yuxing Feng, ND Melgiri, Xiaodong Zhao, Rongzhong Huang, Yang Sun

**Affiliations:** ^1^ Department of Cardiovascular Surgery the First People’s Hospital of Yunnan Province No. 157, Jinbi Road, Xishan District Kunming Yunnan 650032 China; ^2^ Center for Translational Research in Clinical Medicine the Affiliated Hospital of Kunming University of Science and Technology No. 68, Wenchang Road, Wuhua District Kunming Yunnan 650093 China; ^3^ Department of Ultrasound Chongqing Key Laboratory of Ultrasound Molecular Imaging the Second Affiliated Hospital of Chongqing Medical University No. 76, Linjiang Road, Yuzhong District Chongqing 400010 China; ^4^ Department of Hematopathology the First People's Hospital of Yunnan Province No. 157, Jinbi Road, Xishan District Kunming Yunnan 650032 China; ^5^ Cell Therapy Engineering Research Center for Cardiovascular Diseases in Yunnan Province Yunnan Key Laboratory of Innovative Application of Traditional Chinese Medicine the First People's Hospital of Yunnan Province No. 157, Jinbi Road, Xishan District Kunming Yunnan 650032 China; ^6^ Precision Medicine Center the Second Affiliated Hospital of Chongqing Medical University No. 76, Linjiang Road, Yuzhong District Chongqing 400010 China; ^7^ Department of Cardiology the Second Affiliated Hospital of Chongqing Medical University No. 76, Linjiang Road, Yuzhong District Chongqing 400010 China; ^8^ Department of Geriatric Medicine the Second Affiliated Hospital of Chongqing Medical University No. 76, Linjiang Road, Yuzhong District Chongqing 400010 China; ^9^ Department of Rehabilitation and Pain Medicine the Ninth People's Hospital of Chongqing No. 69, Jialing Village, Beibei District Chongqing 400700 China; ^10^ Impactys Foundation for Biomedical Research 10300 Campus Pointe Drive San Diego CA 92121 USA

**Keywords:** AIP, atherosclerosis, foamy macrophage, HM13, HO‐1, macrophage, SPP

## Abstract

Aryl Hydrocarbon Receptor‐Interacting Protein (AIP) reduces macrophage cholesterol‐ester accumulation and may prevent atherogenic foamy macrophage formation. Analyzing *AIP*‐associated regulatory gene networks can aid in identifying key regulatory mechanism(s) underlying foamy macrophage formation. A weighted gene co‐expression network analysis on the Stockholm Atherosclerosis Gene Expression (STAGE) patient cohort identifies *AIP* as a negative correlate of Histocompatibility Minor 13 (*HM13*), which encodes the ER‐associated degradation (ERAD) protein Signal Peptide Peptidase (HM13/SPP). The negative correlation between AIP and HM13/SPP on mRNA and protein levels is validated in oxLDL‐stimulated macrophages and human plaque foamy macrophages. Mechanistically, AIP, via its chaperone interaction with Aryl Hydrocarbon Receptor (AHR), inhibits p38‐c‐JUN‐mediated *HM13* transactivation, thereby suppressing macrophage lipid accumulation. Myeloid HM13/SPP overexpression enhances oxLDL‐induced foamy macrophage formation in vitro as well as atherogenesis and plaque foamy macrophage load in vivo, while myeloid HM13/SPP knockout produces the opposite effects. Mechanistically, myeloid HM13/SPP enhances oxLDL‐induced foamy macrophage formation in vitro as well as atherogenesis and plaque foamy macrophage load in vivo via promoting ERAD‐mediated proteasomal degradation of the metabolic regulator Heme Oxygenase‐1 (HO‐1). In conclusion, AIP downregulates macrophage HM13/SPP, a driver of oxLDL‐induced lipid loading, foamy macrophage generation, and atherogenesis.

## Graphical abstract

Previous research suggests that AIP may prevent atherogenic foamy macrophage formation. This study reveals that AIP, via its chaperone interaction with AHR, inhibits p38‐c‐JUN‐mediated transactivation of *HM13*, which encodes the ERAD protease HM13/SPP. HM13/SPP promotes foamy macrophage formation in addition to atherogenesis and plaque foamy macrophage load via promoting ERAD‐mediated proteasomal degradation of the metabolic regulator HO‐1.

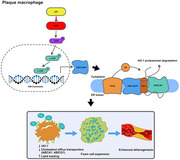

## 1 Introduction

Atherosclerosis‐related disorders–such as coronary artery disease (CAD), myocardial infarction, cerebrovascular disease (CVD), stroke, and peripheral artery disease (CAD) –lead to ≈50% of all global deaths.^[^ ^1^ ^]^ Atherosclerosis is induced when dysfunctional lipid‐laden macrophages (termed “foamy macrophages”) are formed from plaque macrophages in the arterial wall.^[^ ^2^ ^]^ This transformation is caused by excessive oxidized low‐density lipoprotein (ox‐LDL) intake and cholesterol esterification coupled with insufficient cholesterol efflux.^[^ ^2^ ^]^ Foamy macrophages have a greater propensity for apoptosis and are unable to clear apoptotic cells efficiently.^[^ ^2^ ^]^ This increases the rate of secondary necrosis and leads to the growth of atherosclerotic plaques. Thus, it is imperative to develop new therapeutic strategies to battle atherogenic foamy macrophage formation.

Unfortunately, few apical regulator(s) that govern atherogenic foamy macrophage formation have been identified and characterized. Systems genetics research has identified four key myeloid RNA‐processing regulatory gene network (RGN) driver genes, namely *AIP* (*ARA9*, *XAP2*), *DRAP1*, *POLR21*, and *PQBP1*, that may regulate atherosclerotic foamy macrophage generation.^[^ ^3^ ^]^ In acLDL‐exposed THP‐1 macrophages, knockdown of DRAP1, POLR21, and PQBP1 reduces cholesterol‐ester accumulation, while knockdown of Aryl Hydrocarbon Receptor‐Interacting Protein (AIP) promotes cholesterol‐ester accumulation.^[^ ^3^ ^]^ This evidence indicates that AIP inhibits macrophage cholesterol‐ester accumulation in vitro and suggests that AIP may inhibit atherosclerotic foamy macrophage generation. Analyzing the gene networks associated with *AIP* and other key atherosclerosis‐associated RGN driver genes can aid us in identifying key regulatory mechanism(s) underlying foamy macrophage formation in atherosclerotic plaques.

Therefore, here we employed a weighted gene co‐expression network analysis (WGCNA) on the transcriptomic database of the Stockholm Atherosclerosis Gene Expression (STAGE) patient cohort^[^ ^4^ ^]^ to identify biologically relevant gene clusters (modules) that significantly correlate with *AIP* and the other key atherosclerosis‐associated RGN driver genes (*DRAP1*, *POLR21*, and *PQBP1*). We discovered *AIP* to be a negative correlate of Histocompatibility Minor 13 (*HM13*), which encodes the endoplasmic reticulum‐associated degradation (ERAD) protease Signal Peptide Peptidase (HM13/SPP).^[^ ^5^ ^]^ We show that AIP, through its interaction with AHR, downregulates macrophage HM13/SPP, a driver of oxLDL‐induced lipid accumulation, foamy macrophage generation, and atherogenesis. Mechanistically, HM13/SPP enhances foamy macrophage formation and atherosclerosis via promoting ERAD‐mediated proteasomal degradation of the metabolic regulator Heme Oxygenase‐1 (HO‐1).

## 2 Results

### 2.1 WGCNA Identifies Three AIP‐Associated Gene Modules in Human Atherosclerotic Plaques

**Figure 1:**
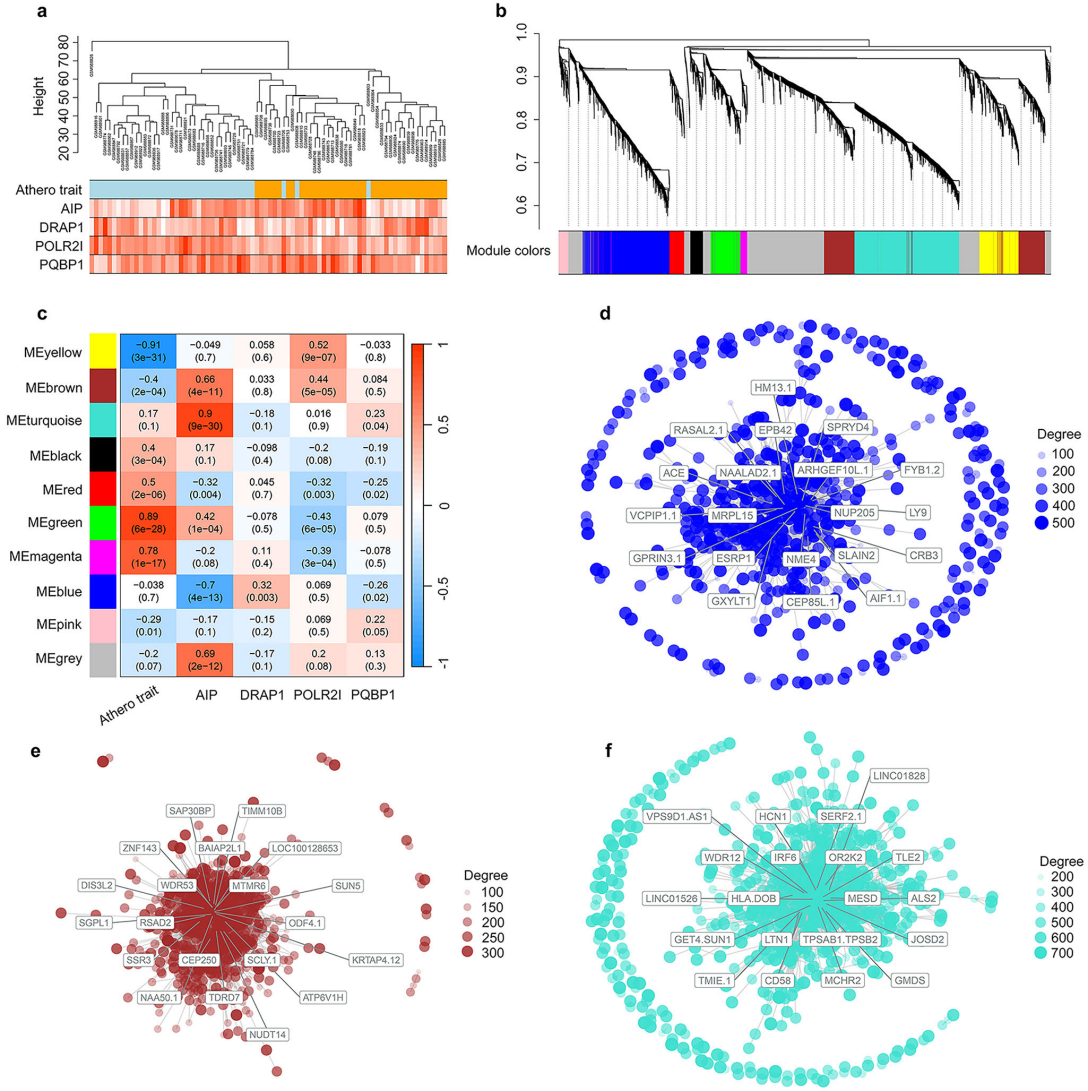
Weighted Gene Co‐Expression Network Analysis (WGCNA) Identifies Three *AIP*‐Associated Gene Modules in Human Atherosclerotic Plaques. a) Sample dendrogram and trait heatmap illustrating the atherosclerosis trait (“Athero trait”) variable (orange = atherosclerotic aortic root, light blue = non‐atherosclerotic IMA) and the associated normalized expression of the four atherosclerosis RGN driver genes (i.e., *AIP*, *DRAP1*, *PLR2I*, and *PQBP1*). Higher gene expression is depicted in red. b) Cluster dendrogram showing the module assignment for the ten modules. c) Heatmap indicating the positive correlations (red) and negative correlations (blue) for the atherosclerosis trait (“Athero trait”) and the four atherosclerosis RGN driver genes. d‐f) Visual representations of modular networks for the d) Blue, e) Brown, and f) Turquoise modules. The labeled genes within each module are the top‐ranking genes in terms of connectivity.

A WGCNA approach was implemented to identify biologically meaningful gene modules highly correlated with the four key atherosclerosis‐associated RGN driver genes (i.e., *AIP*, *DRAP1*, *POLR2I*, and *PQBP1*). Transcriptomic microarray data from atherosclerotic aortic root wall samples and matching non‐atherosclerotic IMA wall samples from 40 STAGE cohort CAD patients were selected for this WGCNA (Gene Expression Omnibus (GEO) acc. no: GSE40231).^[^ ^4^ ^]^ A sample dendrogram illustrates the phenotypic trait variable (atherosclerotic aortic root or non‐atherosclerotic IMA) and the associated expression of the RGN driver genes (**Figure** **1**a). WGCNA modules were defined using biweight midcorrelation (bicor), with the top 20% most variable annotated genes (*n* = 8933) and a soft‐threshold power of eight to achieve approximate scale‐free topology (*R* ^2^ > 0.8) (Figure S1, Supporting Information). The simulation of different combinations of hyperparameters (i.e., deep split, minimal module size, merge threshold, and pamstage^[^ ^6^ ^]^) revealed that the modules were relatively robust to these hyperparameters despite the moderate size of the dataset (Figure S2, Supporting Information). The final network resulted in ten modules, ranging in membership from 155 to 1870 genes (Figure 1b; Figure S3a,b, Supporting Information). Application of a differential co‐expression analysis to characterize the WGCNA modules revealed that most modules (8/10) were preserved across the two conditions (atherosclerotic aortic root wall samples vs matching non‐atherosclerotic IMA samples, *p* > 0.05), although differences were found at the gene level (Table S6, Supporting Information). We evaluated the preservation of the modules relative to tissue‐specific networks from the STAGE dataset (i.e., liver, skeletal muscle, and visceral fat) using a set of seven parameters described in the Methods.^[^ ^7^ ^]^ A total of 6/10 modules (i.e., Black, Blue, Brown, Pink, Red, and Turquoise) were significantly preserved (*p* < 0.05) across all seven parameters (Table S7, Supporting Information).

*AIP* and *PQBP1* were found in the Turquoise module, while *POLR2I* was found in the Brown module. *DRAP1* did not belong to any module, as it was not in the top 20% of most variable genes. Each of the module's ME values was Pearson‐correlated with sample traits defined by *AIP*, *DRAP1*, *POLR2I*, or *PQBP1* expression in the atherosclerotic samples. None of the four genes displayed a strong relationship with the atherosclerotic trait; however, *AIP* displayed the strongest correlations with the modules among the four genes (Figure 1c). Therefore, we selected the modules with the strongest *AIP* correlations (i.e., Turquoise [*R* = 0.90], Blue [*R* = −0.70], and Brown [*R* = 0.66]) for further analysis (note: the Grey module, which groups non‐significant genes, was excluded) (Figure 1d–f). Using all members of each module, functional enrichment for the Blue module (Figure S4a–d, Supporting Information), Brown module (Figure S5a–d, Supporting Information), and Turquoise module (Figure S6a–d, Supporting Information) was assessed by interrogating the GO‐BP, BioCarta, KEGG, and Reactome databases. Consistent with *AIP*’s systems genetics‐derived RGN,^[^ ^3^ ^]^ the Blue and Turquoise modules were both significantly enriched for RNA processing.

### 2.2 In Silico Analysis on the *AIP*‐Associated Gene Modules Identifies the ERAD Protease Gene *HM13*

**Figure 2:**
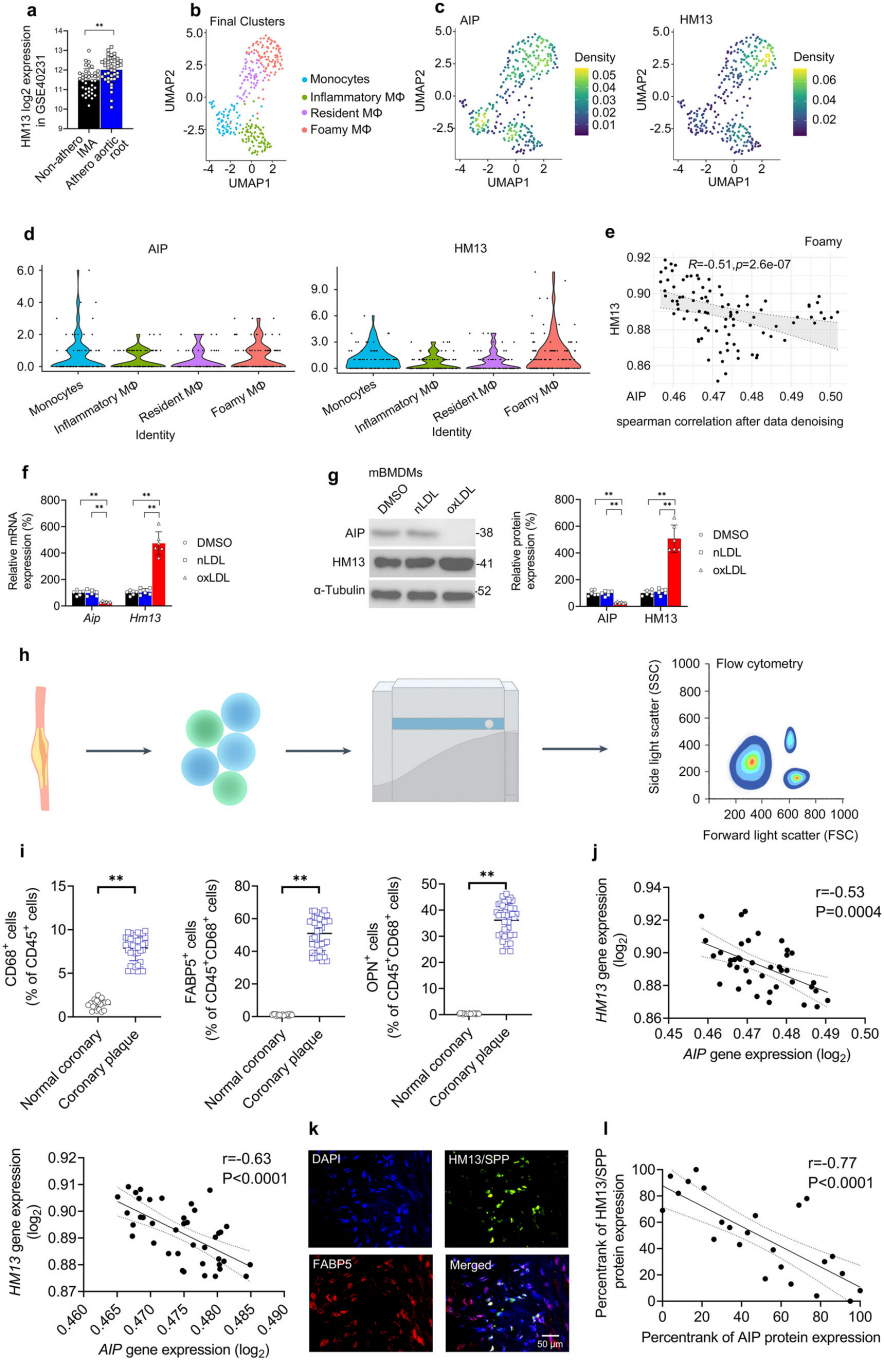
In Silico Analysis on the *AIP*‐Associated Gene Modules Identifies the ERAD Gene *HM13*. a) *HM13* (log_2_) expression in the STAGE atherosclerotic plaque cohort (GSE40231). b–e) scRNAseq analysis of human atherosclerotic carotid plaque scRNAseq data (GSE224273). b) Uniform manifold approximation and projection (UMAP) visualization depicting the four plaque myeloid clusters. c) UMAP visualizations and d) violin plots depicting *AIP* and *HM13* expression across the plaque myeloid clusters. e) Spearman correlation plot depicting a significant negative correlation between *AIP* and *HM13* in foamy macrophages. f,g) Murine bone marrow‐derived macrophages (mBMDMs) were analyzed after incubation with DMSO vehicle or oxLDL (25 µg mL^−1^) for 24 h by f) qPCR and g) Western blotting. H–k) Coronary artery specimens from 20 human donors (*n* = 40 plaque samples, 20 normal samples) were digested and subjected to FACS. h) Schematic overview. i) Analyses of FACS‐sorted cell subsets. j,k) Pearson correlation analyses of *AIP* and *HM13* gene expression in j) coronary plaque FABP5^+^CD45^+^CD68^+^ foamy macrophages and k) coronary plaque OPN^+^CD45^+^CD68^+^ foamy macrophages. l) Representative immunofluorescent images from human carotid atheromas (*n* = 2) displaying overlapping FABP5^+^ (green) and HM13/SPP^+^ (red) staining. Scale bar, 50 µm. m) Pearson correlation analyses of AIP and HM13/SPP protein expression in *ex vivo* carotid plaque foamy macrophages. a) *n* = 40 patients (STAGE cohort; GSE40231); b–e) *n* = 6 patients (GSE224273); f,g) *n* = 6 independent biological replicates per cohort; i–k) *n* = 20 independent coronary specimens, each divided into two plaque samples and one normal sample; and m) *n* = 8 independent carotid specimens, each divided into three *ex vivo* cultures. Data expressed as means ± SDs compared using a) paired Student's *t*‐test and f,g) one‐way ANOVA or medians ± upper/lower quartiles compared using i) Wilcoxon signed‐rank test. ^*^ *p* < 0.05, ^**^ *p* < 0.01.

Using an independent coronary atherosclerotic plaque gene expression dataset (GEO acc. no. GSE11138^[^ ^8^ ^]^), the Blue, Brown, and Turquoise modules were taken for final validation and correlation analyses with a curated 19‐gene Macrophage Plaque Gene Signature obtained from a previous study^[^ ^9^ ^]^ (Figure S7a, Supporting Information). Module Hub Gene Signature Scores were generated using the top 120‐ranking hub genes from each module and a ranking system robust to differences between samples and datasets. Among the three modules, the Blue Module Hub Gene Signature Score displayed the strongest positive correlation (*R* = 0.21, *P* = 0.091) with the Macrophage Plaque Gene Signature Score (Figure S7b, Supporting Information). Therefore, a final set of the highest‐ranking Blue module candidate genes (*n* = 47) was constructed (Figure S8a, Supporting Information). Consistent with the WGCNA findings (Figure 1c), 85% (40/47) of these highest‐ranking Blue module genes negatively correlated with *AIP* (*P* < 0.05). The three highest‐ranking Blue module genes among these 40 candidate genes were: *NME4*, *LY9*, and *HM13* (Figure S8b, Supporting Information). Of these three genes, only the ERAD protease gene *HM13* was significantly upregulated in the STAGE atherosclerotic plaque cohort (**Figure** **2**a), suggesting *HM13* may contribute to atherogenesis.

The foregoing evidence suggests a negative relationship between *AIP* and *HM13* expression in plaque macrophages. To understand how *AIP* and *HM13* co‐express in human atherosclerotic plaque macrophages, we analyzed an independent human atherosclerotic carotid plaque scRNAseq dataset (GSE224273)^[^ ^10^ ^]^ by cell type (Figure S9a, Supporting Information). The highest levels of both genes were observed in macrophages, classical monocytes, platelets, and memory B‐cell clusters (Figure S9b,c, Supporting Information). After subsetting and re‐clustering of the original plaque myeloid subpopulations (i.e., macrophages and classical monocytes) (Figure S10a,b, Supporting Information), four distinct plaque myeloid clusters were identified: monocytes, inflammatory macrophages, resident macrophages, and foamy macrophages (Figure 2b). Although *AIP* and *HM13* were clearly detected across all four plaque myeloid clusters (Figure 2c), foamy macrophages displayed the most prominent *HM13* expression (Figure 2d). Consistent with our WGCNA findings, *HM13* was negatively correlated with *AIP* in foamy macrophages (Figure 2e). Analysis across the M1 (inflammatory)‐M2 (anti‐inflammatory) macrophage polarization spectrum (i.e., M1, M4, Mox, Mhem, M(Hb), and M2)^[^ ^11^ ^]^ revealed *AIP* and *HM13* expression across the polarization spectrum but with minimal expression in M2 macrophages (Figure S11a,b, Supporting Information). For in vitro validation, we incubated non‐polarized mBMDMs with oxLDL for 24 h to induce lipid loading and mimic in vivo foamy macrophage formation; nLDL was employed as a control (Figure S12a–c, Supporting Information). oxLDL (but not nLDL) downregulated macrophage AIP mRNA and protein expression and upregulated HM13/SPP mRNA and protein expression (Figure 2f,g).

To extend these findings to human atherosclerosis, we FACS‐isolated CD45^+^CD68^+^ macrophages from human coronary atherosclerotic plaques and matched normal coronary artery samples (n = 20 donors, two plaque samples, and one normal sample per donor) (Figure 2h) (note: the myeloid lineage marker CD45 was used to exclude macrophage‐like VSMCs that express CD68^[^ ^12^ ^]^). As expected, there were significantly higher percentages of CD45^+^CD68^+^ macrophages and foamy macrophages (i.e., FABP5^+^CD45^+^CD68^+^ and OPN^+^CD45^+^CD68^+^) in coronary plaques relative to normal samples (Figure 2i; see Methods (Supporting Information) regarding the selection of the foamy macrophage‐specific markers FABP5 and OPN). Consistent with our scRNAseq‐based profiling, qPCR analyses in coronary plaque foamy macrophages revealed that *FABP5* and *OPN* are negatively correlated with *AIP* but positively correlated with *HM13* (Figure S13a–d, Supporting Information). qPCR analyses in coronary plaque foamy macrophages verified a significant negative correlation between *AIP* and *HM13* (Figure 2j,k). Human carotid plaques were used for protein‐level analyses, as they contain high levels of CD45^+^CD68^+^ macrophages.^[^ ^13^ ^]^ Immunofluorescent staining confirmed HM13/SPP's co‐localization with FABP5 (Figure 2l). Moreover, foamy macrophages isolated from human carotid plaques for 24 h ex vivo culture and ELISA (n = 8 donors, three ex vivo cultures per donor) displayed a negative correlation between AIP protein expression and HM13/SPP protein expression (Figure 2m).

### 2.3 AIP's Interaction with AHR Inhibits p38‐c‐JUN‐Mediated *HM13* Transactivation, Thereby Suppressing Macrophage Lipid Accumulation

**Figure 3:**
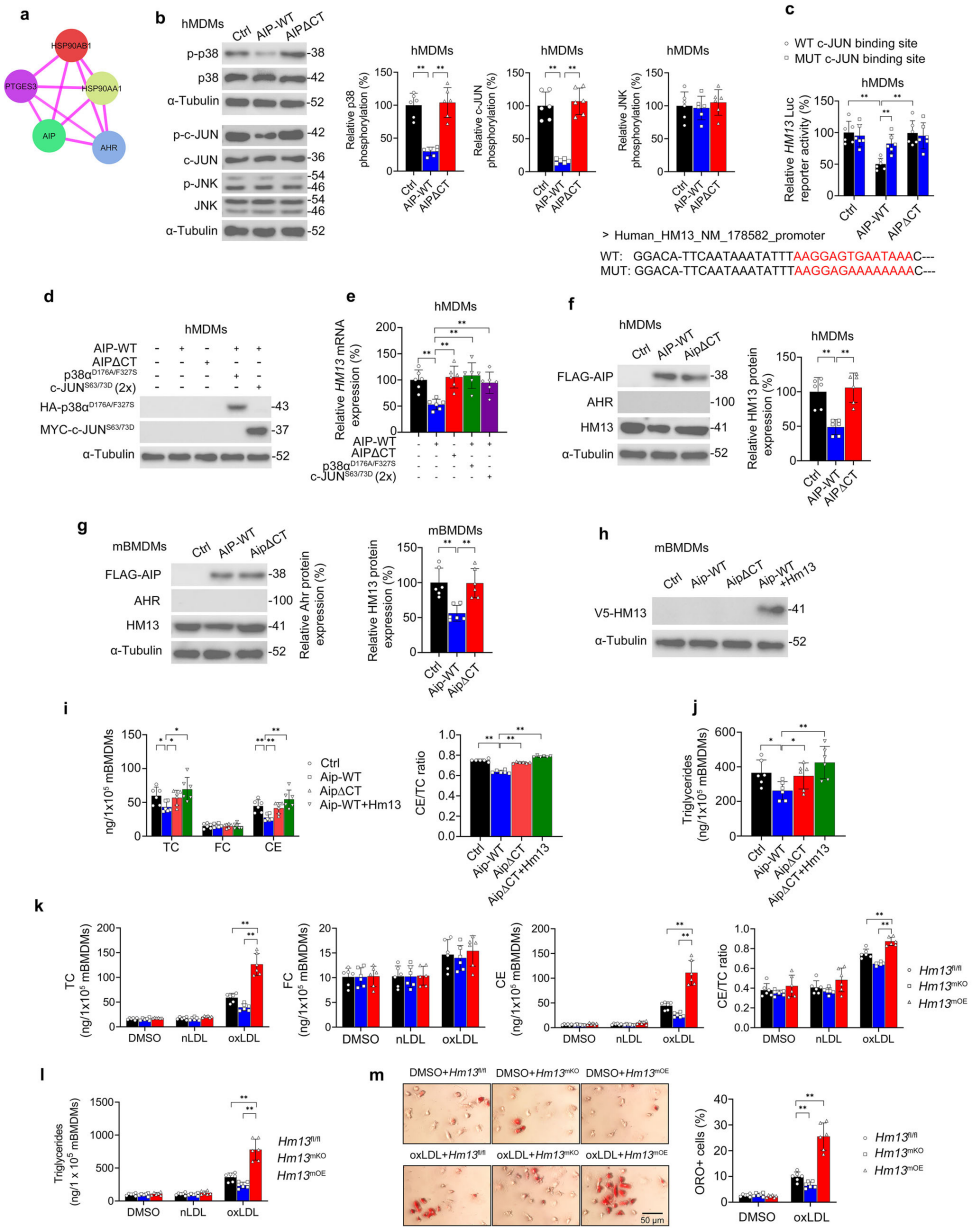
AIP's Interaction with AHR Inhibits p38‐c‐JUN‐Mediated *HM13* Transactivation, Suppressing Macrophage Lipid Accumulation. a) Cytoscape schematic of STRING protein‐protein interaction (PPI) analysis (high confidence = 0.700) showing direct PPIs between AIP, AHR, and other chaperone proteins. Pink edges indicate experimental evidence. b) Western blotting analyses of p38 phosphorylation, c‐JUN phosphorylation, and JNK phosphorylation in human monocyte‐derived macrophages (hMDMs) without or with lentiviral overexpression of WT AIP (AIP‐WT) or an AIP carboxy‐terminus deletion mutant (AIPΔCT). c) Dual‐luciferase reporter assay confirming the functionality of the conserved c‐JUN binding site within the human *HM13* promoter using the WT or mutant (MUT) c‐JUN binding site sequence (depicted in red). d) Western blotting analyses confirming lentiviral overexpression of constitutively‐active p38α^D176A/F327S^ or constitutively‐active c‐JUN^S63/73D^ and e) qPCR analysis of *Hm13* expression in hMDMs. f,g) Western blotting analyses in hMDMs and murine bone marrow‐derived macrophages (mBMDMs) without or with lentiviral overexpression of WT AIP (AIP‐WT) or an AIP carboxy‐terminus deletion mutant (AIPΔCT). h) Western blotting analyses confirming lentiviral overexpression of Hm13 in mBMDMs. i,j) mBMDMs without or with lentiviral overexpression of AIP‐WT, AIPΔCT, and/or Hm13 were analyzed after incubation with DMSO vehicle or oxLDL (25 µg/ml) for 24 h. Intracellular content of i) total cholesterol (TC), unesterified free cholesterol (FC), cholesteryl esters (CE) as well as j) triglycerides. k–m) *Hm13* ^fl/fl^, *Hm13* ^mKO^, and *Hm13* ^mOE^ mBMDMs were analyzed after incubation with DMSO vehicle, nLDL (25 µg/ml), or oxLDL (25 µg/ml) for 24 h. Intracellular content of k) total cholesterol (TC), unesterified free cholesterol (FC), cholesteryl esters (CE), and l) triglycerides. m) Representative images and quantitation of Oil Red O‐staining in *Hm13* ^fl/fl^, *Hm13* ^mKO^, and *Hm13* ^mOE^ mBMDMs (scale bar, 50 µm). *n* = 6 independent biological replicates per cohort. Data expressed as means ± SDs compared using b, e–j) one‐way ANOVA and c, k–m) two‐way ANOVA. ^*^ *p* < 0.05, ^**^ *p* < 0.01.

Given the negative relationship between AIP and HM13/SPP expression, we hypothesized that AIP may downregulate HM13/SPP expression in macrophages. AIP, in combination with the chaperone protein HSP90 and its co‐chaperone PTGES3/p23, binds to AHR (**Figure** **3**a), thereby affecting AHR's folding, localization, and activity.^[^ ^14^ ^]^ AHR is a ligand‐activated transcription factor but activates foamy macrophage‐inducing p38 mitogen‐activated protein kinase (p38 MAPK) signaling in macrophages by non‐canonical cross‐talk.^[^ ^15^ ^]^ Our *HM13* promoter analyses did not identify any strongly‐conserved binding sites for AHR, its heterodimer aryl hydrocarbon receptor nuclear translocator (ARNT), or the p38‐responsive transcription factor PPARγ.^[^ ^15b^ ^]^ However, we did unveil a strongly‐conserved binding site for the p38‐responsive transcription factor c‐JUN (Matrix ID: MA0489.1) ≈250 bp upstream of the *HM13* transcriptional start site (TSS) (Figure S14a,b, Supporting Information). Although JNK is the primary kinase of c‐JUN, p38 also phosphorylates c‐JUN in myeloid cells.^[^ ^16^ ^]^

Therefore, we hypothesized that AIP's interaction with AHR may downregulate macrophage HM13/SPP expression via inhibiting p38‐c‐JUN signaling. To test this, we employed lentiviral overexpression of WT AIP (AIP‐WT) or an AIP carboxy‐terminus deletion mutant (AIPΔCT) that cannot bind to AHR^[^ ^17^ ^]^ in non‐polarized hMDMs exposed to oxLDL for 24 h. As postulated, AIP‐WT (but not AIPΔCT) inhibited the phosphorylation of p38 and c‐JUN but did not impact JNK phosphorylation (Figure 3b). As postulated, AIP‐WT (but not AIPΔCT) inhibited transactivation of the *HM13* promoter bearing the WT c‐JUN binding site but not the mutant c‐JUN binding site (Figure 3c). AIP‐WT (but not AIPΔCT) also inhibited *HM13* mRNA expression, which was rescued by either constitutively‐active p38α^D176A/F327S^ or constitutively‐active c‐JUN^S63/73D^ (Figure 3d,e). Consistently, AIP‐WT (but not AIPΔCT) downregulated HM13/SPP protein expression in oxLDL‐treated hMDMs and mBMDMs (Figure 3f,g). Consistent with previous studies,^[^ ^18^ ^]^ neither AIP‐WT nor AIPΔCT significantly affected AHR protein levels; this phenomenon has been attributed to PTGES3/p23's stabilizing effect on AHR.^[^ ^18b^ ^]^ Most notably, AIP‐WT (but not AIPΔCT) reduced total cholesterol, cholesteryl ester, and triglyceride accumulation in oxLDL‐treated mBMDMs, which was rescued by the addition of lentiviral HM13/SPP overexpression (Figure 3h–j). In sum, AIP's interaction with AHR inhibits p38‐c‐JUN‐mediated *HM13* transactivation and resulting in macrophage lipid accumulation.

### 2.4 Macrophage HM13/SPP Enhances oxLDL‐Induced Foamy Macrophage Formation and Pro‐Inflammatory Properties

To discover whether HM13/SPP enhances foamy macrophage formation, we created mice expressing WT levels (*Hm13* ^fl/fl^), low macrophage HM13/SPP levels (*Hm13* ^mKO^ [*Hm13* myeloid knockout]), or high macrophage HM13/SPP levels (*Hm13* ^mOE^ [*Hm13* myeloid overexpression]) (Figure S15a,b, Supporting Information). All transgenic mice were viable and bred normally. As anticipated, HM13/SPP mRNA and protein levels were significantly lower in mBMDMs from *Hm13* ^mKO^ mice, and significantly higher in those from *Hm13* ^mOE^ mice, relative to their *Hm13* ^fl/fl^ littermates (Figure S16a,b, Supporting Information). As the lysozyme Cre driver *Lyz2‐2A* ^Cre^ is active in myeloid‐lineage cells (i.e., monocytes, macrophages, and granulocytes),^[^ ^19^ ^]^ HM13/SPP protein levels were also modulated in circulating monocytes and bone‐marrow‐derived granulocytes (Figure S16c–f, Supporting Information). We confirmed higher GFP expression in monocytic myeloid lineage (Cd11b^+^/Ly6c^+^) cells^[^ ^20^ ^]^ isolated from *Hm13* ^mOE^ murine blood and bone marrow (Figure S17a, Supporting Information). Myeloid *Hm13* modulation did not significantly impact overall white blood cell counts, individual WBC counts (Figure S17b, Supporting Information), adipose tissue F4/80^+^ macrophage counts (Figure S17c, Supporting Information), or liver tissue F4/80^+^ macrophage counts (Figure S17d, Supporting Information).

We incubated non‐polarized *Hm13* ^fl/fl^, *Hm13* ^mKO^, or *Hm13* ^mOE^ mBMDMs with nLDL or oxLDL for 24 h. oxLDL‐treated *Hm13* ^mOE^ mBMDMs displayed substantial increases in total cholesterol, cholesteryl ester, and triglyceride accumulation, while oxLDL‐treated *Hm13* ^mKO^ displayed the opposite (Figure 3k,l). This was corroborated by visible changes in lipid accumulation by Oil Red O staining (Figure 3m). However, modulating HM13/SPP expression did not impact HDL‐mediated cholesterol efflux (Figure S18a, Supporting Information), gene expression of the cholesterol efflux transporters ATP binding cassette A1 and G1 (*Abca1*, *Abcg1*) (Figure S18b, Supporting Information), nor expression of the cholesterol metabolism genes *Acat1*, *Adrp* (*Plin2*), *Cd36*, *Lxra*, and *Plin1* (Figure S18c, Supporting Information) in mBMDMs.

As changes in lipid metabolism can impact inflammatory mediators in macrophages,^[^ ^21^ ^]^ we analyzed the gene expression and secretion of inflammatory cytokines in non‐polarized *Hm13* ^fl/fl^, *Hm13* ^mKO^, or *Hm13* ^mOE^ mBMDMs incubated with oxLDL for 24 h. Notably, *Hm13* ^mOE^ mBMDMs displayed increased expression and secretion of IL‐6, MCP‐1, and TNF‐α, while *Hm13* ^mKO^ displayed the opposite (Figure S19a,b, Supporting Information). This evidence indicates macrophage HM13/SPP enhances oxLDL‐induced foamy macrophage formation and pro‐inflammatory properties.

### 2.5 Myeloid HM13/SPP Increases Plaque Burden and Foamy Macrophage Load in Two Murine Models of Hyperlipidemic Atherosclerosis

**Figure 4:**
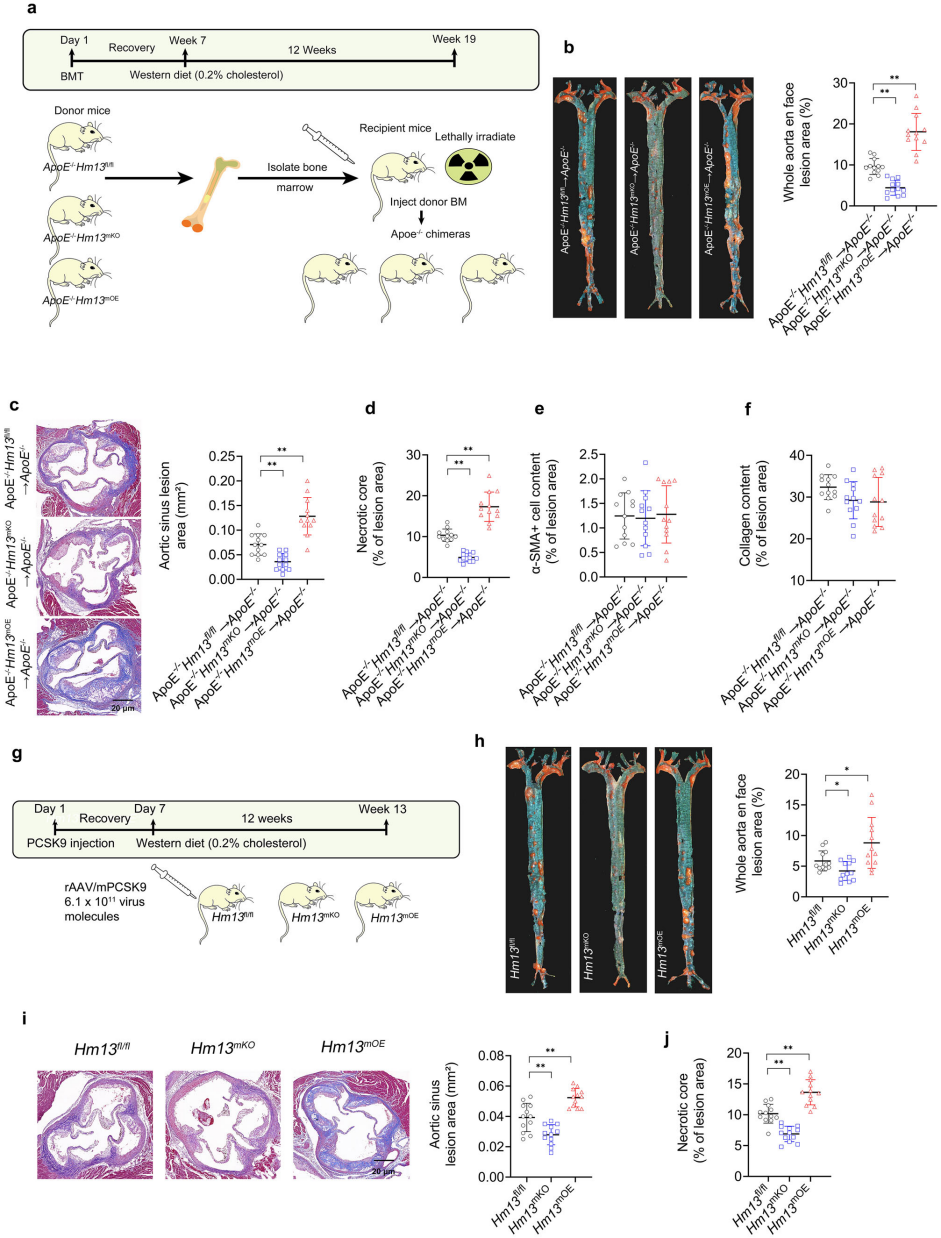
Myeloid HM13/SPP Increases Atherosclerotic Burden in Two Murine Models of Atherosclerosis. a) Schematic of the bone marrow transplant experiments depicted in panels b–f. Bone marrow was extracted from *ApoE* ^−/−^ *Hm13* ^fl/fl^, *ApoE* ^−/−^ *Hm13* ^mKO^, or *ApoE* ^−/−^ *Hm13* ^mOE^ mice and then transplanted into *ApoE* ^−/−^ recipients to form chimeras. b) Representative images of Oil Red O‐stained aortas (week 19) from specified chimeras. Lesion area data is expressed as a % of the total surface area of the aorta. c) Representative images and quantified areas of aortic sinus lesions. d) Necrotic core sizes, e) VSMC (α‐SMA+) cell content, and f) collagen content (Martius Scarlet Blue) in aortic sinus samples. g) Schematic of the rAAV8‐*Pcsk9* mouse model experiments depicted in panels h–j. h) Representative images of oil‐red O‐stained aortas. Lesion area data is expressed as a % of the total surface area of the aorta. i) Representative images and quantified areas of aortic sinus lesions. j) Necrotic core sizes in aortic sinus samples. *n* = 12 mice per cohort. Data expressed as medians ± upper/lower quartiles compared using the Kruskal–Wallis test. ^*^ *p* < 0.05, ^**^ *p* < 0.01.

Bone marrow from *ApoE* ^−/−^ *Hm13* ^fl/fl^, *ApoE* ^−/−^ *Hm13* ^mKO^, or *ApoE* ^−/−^ *Hm13* ^mOE^ mice was transplanted into lethally‐irradiated, 12–13 week‐old *ApoE* ^−/−^ male mice (**Figure** **4**a). These mice were given seven weeks on a standard chow diet to recover from the transplantation procedure and were then fed a hyperlipidemic Western diet containing 0.2% cholesterol for 12 weeks. At sacrifice, there were no significant changes in plasma cholesterol or triglyceride levels between the chimeric cohorts (Figure S20a–d, Supporting Information). Whole aortas of *ApoE* ^−/−^ *Hm13* ^mKO^→*ApoE* ^−/−^ chimeras exhibited less atherosclerosis, and those of *ApoE* ^−/−^ *Hm13* ^mOE^→*ApoE* ^−/−^ chimeras exhibited enhanced atherosclerosis, relative to *ApoE* ^−/−^ *Hm13* ^fl/fl^ controls (Figure 4b). Lesions and necrotic core sizes in the aortic sinus were, on average, smaller in *ApoE* ^−/−^ *Hm13* ^mKO^→*ApoE* ^−/−^ mice and larger in *ApoE* ^−/−^ *Hm13* ^mOE^→*ApoE* ^−/−^ mice relative to *ApoE* ^−/−^ *Hm13* ^fl/fl^ controls (Figure 4c,d). However, VSMC (α‐SMA+) content and plaque collagen content were similar among the three cohorts (Figure 4e,f).

We created a Proprotein Convertase Subtilisin/Kexin Type 9 (*Pcsk9*)‐induced low‐density lipoprotein receptor (LDLR) knockdown model of hyperlipidemic atherosclerosis^[^ ^22^ ^]^ to verify that HM13/SPP accelerates atherogenesis. *Hm13* ^fl/fl^, *Hm13* ^mKO^, or *Hm13* ^mOE^ mice were injected with an adeno‐associated viral vector encoding murine *Pcsk9* (rAAV8‐*Pcsk9*) or empty control viral vector (rAAV8‐Ctrl) followed by Western diet feeding for 12 weeks (Figure 4g). Across all three mouse cohorts, rAAV8‐*Pcsk9* produced similar reductions in LDLR protein levels (Figure S21a, Supporting Information) and similar degrees of hyperlipidemia (Figure S21b–e, Supporting Information). Whole aortas of *Hm13* ^mKO^ rAAV8‐*Pcsk9* mice exhibited less atherosclerosis, and those of *Hm13* ^mOE^ rAAV8‐*Pcsk9* mice exhibited enhanced atherosclerosis, relative to *Hm13* ^fl/fl^ rAAV8‐*Pcsk9* controls (Figure 4h). Lesions and necrotic core sizes in the aortic sinus were, on average, smaller in *Hm13* ^mKO^ rAAV8‐*Pcsk9* mice and larger in *Hm13* ^mOE^ rAAV8‐*Pcsk9* relative to *Hm13* ^fl/fl^ rAAV8‐*Pcsk9* controls (Figure 4i,j).

**Figure 5:**
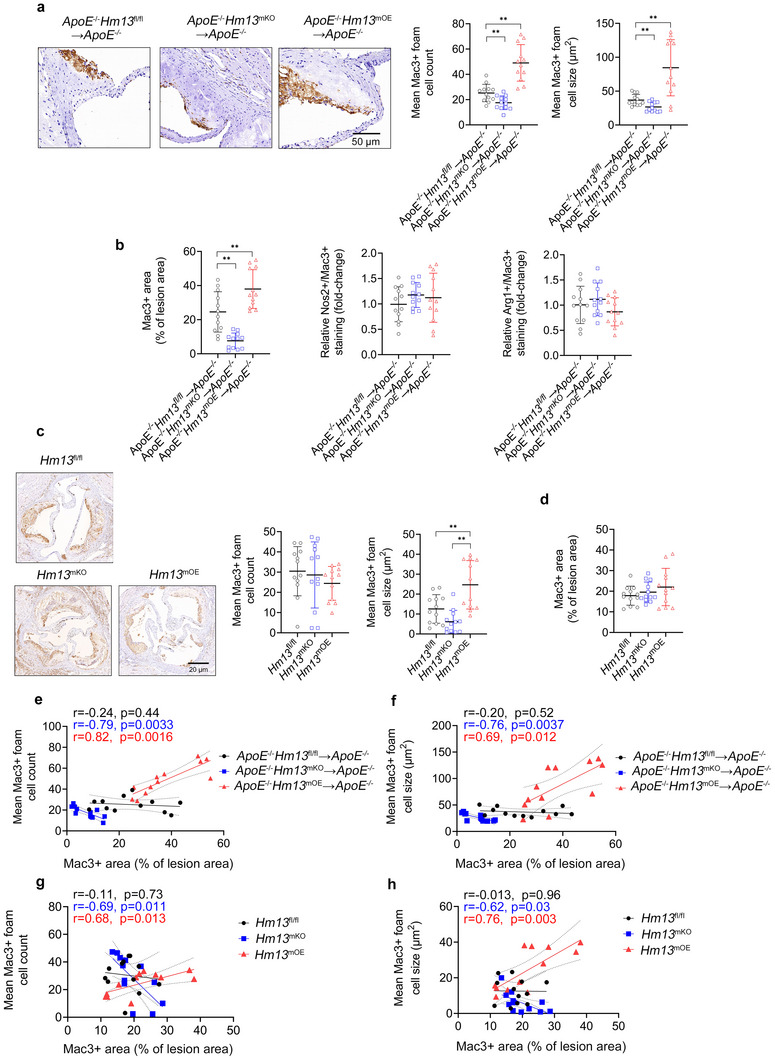
Myeloid HM13/SPP Enhances Foamy Macrophage Accumulation in Atherosclerotic Plaques. a) Representative images of *ApoE* ^−/−^ chimera aortic sinus lesions stained with an anti‐Mac3 antibody (brown). Note the presence of foamy macrophages within the plaque region. Scale bar, 50 µm. Quantification of relative Mac3^+^ foamy macrophage numbers and sizes in *ApoE* ^−/−^ chimera aortic sinus lesions. b) *ApoE* ^−/−^ chimera aortic sinus lesions were stained with antibodies against Mac3, Nos2 (M1 marker), and Arg1 (M2 marker) for relative quantification. c,d) Representative images of aortic sinus lesions (scale bar, 20 µm) from rAAV8‐*Pcsk9* model mice stained with Elastic van Gieson and an anti‐Mac3 antibody (brown). Quantification of c relative Mac3^+^ foamy macrophage numbers and sizes as well as d Mac3 staining in aortic sinus lesions. e–h) In e, f) *ApoE* ^−/−^ chimera aortic sinus lesions and g,h) rAAV8‐*Pcsk9* model aortic sinus lesions, Pearson correlations between Mac3 staining (*x*‐axis) and e,g) foamy macrophage counts (*y*‐axis) and f,h) mean foamy macrophage size (*y*‐axis). Mac3 staining is expressed as a % of the total area of the aortic sinus lesion. *n* = 12 mice per cohort. Data expressed as medians ± upper/lower quartiles compared using a–d) Kruskal–Wallis test. ^*^ *p* < 0.05, ^**^ *p* < 0.01.

Given macrophage HM13/SPP's enhancement of oxLDL‐induced foamy macrophage formation in vitro, we investigated the foamy macrophage load of aortic sinus plaques (i.e., counts and sizes of plaque foamy macrophages) from the two aforementioned mouse models. *ApoE* ^−/−^ *Hm13* ^mKO^→*ApoE* ^−/−^ mice displayed lesser foamy macrophage loads and smaller overall Mac3^+^ immunoreactive areas, while *ApoE* ^−/−^ *Hm13* ^mOE^→*ApoE* ^−/−^ mice displayed greater foamy macrophage loads and overall Mac3^+^ immunoreactive areas when compared to *ApoE* ^−/−^ *Hm13* ^fl/fl^→*ApoE* ^−/−^ mice (**Figure** **5**a,b). However, there were no significant differences in M1 or M2 polarization markers among the three cohorts. *Hm13* ^mKO^ rAAV8‐*Pcsk9* mice displayed smaller foam sizes, while *Hm13* ^mOE^ rAAV8‐*Pcsk9* mice displayed larger foamy macrophage sizes when compared to *Hm13* ^fl/fl^ rAAV8‐*Pcsk9* mice (Figure 5c). In contrast to the *ApoE* ^−/−^ chimeric model, myeloid *Hm13* modulation in the rAAV8‐*Pcsk9* model produced no significant effects on foamy macrophage counts or overall Mac3^+^ immunoreactive areas (Figure 5c,d). Aortic sinus plaques from *ApoE* ^−/−^ *Hm13* ^mKO^→*ApoE* ^−/−^ chimeras showed a negative correlation, while those from *ApoE* ^−/−^ *Hm13* ^mOE^→*ApoE* ^−/−^ chimeras showed a positive correlation, between Mac3^+^ staining area and foamy macrophage count (Figure 5e) as well as between Mac3^+^ staining area and foamy macrophage size (Figure 5f). A similar pattern was observed in aortic sinus plaques from rAAV8‐*Pcsk9* mice (Figure 5g,h). In both murine models, there were no significant correlations observed between total cholesterol levels and either foamy macrophage counts, foamy macrophage sizes, or aortic sinus plaque areas (Figure S22a–d, Supporting Information). In sum, the evidence indicates that macrophage HM13/SPP increases plaque burden and foamy macrophage load in two murine models of hyperlipidemic atherosclerosis.

### 2.6 Macrophage HM13/SPP Cleaves the Metabolic Regulator HO‐1, Thereby Promoting HO‐1 Proteasomal Degradation

**Figure 6:**
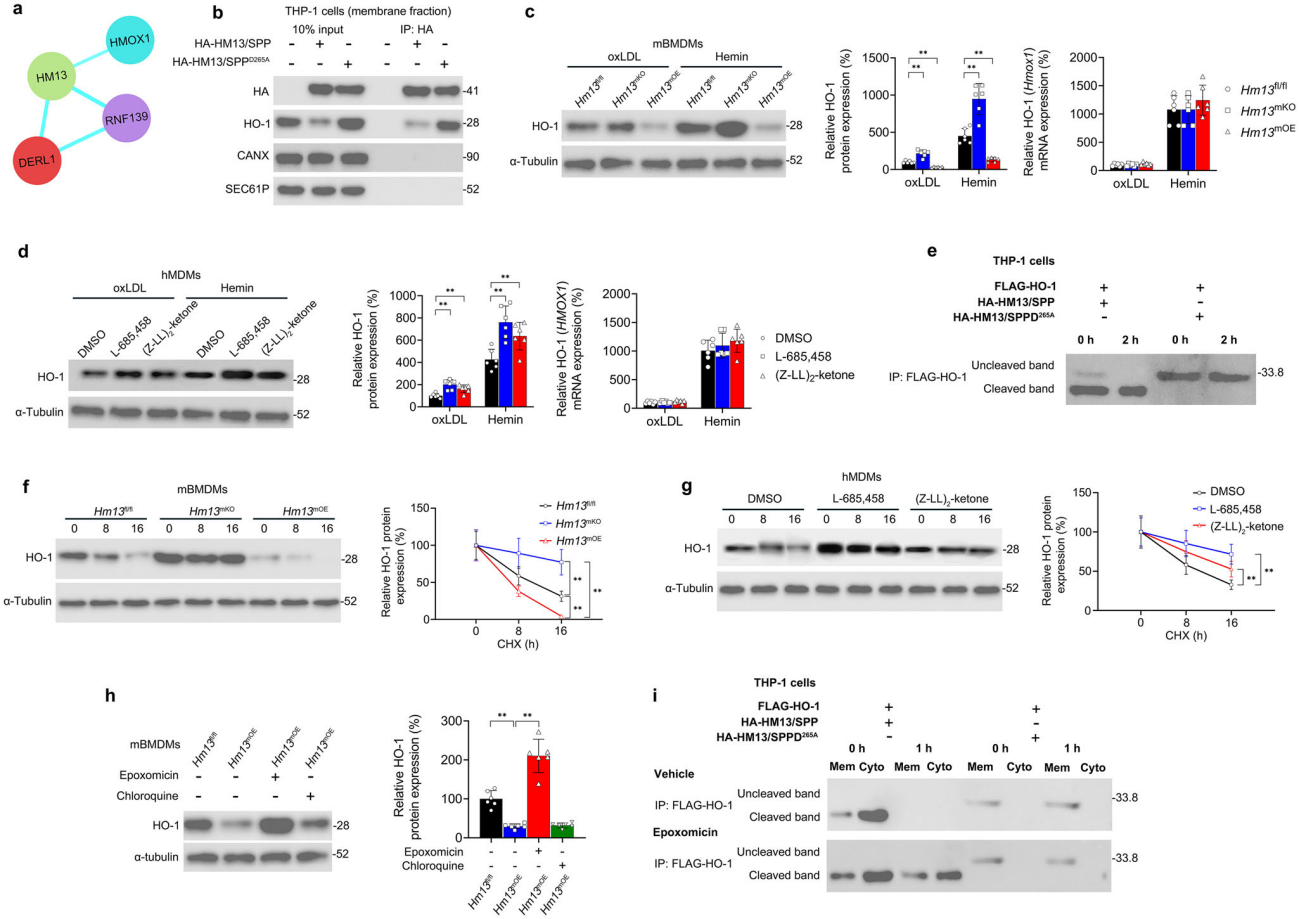
Macrophage HM13/SPP Cleaves the Metabolic Regulator HO‐1, Thereby Promoting HO‐1 Proteasomal Degradation. a) Cytoscape schematic of STRING protein‐protein interaction (PPI) analysis (high confidence = 0.700) showing direct PPIs between the ERAD complex proteins and HO‐1 (HMOX1). Blue edges indicate database evidence. b) Following 24 h hemin (50 µM) stimulation, membrane fraction inputs from transduced THP‐1 cells were immunoprecipitated with an anti‐HA antibody (IP:HA) and then subjected to immunoblotting analysis. c, d) Western blotting analysis of HO‐1 in c) *Hm13* ^fl/fl^, *Hm13* ^mKO^, and *Hm13* ^mOE^ murine bone marrow‐derived macrophages (mBMDMs) and d) HM13/SPP inhibitor‐treated human monocyte‐derived macrophages (hMDMs) following oxLDL (25 µg/ml) or hemin (50 µM) stimulation for 24 h. e) Pulse‐chase analysis of HO‐1 cleavage in transduced THP‐1 cells by HO‐1 immunoprecipitation with an anti‐FLAG antibody (IP:FLAG) at 0 and 2 h followed by SDS‐PAGE autoradiography. f, g) Cycloheximide (CHX, 2 µg/mL) chase studies in f *Hm13* ^fl/fl^, *Hm13* ^mKO^, and *Hm13* ^mOE^ mBMDMs and g) HM13/SPP inhibitor‐treated hMDMs following 24 h hemin (50 µM) stimulation. h) Western blotting analysis of HO‐1 in *Hm13* ^fl/fl^ and *Hm13* ^mOE^ mBMDMs pretreated with vehicle, epoxomicin (1 µM), or chloroquine (50 µM). *n* = 6 independent biological replicates per cohort. i) Pulse‐chase analysis of HO‐1 cleavage as performed in panel e without or with epoxomicin (1 µM). At 0 h and 1 h, post‐nuclear lysates were separated into membrane (Mem) fractions and cytosolic (Cyto) fractions prior to FLAG immunoprecipitation and SDS‐PAGE autoradiography. Data expressed as means ± SDs compared using c, d, h) one‐way ANOVA and f, g) two‐way ANOVA. ^*^ *p* < 0.05, ^**^ *p* < 0.01.

HM13/SPP is an ER‐resident, intramembrane‐cleaving aspartyl protease that directly associates with the metabolic regulator HO‐1 (**Figure** **6**a) and proteolytically cleaves HO‐1 in the ER prior to proteasomal degradation of HO‐1 in HeLa cells.^[^ ^23^ ^]^ Based on this prior evidence, we hypothesized that HM13/SPP may promote cleavage of HO‐1 in macrophages. Co‐immunoprecipitation studies in THP‐1 macrophages transduced with HA‐tagged WT HM13/SPP or the HM13/SPP dominant‐negative mutant HM13/SPP^D265A^ (which binds to but cannot cleave its substrates^[^ ^5^ ^]^) revealed that HM13/SPP bound to HO‐1 but not to the negative control ER membrane proteins CANX and SEC61α (Figure 6b). Notably, HM13/SPP^D265A^ enhanced HO‐1 protein expression relative to WT HM13/SPP, suggesting that HM13/SPP's cleaving activity reduces HO‐1 protein expression. Modulating HM13/SPP expression in mBMDMs inversely affected HO‐1 protein levels following oxLDL or hemin stimulation with no impact on HO‐1 (*Hmox1*) mRNA levels (Figure 6c). Similarly, levels of endogenous HO‐1 in hMDMs following oxLDL or hemin stimulation were enhanced using the HM13/SPP inhibitors L‐685458 and (Z‐LL)2‐ketone with no impact on HO‐1 (*HMOX1*) mRNA levels (Figure 6d).

To assess if HM13/SPP directly cleaves HO‐1 in macrophages, we performed a pulse‐chase analysis in [35S]methionine/cysteine‐radiolabeled THP‐1 macrophages transduced with FLAG‐tagged HO‐1 as well as HA‐tagged WT HM13/SPP or HM13/SPP^D265A^. As HO‐1 cleavage results in a loss of a 10–residue polypeptide fragment, HO‐1 cleavage can be detected by a slight diminution in molecular mass.^[^ ^23^ ^]^ At the 0‐h timepoint in WT HM13/SPP‐overexpressing cells, HO‐1 is detected in its predominantly cleaved form (strong, faster migrating band) with a minimal amount in the uncleaved form (faint, slower migrating band) (Figure 6e). By the 2 h timepoint, only HO‐1′s cleaved form can be detected. In contrast, in HM13/SPP^D265A^‐overexpressing cells, only HO‐1′s uncleaved form can be detected. This evidence suggests that catalytically‐active HM13/SPP cleaves HO‐1 in macrophages.

HM13/SPP's proteolysis of HO‐1 is necessary for its proteasomal degradation in HeLa cells.^[^ ^23^ ^]^ Thus, we assessed whether HM13/SPP promotes proteasomal degradation of HO‐1 in macrophages through cycloheximide (CHX) chase studies. HO‐1 degradation in hemin‐exposed mBMDMs was promoted by *Hm13* ^mOE^ and inhibited by *Hm13* ^mKO^ (Figure 6f). Similarly, HO‐1 degradation in hemin‐exposed hMDMs was inhibited by HM13/SPP inhibition (Figure 6g). Pre‐exposure to the selective proteasome inhibitor epoxomicin^[^ ^24^ ^]^ abrogated *Hm13* ^mOE^ enhancement of HO‐1 degradation in hemin‐exposed mBMDMs, but pre‐exposure to the lysosome pathway inhibitor chloroquine did not produce this effect (Figure 6h). To assess whether HO‐1′s cleavage by HM13/SPP is necessary for its proteasomal degradation, THP‐1 macrophage lysates from the afore described pulse‐chase study were fractionated into cytosolic and membrane fractions in the absence or presence of epoxomicin (Figure 6i). In WT HM13/SPP‐overexpressing cells, HO‐1′s cleavage and release into the cytosolic fraction occurred under both conditions, indicating proteasomal‐independence. However, HO‐1′s cleaved form was not degraded in the presence of epoxomicin, indicating proteasomal‐dependence. In contrast, in HM13/SPP^D265A^‐overexpressing cells, only HO‐1′s uncleaved form was detected in the membrane fraction. These combined findings indicate that HM13/SPP‐mediated HO‐1 cleavage is necessary for HO‐1 proteasomal degradation.

HM13/SPP, in combination with RNF139 (TRC8, an E3 ubiquitin ligase) and DERLIN‐1 (DERL1, an E3 ubiquitin ligase adaptor protein), forms a 500‐kDa SPP ERAD protein complex that targets ER membrane proteins for HM13/SPP‐mediated cleavage and proteasomal degradation in HEK293T cells.^[^ ^5^ ^]^ However, the role(s) of these ERAD proteins in HM13/SPP‐mediated HO‐1 cleavage and subsequent degradation in macrophages remain uncharacterized. Co‐immunoprecipitation studies confirmed that HM13/SPP binds to both RNF139 and DERLIN‐1 in transduced THP‐1 macrophages (Figure S23a, Supporting Information). Moreover, substrate trapping revealed HO‐1 binding to the 500‐kDa SPP ERAD complex (Figure S23b, Supporting Information). CHX chase analysis conducted with co‐expression of the dominant‐negative RNF139 ring finger mutant (RNF139‐RFM^[^ ^25^ ^]^) or the dominant‐negative mutant DERLIN1^G180V^ (which binds to the SPP ERAD complex but cannot function^[^ ^5^ ^]^) revealed both mutants independently inhibited HO‐1 degradation similar to that of HM13/SPP^D265A^ (Figure S23c, Supporting Information). Pulse‐chase analysis in [35S]methionine/cysteine‐radiolabeled THP‐1 macrophages transduced with FLAG‐tagged HO‐1 revealed that RNF139 and DERLIN1 are necessary for HO‐1 cleavage and subsequent degradation (Figure S23d, Supporting Information). This evidence confirms that HM13/SPP associates with RNF139 and DERLIN‐1 to form a 500‐kDa SPP ERAD protein complex and that both RNF139 and DERLIN‐1 are necessary for HM13/SPP‐mediated HO‐1 cleavage and subsequent degradation in macrophages.

### 2.7 Macrophage HM13/SPP Enhances Foamy Macrophage Formation and Atherosclerosis Through HO‐1 Degradation

**Figure 7:**
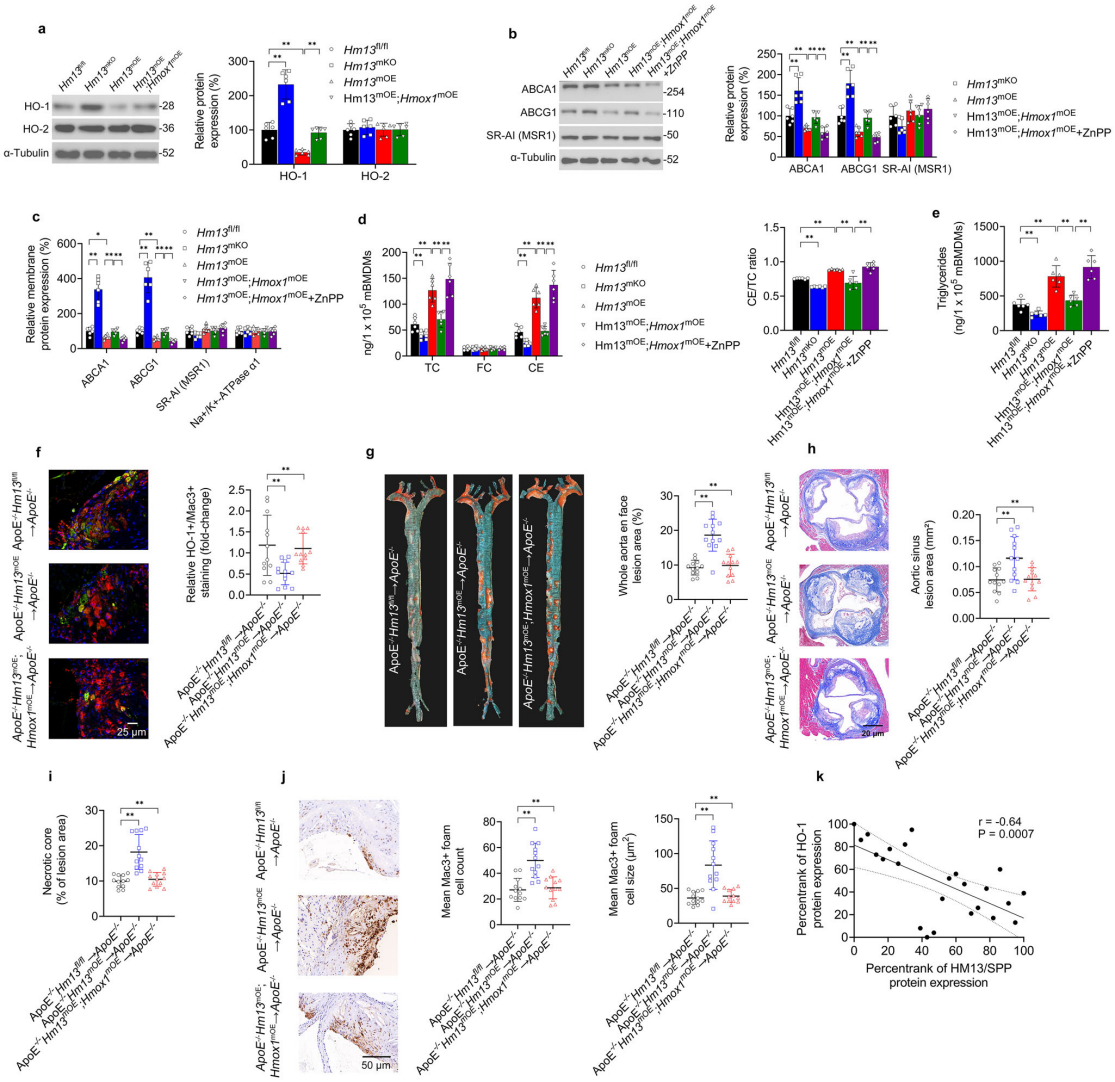
Macrophage HM13/SPP Enhances Foamy Macrophage Formation and Atherosclerosis Through HO‐1 Degradation. a, b) *Hm13* ^fl/fl^, *Hm13* ^mKO^, *Hm13* ^mOE^, and *Hm13* ^mOE^; *Hmox1* ^mOE^ murine bone marrow‐derived macrophages (mBMDMs) were subjected to the following experiments after incubation with oxLDL (25 µg/ml) without or with ZnPP (10 µM) for 24 h. a, b) Western blotting analyses of a) the heme oxygenases HO‐1 and HO‐2 as well as b) the cholesterol transporters ABCA1, ABCG1, and SR‐AI. c) Membrane‐fraction ELISA of ABCA1, ABCG1, SR‐AI, and the membrane control protein Na^+^/K^+^‐ATPase α1. d, e) Intracellular content of d) total cholesterol (TC), unesterified free cholesterol (FC), and cholesteryl esters (CE) and e) triglycerides in mBMDMs after incubation with DMSO vehicle or oxLDL (25 µg/ml) for 24 h. f–j) Aortic sinus lesions were isolated from *ApoE* ^−/−^ *Hm13* ^fl/fl^→*ApoE* ^−/−^, *ApoE* ^−/−^ *Hm13* ^mOE^→*ApoE* ^−/−^, and *Hm13* ^mOE^;*Hmox1* ^mOE^→*ApoE* ^−/−^ mice. f) Representative immunofluorescent images of *ApoE* ^−/−^ chimera aortic sinus lesions stained with an anti‐HO‐1 antibody (green) and anti‐Mac3 antibody (red) (scale bar, 25 µm) and quantification of HO‐1^+^/Mac3^+^ staining (yellow). g) Representative images of Oil Red O‐stained aortas (week 19) from specified chimeras. Lesion area data is expressed as a % of the total surface area of the aorta. h) Representative images and quantified areas of lesion sizes and i) necrotic core sizes in aortic sinus samples. j) Representative images of *ApoE* ^−/−^ chimera aortic sinus lesions stained with an anti‐Mac3 antibody (brown). Scale bar, 50 µm. Quantification of relative Mac3^+^ foamy macrophage numbers and sizes in *ApoE* ^−/−^ chimera aortic sinus lesions. k) ELISA and Pearson correlation analyses of AIP and HM13/SPP protein expression in ex vivo carotid plaque foamy macrophages. Data expressed as means ± SDs for a‐e) one‐way ANOVA and medians ± upper/lower quartiles compared using f–j) Kruskal–Wallis test. ^*^ *p* < 0.05, ^**^ *p* < 0.01.

As HO‐1 promotes oxLDL‐induced foamy macrophage formation through regulating macrophage cholesterol transporter expression,^[^ ^26^ ^]^ we hypothesized that HM13/SPP's enhancement of HO‐1 degradation may promote foamy macrophage formation through modulating cholesterol transporter expression. In oxLDL‐exposed mBMDMs, total protein levels of HO‐1 as well as total and membrane protein levels of Abca1 and Abcg1 were downregulated by *Hm13* ^mOE^ and upregulated by *Hm13* ^mKO^ (**Figure** **7**a–c). Accordingly, total cholesterol, cholesterol ester, and triglyceride levels were promoted by *Hm13* ^mOE^ and inhibited by *Hm13* ^mKO^ (Figure 7d,e). The addition of myeloid HO‐1 overexpression (*Hmox1* ^mOE^) rescued the effects of *Hm13* ^mOE^, and the HO‐1 inhibitor zinc protoporphyrin (ZnPP) validated these *Hmox1* ^mOE^ rescue effects as HO‐1‐specific. Notably, mRNA levels of *Abca1* and *Abcg1* were not significantly impacted by *Hm13* modulation or HO‐1 overexpression (Figure S24a,b, Supporting Information), suggesting that the HM13/SPP‐HO‐1 axis downregulates Abca1 and Abcg1 through a post‐translational mechanism. The addition of *Hmox1* ^mOE^ also rescued the effects of *Hm13* ^mOE^ on inflammatory cytokine release in oxLDL‐exposed mBMDMs (Figure S25a–c, Supporting Information). In vivo, aortic sinus plaque macrophage HO‐1 expression in the *ApoE* ^−/−^ chimeric model was downregulated by *Hm13* ^mOE^ and rescued by the addition of *Hmox1* ^mOE^ (Figure 7f). Notably, the enhanced atherogenesis and foamy macrophage load observed in *ApoE* ^−/−^ *Hm13* ^mOE^→*ApoE* ^−/−^ mice was lost by the addition of *Hmox1* ^mOE^ (Figure 7g–j). To extend these findings to human atherosclerosis, foamy macrophages were isolated from human carotid artery plaques for 24 h ex vivo culture and ELISA (n = 8 donors, three ex vivo cultures per donor). Notably, HO‐1 protein expression negatively correlated with HM13/SPP protein expression (Figure 7k). This evidence suggests that macrophage HM13/SPP's effects on cholesterol transporter expression, foamy macrophage generation, and atherogenesis are dependent on HO‐1 degradation.

### 2.8 Macrophage HM13/SPP Induces Phenotypic Changes in Co‐Cultured VSMCs Through HO‐1 Degradation

Given that the HM13/SPP‐HO‐1 axis modulates oxLDL‐induced inflammatory cytokine release by macrophages, we hypothesized that the macrophage HM13/SPP‐HO‐1 axis may impact the macrophage's interaction with other vascular cell types. In particular, VSMCs localize near macrophages within the subintimal plaque layer,^[^ ^27^ ^]^ and macrophages have been shown to induce phenotypic changes in VSMCs that may contribute to plaque vulnerability, such as aberrant production of matrix metalloproteases (MMPs), inflammatory cytokines, and extracellular matrix proteins.^[^ ^28^ ^]^ Here, we employed a macrophage‐VSMC Transwell co‐culture system in which oxLDL‐treated mBMDMs (Transwell bottom chamber) and murine VSMCs (Transwell upper chamber) were co‐cultured for 72 h. oxLDL‐treated *Hm13* ^fl/fl^ mBMDMs induced several phenotypic changes in VSMCs, namely: upregulation of MMP‐9 expression and activity (Figure S26a–c, Supporting Information), upregulation of the inflammatory cytokines IL‐1β and IL‐6 (Figure S26d, Supporting Information), and downregulation of the extracellular matrix proteins collagen I, III, and elastin (Figure S26e, Supporting Information). Notably, oxLDL‐treated *Hm13* ^mOE^ mBMDMs potentiated these phenotypic changes in VSMCs, while oxLDL‐treated *Hm13* ^mKO^ abrogated these effects. The addition of *Hmox1* ^mOE^ rescued the effects of *Hm13* ^mOE^, and the HO‐1 inhibitor ZnPP validated these *Hmox1* ^mOE^ rescue effects as HO‐1‐specific. These findings indicate that the macrophage HM13/SPP‐HO‐1 axis induces phenotypic changes in VSMCs that may contribute to plaque vulnerability.

## 3 Discussion

Hypercholesterolemia, clinically defined as chronically elevated LDL‐C (>4.1 mM) and/or non‐HDL‐C levels (>4.9 mM),^[^ ^29^ ^]^ has been positively associated with an increased risk of atherosclerotic disease.^[^ ^30^ ^]^ Hypercholesterolemia activates circulating monocytes and enhances their migration into the vascular wall, where they uptake oxLDL via scavenger receptors.^[^ ^30^ ^]^ This exposure to oxLDL induces expression of the macrophage differentiation marker CD68 and a higher phagocytic capacity, which culminates in atherogenic foamy macrophage formation.^[^ ^30^ ^]^ Here, our WGCNA and scRNAseq analyses revealed that the atherosclerosis‐associated RGN driver gene *AIP* was negatively correlated with *HM13* in human plaques and foamy macrophages, respectively. AIP is a key chaperone of AHR, a ligand‐activated transcription factor that responds to various atherogenic environmental ligands including oxLDL, 2,3,7,8‐Tetrachlorodibenzo‐p‐dioxin (TCDD), urban dust particles (UDP), and diesel exhaust particulate (DEP).^[^ ^31^ ^]^ Indeed, AHR agonism by oxLDL and TCDD stimulates foamy macrophage formation in U937 macrophages.^[^ ^32^ ^]^ In its non‐canonical signal regulator role, AHR activates foamy macrophage‐inducing p38 MAPK activity in macrophages.^[^ ^15^ ^]^ We discovered that AIP, through its interaction with AHR, downregulates p38‐c‐JUN‐mediated HM13/SPP transactivation. Consistently, AIP overexpression inhibited oxLDL‐induced lipid accumulation in macrophages, which was rescued by HM13/SPP overexpression. We also discovered strong, positive correlations between plaque Mac3^+^ staining areas and foamy macrophage counts/areas in both murine *Hm13* ^mOE^ atherosclerotic models but not in the *Hm13* ^fl/fl^ or *Hm13* ^mKO^ models, suggesting that myeloid *Hm13* overexpression contributes to foamy macrophage generation and growth. In sum, AIP's interaction with AHR downregulates macrophage HM13/SPP, a driver of oxLDL‐induced lipid accumulation and foamy macrophage generation.

Foamy macrophage generation is based on an imbalance favoring modified LDL influx and endogenous lipid synthesis over free cholesterol efflux and lipid catabolism.^[^ ^33^ ^]^ Given that i) modulating HM13/SPP expression did not impact HDL‐mediated cholesterol efflux, free cholesterol levels, or known atherosclerosis‐associated cholesterol metabolism genes and ii) HM13/SPP is an aspartic intramembrane protease linked to cholesterol‐dependent ERAD,^[^ ^34^ ^]^ we reasoned that HM13/SPP likely promotes endogenous lipid synthesis through an ERAD‐based mechanism. We demonstrated that HM13/SPP downregulates cholesterol efflux transporter expression and enhances foamy macrophage formation and atherosclerosis via promoting ERAD‐mediated HO‐1 proteasomal degradation. Our findings are consistent with previous studies demonstrating HO‐1′s role in the atheroprotective response. Specifically, macrophage HO‐1 has been shown to reduce levels of reactive oxygen species (ROS), inflammatory cytokines, oxLDL‐induced foamy macrophage formation, and plaque macrophage content in murine models.^[^ ^26^ ^]^ Moreover, HO‐1 induction has been shown to reduce necrotic core size and intraplaque lipid accumulation in murine atherosclerotic plaques.^[^ ^35^ ^]^ Although other HM13/SPP substrates may play a role in atherogenesis and require further investigation, the present findings support proteasomal degradation of HO‐1 as a key underlying mechanism by which macrophage HM13/SPP drives oxLDL‐induced lipid accumulation and foamy macrophage generation.

In terms of the mechanism underlying HO‐1 proteasomal degradation in macrophages, we show that HM13/SPP associates with the ERAD proteins RNF139 (TRC8, an E3 ubiquitin ligase) and DERLIN‐1 (DERL1, an E3 ubiquitin ligase adaptor protein) to form a 500‐kDa SPP ERAD protein complex and that both RNF139 and DERLIN‐1 are necessary for HM13/SPP‐mediated HO‐1 cleavage and subsequent degradation. Chen et al.’s work suggests that DERLIN‐1 may act as a receptor for HM13/SPP substrates and that RNF139 may ubiquitinate these substrates to enable their proteasomal degradation following cleavage.^[^ ^5^ ^]^ Further research is needed to determine if HO‐1 degradation abides by this pathway in macrophages.

## 4 Conclusion

AIP's interaction with AHR downregulates macrophage HM13/SPP, a driver of oxLDL‐induced lipid accumulation, foamy macrophage generation, and atherogenesis. Therefore, the development of therapeutics to target the macrophage AIP‐HM13/SPP axis may show promise in combating atherogenic foamy macrophage formation. Further investigation is needed to examine whether targeting the macrophage AIP‐HM13/SPP axis promotes atheroma regression or later‐stage plaque stability.

## 5 Experimental Section

### Ethics Statement

All protocols involving human participants were approved in advance by the Ethics Committee of the First People's Hospital of Yunnan (approval no. KHLL2024‐KY220). Human blood and tissue samples were collected in accordance with the Declaration of Helsinki. Written informed consent was received from all participants prior to donation. All animal studies were approved in advance by the Animal Care and Use Committee of Kunming Medical University (approval no. kmmu20241541) and were performed in accordance with the NIH Guide for the Care and Use of Laboratory Animals.

### Data and Materials Availability

The publicly available data employed in this study can be accessed in the GEO database under the accession nos. GSE40231, GSE11138, and GSE224273. The code used for data analysis is available at our GitHub repository: https://github.com/rzhuang‐cqmu/macrophage‐HM13. The remaining data generated in this study are provided in the main text or the Supporting Information file.

### Animal Housing and Euthanasia Procedures

All mice were housed in individually‐ventilated cages with a 12 h light/dark cycle at 22 °C. Unless otherwise specified, mice were fed a standard chow diet and filtered water ad libitum. Mice were humanely euthanized by 4% isoflurane inhalation followed by cervical dislocation.

### In Silico Analyses

See methods (Supporting Information) for details regarding the WGCNA, single‐cell RNAseq (scRNAseq), and promoter analyses.

### Collection and Analyses of Human Coronary Artery and Carotid Plaque Specimens

Human coronary artery specimens were obtained from twenty cadavers following fatal acute ST‐elevation myocardial infarction (STEMI). The clinicodemographic characteristics of this cohort are provided in Table S1 (Supporting Information). For each donor, two coronary plaque samples (plaque burden >25%) and one normal coronary sample were obtained for FACS and quantitative reverse transcription PCR (qPCR). See methods (Supporting Information) for inclusion/exclusion criteria and detailed procedures.

Human atheromatous carotid plaque specimens were obtained from ten carotid endarterectomy patients. The clinicodemographic characteristics of this cohort are provided in Table S2 (Supporting Information). Two specimens were randomly selected for immunofluorescent staining. Each of the remaining eight specimens was divided into three parts; these 24 plaque samples were used for ex vivo foamy macrophage culture and ELISA. See methods (Supporting Information) for inclusion/exclusion criteria and detailed procedures.

### Generation and Packaging of Lentiviral Vectors

See Methods (Supporting Information).

### Isolation and Transduction of Human Monocyte‐Derived Macrophages (hMDMs) and Murine Bone Marrow‐Derived Macrophages (mBMDMs)

See Methods (Supporting Information).

### Construction and Characterization of Myeloid‐Specific Transgenic Hm13 Murine Models

See Methods (Supporting Information).

### Myeloid Green Fluorescent Protein (GFP) Quantification

See Methods (Supporting Information).

### Murine Models of Atherosclerosis

For the bone marrow transplantation model,^[^ ^36^ ^]^ *ApoE* ^−/−^ *Hm13* ^fl/fl^, *ApoE* ^−/−^ *Hm13* ^mKO^, or *ApoE* ^−/−^ *Hm13* ^mOE^ mice (male and female, 12–13 weeks old) were euthanized by cervical dislocation. Bone marrow cells from femur/tibiae were isolated using a standard protocol and resuspended in Hank's Balanced Salt Solution (HBSS, Thermo Fisher Scientific) containing 10% (v/v) ultralow endotoxin FBS. A total of 4 × 10^6^ cells were then tail‐vein injected into *ApoE* ^−/−^ mice (males, 12–13 weeks old). The recipient mice were irradiated with 11 Grays (Gy) in two equal doses of 5.5 Gy separated by a period of 4 h during the day of transplantation. The chimeric mice were given sterile water acidified by 1.1% (v/v) HCl till the end of the procedure. Following a seven‐week recovery period on a standard chow diet, the chimeric mice were switched to a Western diet (12% fat and 0.2% cholesterol; Keao Xieli Feed) for 12 weeks.

For the construction of the Proprotein Convertase Subtilisin/Kexin 9 (*Pcsk9*) murine model,^[^ ^36^ ^]^ an adeno‐associated virus 8 (rAAV8)‐based vector supporting hepatic delivery of the murine *Pcsk9* ^D377Y^ gene was purchased from Vigene Biosciences (Shangdong, China). The mice were injected with 6 × 10^11^ viral particles via a single tail‐vein injection. Following a seven‐day recovery period on a standard chow diet, the mice were fed the aforementioned Western diet for 12 weeks.

### Plasma Lipid Profiling

Plasma total cholesterol (TC), high‐density lipoprotein cholesterol (HDL‐C), and triglycerides were measured on a Roche Cobas 6000 auto‐analyzer. Plasma LDL‐C was calculated using the Friedewald equation.

### Atherosclerotic Plaque Assessment

PBS was perfused through the heart of mice and then 10% (w/v) neutral buffered formalin was added. The aorta was excised, processed, fixed, and stained with Oil Red O (60% [v/v] in isopropanol) as previously described.^[^ ^36^ ^]^ *En face* images were captured by a macroscopic charge‐coupled camera and analyzed using NIS‐Elements software (Nikon).

Aortic sinus samples were excised from the heart, fixed, and serially sectioned (7 µm) from the beginning of the aorta to the valve leaflets as previously described.^[^ ^36^ ^]^ Histological analysis of aortic sinus sections was conducted using a modified Paigen method.^[^ ^37^ ^]^ Trichrome staining was applied to measure aortic sinus lesions and their collagen content. Necrotic core areas within these trichrome‐stained aortic sinus lesions were demarcated based on the presence of two factors: intimal acellularity (i.e., hematoxylin+ nuclei between the lumen and the internal elastic lamina) and cholesterol clefts.^[^ ^38^ ^]^ The necrotic core size was then calculated as a percentage of the aortic sinus lesion area.

For immunohistochemistry, aortic sinus sections were dewaxed, rehydrated, and subjected to heat‐mediated antigen retrieval and non‐specific serum blocking as previously described.^[^ ^36^ ^]^ The details regarding the primary and secondary antibodies are available in Table S3 (Supporting Information). The number and size of foamy macrophages (i.e., Mac3^+^ cells containing vacuolated cytoplasm and lipid droplets) were analyzed using NIS‐Elements software (Nikon). The vascular smooth muscle cell (VSMC) marker α‐SMA, M1 marker Nos2, M2 marker Arg1, and HO‐1 were visualized with fluorescent antibodies. Sections were counterstained with Carazzi's hematoxylin and captured using a Ti Eclipse inverted fluorescence microscope (Nikon). Nos2^+^, Arg1^+^, and HO‐1^+^ staining in Mac3^+^ cells were quantified within Mac3^+^ aortic sinus lesion areas using ImageJ. Briefly, the Nos2^+^Mac3^+^ % (100%×Nos2^+^Mac3^+^ count ÷ total Mac3^+^ count), Arg1^+^Mac3^+^ % (100%×Arg1^+^Mac3^+^ count ÷ total Mac3^+^ count), and HO‐1^+^Mac3^+^ % (100%×HO‐1^+^Mac3^+^ count ÷ total Mac3^+^ count) were calculated for each cohort. The values were then normalized to the control cohort.

### OxLDL Uptake and HDL‐Mediated Cholesterol Efflux

mBMDMs were incubated at 37 °C for 24 h with human oxLDL (25 µg/ml; 5685‐3557, Bio‐Rad). Total cell lipid extraction was performed using 7:11:1 (v/v/v) chloroform: isopropanol: IGEPAL CA‐630 (Sigma–Aldrich). This sample was dried at 50 °C and resuspended in 120 µl of cholesterol assay buffer; total cholesterol, cholesteryl esters, and free cholesterol were then measured using the Cholesterol Quantification Kit (MAK043, Sigma–Aldrich). The assessment of foamy macrophages was done by Oil Red O [60% (v/v) in isopropanol] staining.

To conduct the efflux assays, mBMDMs were incubated for 24 h in Dulbecco's modified Eagle's medium (DMEM) supplemented with 0.2% (w/v) fatty acid‐free bovine serum albumin (FAF‐BSA) and 2.5 µM TopFluor cholesterol (all Sigma–Aldrich). Following medium removal, mBMDMs were washed with PBS and kept for 18 h in DMEM supplemented with 0.2% (w/v) FAF‐BSA. Then, mBMDMs were subjected to a 4 h incubation period with human HDL (50 µg/ml) (Bio‐Rad). Supernatants were collected, and mBMDMs were lysed with 1% (w/v) cholic acid (Sigma–Aldrich) in ethanol. Cholesterol efflux was calculated using a microplate reader (excitation = 490 nm, emission = 520 nm; Infinite M200 Pro, Tecan, Switzerland) as follows: (fluorescence from the supernatant) ÷ (total fluorescence from the supernatant and BMDM lysate) × 100%.

### ELISA Studies in mBMDMs

See Methods (Supporting Information).

### qPCR

Total RNA was isolated with the Total RNA Isolation Kit (ForeGene, Chengdu, China) and reverse‐transcribed to cDNA using an iScript cDNA Synthesis Kit (Bio‐Rad, Shanghai, China). qPCR was conducted using iQ SYBR Green Supermix (Bio‐Rad) in a QuantStudio 3 Real‐Time PCR System (Thermo Fisher). The primer sequences are available in Table S4 (Supporting Information). The 2^−ΔΔCt^ method was employed to calculate fold‐changes in mRNA expression using the housekeeping genes *Actb*/*ACTB* and *Gapdh*/*GAPDH*.

### ERAD Studies

Transduction and differentiation protocols for THP‐1 cells are described in the Methods (Supporting Information). To stimulate HO‐1 expression, mBMDMs, hMDMs, and THP‐1 macrophages were incubated at 37 °C with human oxLDL (25 µg/ml; 5685‐3557, Bio‐Rad) or hemin (50 µM, 51280–1G, Sigma–Aldrich) for 24 h. Cycloheximide (CHX) chase studies were performed following 24 h hemin exposure using CHX (100 µg/ml). At the indicated time points, immunoblotting analysis was performed on whole cell extracts. For inhibitor experiments, L‐658485 (5 µM), (Z‐LL)2‐ketone (50 µM), epoxomicin (1 µM), chloroquine (50 µM) or DMSO vehicle control (all Calbiochem) were added to cell cultures 8 h after the initiation of hemin exposure. Cells were incubated with these inhibitors for a 16 h period prior to experimentation.

### Pulse‐Chase Analyses for HO‐1 Cleavage and Degradation

See Methods (Supporting Information).

### Co‐Immunoprecipitation and Immunoblotting

Co‐immunoprecipitation was performed by applying 2 µg anti‐HA antibodies per 1000 µg whole‐cell protein followed by Protein A/G PLUS‐Agarose bead incubation (ThermoFisher) as previously described.^[^ ^39^ ^]^ For immunoblotting, 20 µg of total protein lysates were separated on 4–12% NuPAGE Bis‐Tris gels (Invitrogen) and subjected to standard immunoblotting procedures. The details regarding the primary and secondary antibodies are available in Table S5 (Supporting Information). Band detection was performed using an ECL kit (ThermoFisher) and a ChemiScope3600 Mini chemiluminescence imaging system (Clinx Science Instruments, China). Band intensity was quantified using NIH ImageJ.

### Luciferase Reporter Assays

Luciferase reporter activity was assessed with a Dual‐Luciferase Reporter Assay System (Promega), which normalizes Firefly luciferase activity under the control of the target gene's promoter to baseline Renilla luciferase activity under the control of a CMV promoter. All Firefly luciferase reporter vectors were custom‐synthesized by GenePharma (Shanghai, China). For all assays, each Firefly luciferase reporter vector (0.25 µg) was co‐transfected with a pGL4.75 Renilla luciferase plasmid (6.25 ng) for 24 h with DharmaFECT Duo transfection reagent (ThermoFisher), followed by incubation at 37 °C for 24 h with human oxLDL (25 µg/ml; 5685‐3557, Bio‐Rad) prior to the reporter assay. To ascertain the functionality of the conserved c‐JUN binding site within the 0.6‐kb human *HM13* promoter segment (−500/+100 bp), the WT c‐JUN binding site sequence was modified to a mutant (MUT) sequence using the Quick‐Change Site‐Directed Mutagenesis Kit (Stratagene). The WT and MUT *HM13* promoter segments were placed upstream of the Firefly luciferase sequence. Cells were lysed using Passive Lysis Buffer (Promega), immediately followed by bioluminescence assays using a GloMax luminometer (Promega).

### Macrophage‐VSMC Transwell Co‐Culture

See Methods (Supporting Information).

### Statistical Analysis

As effect sizes were not known a priori, sample sizes were determined based on previous studies on atherosclerotic foam cell generation.^[^ ^36^, ^40^ ^]^ The sample size (*n*) for each experiment is stated in Figure Legends. Each data point was representative of a single tissue donor or biological replicate. Recipient mice in the *ApoE* ^−/−^ chimeric studies were randomized by a random number generator. All data was analyzed by investigators blinded to group assignment. No animals were excluded from this study. Unless otherwise mentioned, all data have been charted as means ± SDs or medians ± upper/lower quartiles using GraphPad Prism software. For comparisons of two groups, a two‐tailed Student's *t*‐test, Mann–Whitney *U* test is used, or Wilcoxon signed‐rank test as appropriate. To compare three or more groups, analysis of variance (ANOVA) is employed with Tukey's post‐hoc test or the Kruskal–Wallis test as appropriate. *p*‐values of less than 0.05 were considered significant for all analyses.

## Conflict of Interest

The authors declare no conflict of interest.

## Supporting information



Supporting Information

## Data Availability

The data that support the findings of this study are available in the supplementary material of this article.
