# Supplementary material for: Macrophage HM13/SPP Enhances Foamy Macrophage Formation and Atherogenesis
Source: Adv Sci (Weinh). 2025 Mar 20;12(18):2412498. doi: 10.1002/advs.202412498 (PMC12079524; doi:10.1002/advs.202412498)
Supplement: Supplementary file 1 — Supporting Information [file ADVS-12-2412498-s001.docx]

Supporting Information

**Macrophage HM13/SPP Enhances Foamy Macrophage Formation and Atherogenesis**

Contents

[Supporting Methods 3](#_Toc188612438)

[Theoretical Background for Our *In Silico* Approach 3](#_Toc188612439)

[WGCNA 4](#_Toc188612440)

[Differential Co-Expression Analysis 6](#_Toc188612441)

[Evaluation of Module Preservation 6](#_Toc188612442)

[Functional Enrichment Analyses 7](#_Toc188612443)

[Determination of the Module Hub Gene Signature Scores and Macrophage Plaque Gene Signature Score 7](#_Toc188612444)

[Single-Cell RNAseq (scRNAseq) Analysis of Human Atherosclerotic Plaque Data 8](#_Toc188612445)

[Method for Classifying Macrophages in the Human Atherosclerotic Plaque scRNAseq Analysis 8](#_Toc188612446)

[Promoter Analysis 11](#_Toc188612447)

[Collection and Analyses of Human Coronary Artery Specimens 11](#_Toc188612448)

[Collection and Analyses of Human Carotid Plaque Specimens 12](#_Toc188612449)

[Construction and Characterization of Myeloid-Specific Transgenic *Hm13* Murine Models 13](#_Toc188612450)

[Generation and Packaging of Lentiviral Vectors 14](#_Toc188612451)

[Isolation and Transduction of Human Monocyte-Derived Macrophages (hMDMs) and Murine Bone Marrow-Derived Macrophages (mBMDMs) 15](#_Toc188612452)

[Flow Cytometry-Based Sorting of Bone Marrow Cells 16](#_Toc188612453)

[Myeloid Green Fluorescent Protein (GFP) Quantification 16](#_Toc188612454)

[ELISA Studies in mBMDMs 16](#_Toc188612455)

[Transduction and Differentiation of THP-1 Cells 17](#_Toc188612456)

[Pulse-Chase Analyses for HO-1 Cleavage and Degradation 17](#_Toc188612457)

[Macrophage-VSMC Transwell Co-Culture 18](#_Toc188612458)

[Supporting Files 19](#_Toc188612459)

[Supporting File 1 DEGs that Significantly Correlate with *HM13* in Monocyte-Derived Macrophages (MDMs) from 19](#_Toc188612460)

[Supporting Tables 20](#_Toc188612461)

[Supporting Table 1 Clinicodemographic Characteristics of the Human Coronary Artery Donors (n=20). 20](#_Toc188612462)

[Supporting Table 2 Clinicodemographic Characteristics of the Human Carotid Plaque Donors (n=10). 22](#_Toc188612463)

[Supporting Table 3 Primary and Secondary Antibodies Used for Immunohistochemistry. 23](#_Toc188612464)

[Supporting Table 4 qPCR Primer Sequences. 24](#_Toc188612465)

[Supporting Table 5 Primary and Secondary Antibodies Used for Co-Immunoprecipitation (IP) and Immunoblotting (IB). 25](#_Toc188612466)

[Supporting Table 6 Results of the Differential Co-Expression Analysis. 26](#_Toc188612467)

[Supporting Table 7 Results of the Module Preservation Analysis. 27](#_Toc188612468)

[Supporting Figure Legends 28](#_Toc188612469)

[Supporting Figures 35](#_Toc188612470)

[Supporting Figure 1 35](#_Toc188612471)

[Supporting Figure 2 36](#_Toc188612472)

[Supporting Figure 3 37](#_Toc188612473)

[Supporting Figure 4 38](#_Toc188612474)

[Supporting Figure 5 39](#_Toc188612475)

[Supporting Figure 6 40](#_Toc188612476)

[Supporting Figure 7 41](#_Toc188612477)

[Supporting Figure 8 42](#_Toc188612478)

[Supporting Figure 9 43](#_Toc188612479)

[Supporting Figure 10 44](#_Toc188612480)

[Supporting Figure 11 45](#_Toc188612481)

[Supporting Figure 12 46](#_Toc188612482)

[Supporting Figure 13 47](#_Toc188612483)

[Supporting Figure 14 48](#_Toc188612484)

[Supporting Figure 15 49](#_Toc188612485)

[Supporting Figure 16 50](#_Toc188612486)

[Supporting Figure 17 51](#_Toc188612487)

[Supporting Figure 18 52](#_Toc188612488)

[Supporting Figure 19 53](#_Toc188612489)

[Supporting Figure 20 54](#_Toc188612490)

[Supporting Figure 21 55](#_Toc188612491)

[Supporting Figure 22 56](#_Toc188612492)

[Supporting Figure 23 57](#_Toc188612493)

[Supporting Figure 24 58](#_Toc188612494)

[Supporting Figure 25 59](#_Toc188612495)

[Supporting Figure 26 60](#_Toc188612496)

[Supporting References 61](#_Toc188612497)

# Supporting Methods

## Theoretical Background for Our *In Silico* Approach

Gene co-expression correlations provide a robust methodology for predicting gene function, as genes which share a biological process are often co-regulated (several reviews provide systematic evidence for this [[1](#_ENREF_1)]). Indeed, this idea forms the basis of many studies in the field of functional genomics that approach this concept at different levels:

*Global gene expression profiling and clustering analysis.* Studies often use microarray or RNA sequencing technologies to analyse the expression levels of thousands of genes simultaneously. By comparing gene expression profiles across different tissues, conditions, or organisms, researchers can identify patterns that suggest functional similarities. A method to identify such patterns are clustering analysis, in which genes within the same cluster are likely to share similar functions. As an illustrative example, a study by Eisen et al. [[2](#_ENREF_4)] introduced hierarchical clustering to analyse gene expression data, revealing distinct patterns that correlated with known biological functions.

*Functional genomics approaches.* Functional genomics studies often integrate gene expression data with other types of genomic information, such as functional annotations, pathway analyses, and protein-protein interaction networks. The review by Barabási et al. [[3](#_ENREF_5)] discusses the principles of systems biology and how integrating different types of biological data, including gene expression, can uncover functional relationships.

*Evolutionary conservation.* Evolutionary conservation of gene expression patterns across species is often used as evidence for functional relevance. Genes with conserved expression and co-expression patterns are more likely to have conserved functions. This is exemplified in a study by Stuart et al. [[4](#_ENREF_6)], who investigated the conservation of gene expression programs across different species, providing insights into the functional significance of conserved expression.

*Machine learning approaches.* Machine learning techniques, such as support vector machines and random forests, can be applied to predict gene function based on expression patterns. Studies like the one by Troyanskaya et al. [[5](#_ENREF_7)] demonstrated the use of machine learning to predict gene function using gene expression data across diverse organisms and to a certain level provide a validation of co-expression as a method to uncover common functionality.

*Single-cell approaches.* New sets of evidence are being accumulated more recently with the availability of massive datasets of gene expression at the single-cell level. As certain cell types are well defined by their functionality, it is possible to associate specific co-expression networks with particular cell types. Ribeiro et al. [[6](#_ENREF_8)] provide an in-depth view of local gene co-expression and regulatory element co-activity using single-cell data. As an example, using single-cell RNA-seq, Gaublomme et al. [[7](#_ENREF_9)] investigated the heterogeneity within Th17 cells and identified co-expressed gene modules associated with the pathogenicity of these cells, showcasing that co-expressed genes lead to cell type-specific functions.

In conclusion, the literature strongly supports the idea that similar patterns of gene expression are indicative of similar functions. This concept has been demonstrated through various experimental and computational approaches, providing a foundation for understanding the functional roles of genes based on their expression profiles.

## WGCNA

Transcriptomic microarray data from internal mammary arterial (IMA) wall (non-atherosclerotic control) samples and aortic root wall (atherosclerotic) samples of 40 coronary artery disease (CAD) patients from the Stockholm Atherosclerosis Gene Expression (STAGE) cohort was obtained from NCBI’s Gene Expression Omnibus (GEO) data repository portal (GEO acc. no. GSE40231) [[8](#_ENREF_10)]. There are two reasons for selection of the GSE40231 dataset. First, when designing this study, we did consider merging the GSE40231 dataset with other atherosclerosis datasets. However, we were not able to identify any other publicly-available GEO dataset with aortic root wall samples and IMA control samples with which we could reliably merge with the GSE40231 dataset. Second, the GSE40231 is a highly-reputable dataset from a large cohort of atherosclerosis patients and can, therefore, stand alone for purposes of analysis. In our analysis, the aortic root wall has been used as an experimental tissue because the aortic root wall is susceptible to atherosclerosis [[9](#_ENREF_11)]. The IMA wall has been used as a control tissue because the IMA wall is resilient to atherosclerosis [[10](#_ENREF_13)]. This phenomenon has been attributed to (i) the IMA’s anatomy that favors anti-inflammatory, cytoprotective anterograde blood flow and (ii) the biological characteristics of the IMA wall’s endothelial layer and SMCs.

The R package WGCNA was applied to the aforementioned STAGE cohort transcriptomic microarray data to identify gene modules (clusters) associated with the four atherosclerosis-causal RGN driver genes *AIP*, *DRAP1*, *POLR2I*, or *PQBP1* in atherosclerotic plaques. In essence, the WGCNA package clusters genes based on patterns of co-expression and constructs a gene co-expression network [[11](#_ENREF_14)]. Signed networks were constructed using the blockwiseModules function of the WGCNA package. Modules were defined using biweight midcorrelation (bicor), with the top 20% most variable annotated genes (*n*=8933) and a soft-threshold power of eight to achieve approximate scale-free topology (*R*^2^>0.8). “bicor” is a median-based correlation measure that is more robust than the Pearson correlation measure, which is susceptible to outliers [[12](#_ENREF_15)]. We simulated different combinations of hyperparameters (i.e., deep split, minimal module size, merge threshold and pamstage) as previously described [[13](#_ENREF_16)]. The specific tuning parameters were: deepSplit = 0, 2, and 4 (from less to most sensitive); minModuleSize = 50, 100, and 200 (favoring smaller module sizes to increase pathway specificity); pamStage = TRUE or FALSE; and mergeCutHeight = 0.15 and 0.25. This resulted in 36 different combinations of parameters. Based on the iteration results, the final network was built with a deepSplit of 2 (for higher sensitivity) and a minimum module size of 100 (to avoid capturing potential noise with the module size of 50). PamStage was kept as FALSE and mergeCutHeight at 0.25. The resulting ten modules were found in most hyperparameter combinations, with module membership ranging from 155 to 1870 genes. The grey module, grouping non-significant genes, consisted of 2404 features. To produce visual representations of modular networks, our final WGCNA network was re-built as an object suitable for the BioNERO R package. Next, edge lists were extracted for each of the modules of interest based on their adjacency matrices, and networks were plotted using the ‘plot_gcn’ function. The module eigengene (ME) was calculated based on the first principal component of each module. Each module’s first principle component is used to calculate the module’s eigengene (ME). ME values were Pearson correlated with sample traits defined by the four atherosclerosis-causal RGN driver genes *AIP*, *DRAP1*, *POLR2I*, or *PQBP1*.

## Differential Co-Expression Analysis

As previously described [[14](#_ENREF_17)], we performed a differential co-expression analysis between the WGCNA modules for the two conditions of the study. To this end, we calculated the modular differential connectivity (MDC) for each module comparing the aortic root wall (atherosclerotic) samples to the IMA wall (non-atherosclerotic control) samples using the R DCGA package [[15](#_ENREF_18)]. This analysis finds the average change in correlation between gene symbols in the two conditions and the significance of that change in correlation, as well as the top genes with a gain/loss in correlation with the other genes in the module between the conditions (if any of them are significant). We used the same input matrix and resulting modules of the final network construction, with 100 permutations to calculate the significance of the result.

## Evaluation of Module Preservation

The STAGE and STARNET groups have already built modules for the STAGE data used in the present study. In particular, such modules have emphasized their robustness across different tissues in the context of CAD [[8](#_ENREF_10), [16](#_ENREF_19)]. To compare our modules with those published, we used the NetRep R package [[17](#_ENREF_20)] that provides specific functions for comparing gene co-expression networks. To this end, we followed a similar protocol as those published to build independent co-expression networks using WGCNA for each additional tissue type in the STAGE dataset (i.e., liver, skeletal muscle, and visceral fat). Next, we assessed the preservation of our own modules (based only on arterial tissues) within each of those tissue-specific networks. NetRep calculates seven module preservation statistics for each module, and performs a permutation procedure in the test dataset to determine whether these statistics are significant (we used 1000 permutations to establish statistical significance in each comparison). Preservation statistics were interpreted as a whole instead of choosing a single metrics to define significance. The seven module preservation statistics for each comparison are defined as follows:

‘cor.cor’ measures the concordance of the correlation structure (how similar the correlation heatmaps are between the two datasets);

‘avg.cor’ measures the average magnitude of the correlation coefficients of the module in the test dataset (how tightly correlated the module is on average in the test dataset. This score is penalised where the correlation coefficients change in sign between the two datasets);

‘avg.weight’ measures the average magnitude of edge weights in the test dataset (how connected nodes in the module are to each other on average);

‘cor.degree’ measures the concordance of the weighted degree of nodes between the two datasets (whether the nodes that are most strongly connected in the discovery dataset remain the most strongly connected in the test dataset);

‘cor.contrib’ measures the concordance of the node contribution between the two datasets (this measures whether the module’s summary profile summarises the data in the same way in both datasets);

‘avg.contrib’ measures the average magnitude of the node contribution in the test dataset (a measure of how coherent the data is in the test dataset. This score is penalised where the node contribution changes in sign between the two datasets: for example, where a gene is differentially expressed between the two datasets); and ‘coherence’ measures the proportion of variance in the module data explained by the module’s summary profile vector in the test dataset.

## Functional Enrichment Analyses

Among the four atherosclerosis-causal RGN driver genes, the modular pattern associated with *AIP* displayed the strongest resemblance to the modular pattern for the atherosclerotic trait. Therefore, the modules most positively and negatively correlating with *AIP* were chosen for functional enrichment analyses using the Cluster Profile package in R. The following databases were interrogated: Gene Ontology Biological process (GO-BP), BioCarta, Kyoto Encyclopaedia of Genes and Genomes (KEGG), and Reactome pathway.

## Determination of the Module Hub Gene Signature Scores and Macrophage Plaque Gene Signature Score

The top 120 hub genes from each module were used to generate each module’s Hub Gene Signature Score by summarizing the Z-normalized log_2_RSEM (RNA-Seq by Expectation-Maximization) from an independent left anterior descending (LAD) coronary atherosclerotic plaque gene expression dataset (GEO acc. no.: GSE11138) [[18](#_ENREF_21)]. The Macrophage Plaque Gene Signature Score – based on a curated 15-gene macrophage plaque gene signature (i.e., *ZMYND15* (*DKFZp434N127*), *DISC1*, *KIF21B* (*KIAA0449*), *RUNX1*, *LILRB3*, *SYK*, *CPVL*, *LY86* (*MD1*), *DENND1A* (*KIAA1608*), *FAM78A* (*FLJ00024*), *FAM20A* (*DKFZp434F2322*), *NOD2* (*CARD15*), *ADRBK2*, *CORO7* (*FLJ22021*), and *WDFY4* (*KIAA1607*) from a previous study [[19](#_ENREF_22)] -- was calculated by summarizing the Z-normalized log_2_RSEM of the expression data for the 15 macrophage plaque signature genes in the GSE11138 gene expression dataset. Signature scores were calculated using the ‘singscore’ package that is based on a ranking system and therefore more robust to differences between samples and datasets.

## Single-Cell RNAseq (scRNAseq) Analysis of Human Atherosclerotic Plaque Data

Human atherosclerotic carotid plaque scRNAseq data (10xGenomics), developed by Fernandez et al. [[20](#_ENREF_23)], was downloaded from GEO (GSE224273). All analyses were performed using R version 4.2.2 in a Linux environment. Barcode matrices corresponding to six independent carotid artery plaques were loaded into R to create a Seurat object. After filtering and doublet removal with ‘DoubletFinder’, the six Seurat objects were SCT-normalized, integrated to remove batch effects, and clustered using the corresponding functions from the ‘Seurat’ package. Major immune cell types were identified with sctype and validated after calculating a score for each cell type with ‘AUCell’. Macrophages were further subsetted, re-clustered, and re-identified with ‘ScType’ using known subset markers (see detailed explanation in next subsection). The foamy macrophage cluster was further validated using ‘ProjecTILs’ after label transfer from a large scRNAseq dataset of human macrophages [[21](#_ENREF_24)]. Due to known problem caused by sparse data and over-dispersion of counts, gene-gene correlations were performed after data smoothing using ‘Rmagic’. Most visualizations were based on ggplot2.

## Method for Classifying Macrophages in the Human Atherosclerotic Plaque scRNAseq Analysis

Categories were assigned using ‘ScType’, which annotates each cell using a database of markers [[22](#_ENREF_25)]. In the initial step, we used ScType in combination with their “Immune System” database that contains all the major immune cell types. This included the two categories, “Classical Monocytes” and “Macrophages”. “Classical Monocytes” and “Macrophages” clustered very close to each other and shared many of the markers in the database. So, we selected both of them for renormalization and re-clustering. As depicted in the left panel below, the two clusters were still clearly separated from each other after re-clustering. However, the unsupervised clustering resulted in four different categories (see middle panel below).


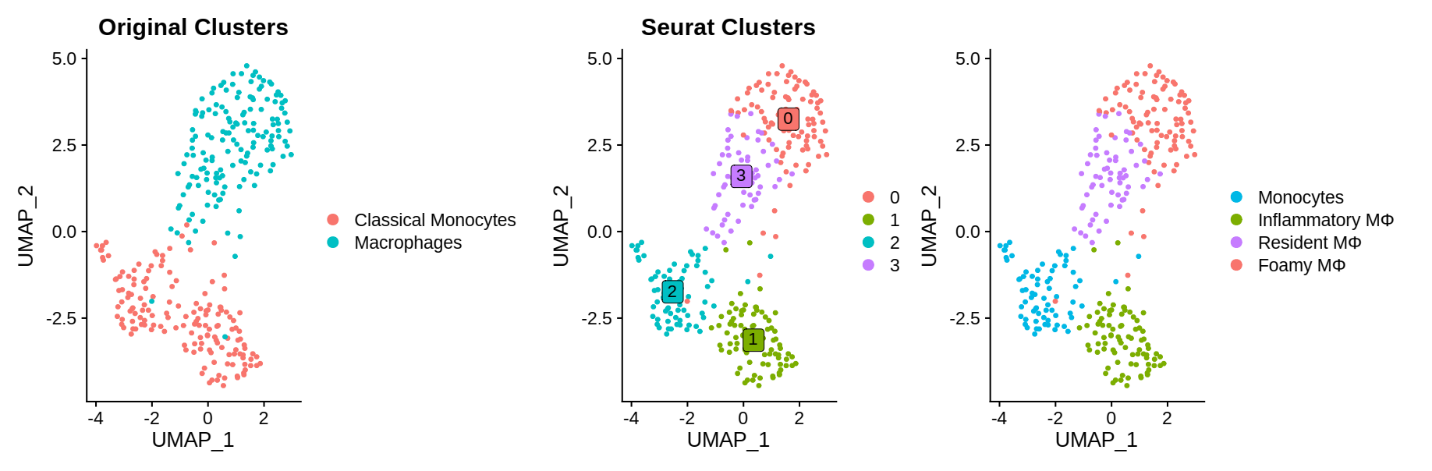
Each of the two original clusters (“Classical Monocytes” and “Macrophages”) consisted of two subcategories in the Seurat clustering. We then repeated the ScType analysis with a more refined set of markers specific for macrophages in atherosclerotic plaques as follows [[23](#_ENREF_26)]:

• Resident macrophages: *CBR2, CD206, CX3CR1, F13A1, FOLR2, GAS6, LYVE1, MRC1, PF4,* and *SEPP1*.

• Inflammatory macrophages: *CEPBP, CXCL2, EGR1, IER3, IL1B, NKFBIA, NLRP3, TLR2, TNF,* and *ZPF36*.

• Foamy macrophages: *CD9, CTSB, FABP5, LGALS3, OPN (SPP1),* and *TREM2*.

This resulted in the four categories shown in the right panel above. To verify this, we calculated a score based on the same genes using ‘AUCell’ and visualized its density, as shown below:


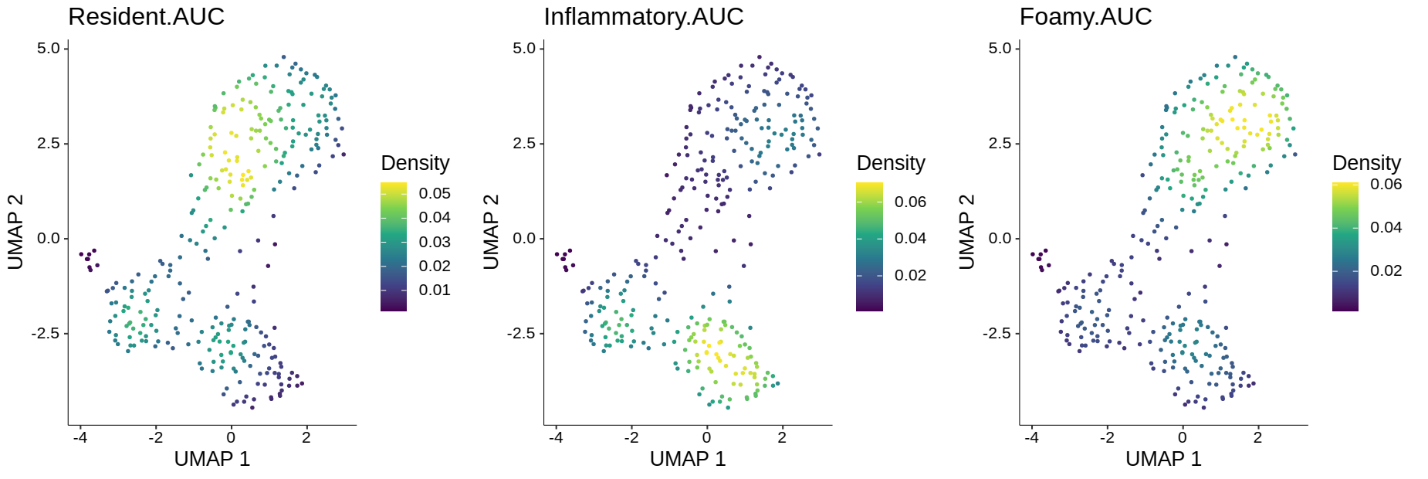


The density scores map reasonably well to the aforedescribed Seurat clusters.

For classification along the M1 (inflammatory)-M2 (anti-inflammatory) macrophage polarization spectrum [[24](#_ENREF_27)], a score for each macrophage polarization category and each cell was calculated using the area under the curve (AUC) method. The scores were based on published markers for each polarized subtype [[24-25](#_ENREF_27)] as follows (note: some markers are present in more than one polarized subtype):

• M1: *TNF, IL6, IL12A, NOS1, NOS2, HLA-DRA, CXCL2, CCL3, CCL4, CEBPB, CD80, CD86, CXCL10, CXCL9, CXCL11, GBP1, STAT1, IFNG, PSMB8,* and *CD2*

• M4: *MMP7, MMP12, S100A8, S100A9,* and *CCL2*

• Mox: *HMOX1, NRF1, SRXN1, NFE2L2,* and *GLRX5*

• Mhem: *CD163, HMOX1, MSR1, NR1H3, NR1H2, ABCA1, ABCG1 HBA1 HBA2 HBB HBD CYP27A1 SLC40A1,* and *FTH1*

• MHb: *HPR, CD163, HPX, MRC1, CD163L1, NR1H3, ABCA1,* and *ABCG1*

• M2: *IL10, TGFB1, ARG1, MRC1, CHI3L1, CD163, CD206, PDGFB, MSR1, PPARG, RETNLB, LYVE1*

*FOLR2,* and*F13A1*

The AUC scores corresponding to each of the final four clusters are shown below:


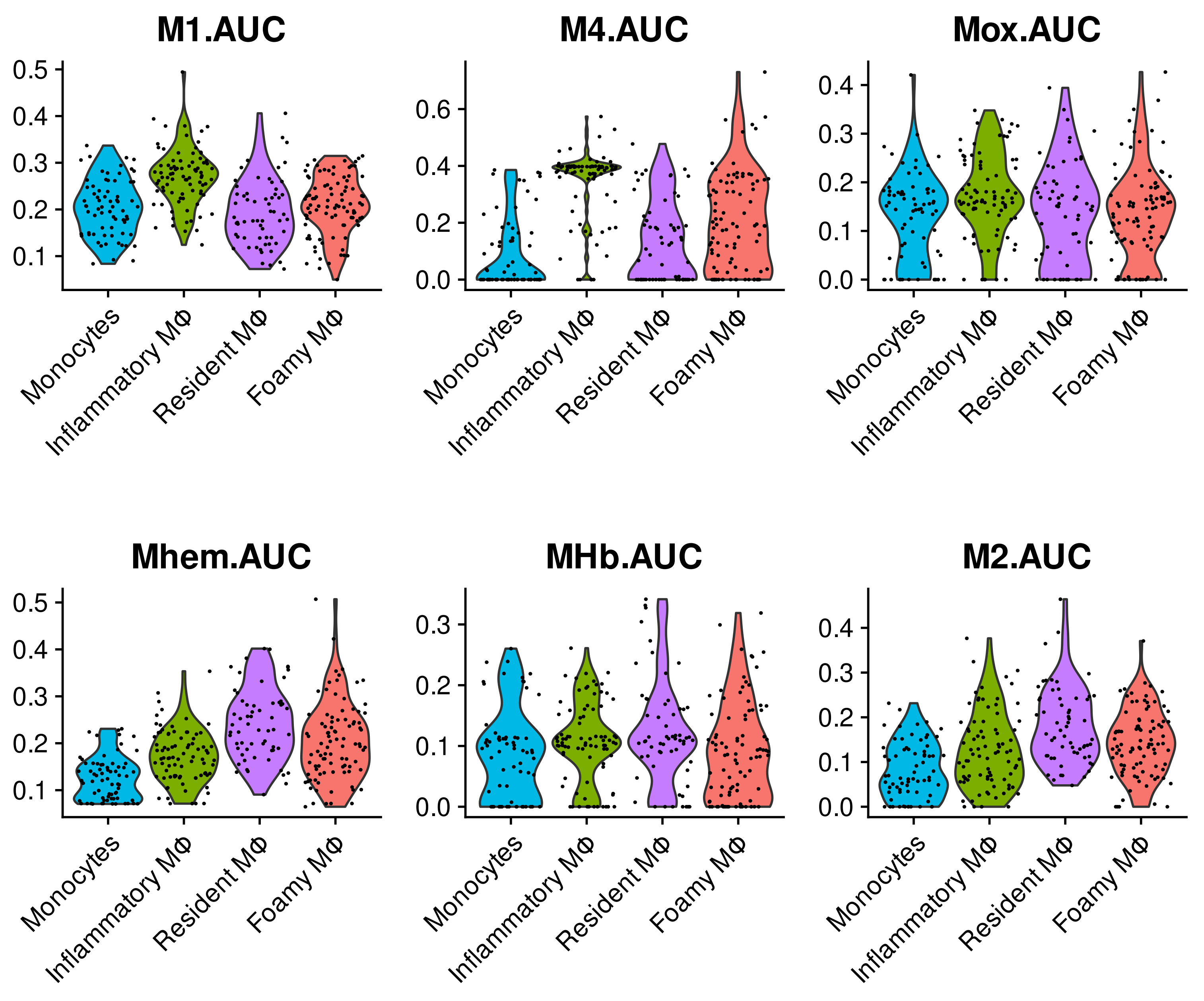


Monocytes scored low-to-intermediate across all polarization phenotypes. Inflammatory macrophages scored high for M1 and M4, while resident macrophages scored high for M2 and Mhem. Foamy macrophages scored intermediate across all polarization phenotypes.

## Promoter Analysis

Putative AHR/ARNT, PPARG, and c-JUN binding sites on the human *HM13* gene promoter (*HM13* chr20:30102240, number of introns: 2, NM_178582; core promoter region=1000 bp) were localized using the ConTra V3 tool [[26](#_ENREF_31)].

**Selection of the Dysfunctional Foamy Macrophage Markers FABP5 and OPN (SPP1)**

The identification and characterization of foamy macrophages within atherosclerotic lesions is a rapidly-developing field of study. Several foamy macrophage-specific marker genes, including *CD9*, *FABP5*, *LGALS3*, *OPN* (*SPP1*), and *TREM2*, have been identified in scRNA-seq studies on atherosclerotic plaques (extensively reviewed here [[23](#_ENREF_26)]). However, recent work differentiates between functional versus dysfunctional foamy macrophage subpopulations in plaques [[27](#_ENREF_32)]. Therefore, not all recognized foamy macrophage-specific markers correspond to dysfunctional foamy macrophage subpopulations that contribute to atherosclerosis. For example, TREM2 has been found to reduce necrotic core formation and improve plaque stability [[28](#_ENREF_33)]. In this study, FABP5 and OPN (SPP1) were selected as dysfunctional foamy macrophage-specific markers for studies on the human atherosclerotic plaque samples due to (i) their positive relationships with atherosclerotic development [[27](#_ENREF_32), [29](#_ENREF_35)] and (ii) strong association with the foamy macrophage cluster in our human atherosclerotic carotid plaque scRNAseq analysis (see heatmap in Supporting Figure S10b).

## Collection and Analyses of Human Coronary Artery Specimens

Human coronary artery specimens were obtained from twenty cadavers following fatal acute ST-elevation myocardial infarction (STEMI). Inclusion criteria were: (i) the cadaver was refrigerated for less than 48 hours and was dissected within five days of death; and (ii) the presence of atherosclerotic coronary vessels (plaque burden>25%) and matching normal coronary vessels was confirmed by integrated backscatter intravascular ultrasound (IB-IVUS) [[30](#_ENREF_37)]. Exclusion criteria were: (ii) presence of malignancy, cachexia, end-stage renal disease, renal failure, multiple organ dysfunction, active infection, sepsis, autoimmune disease, or other systemic inflammatory conditions at the time of death; or (ii) autolysis or corruption of coronary arterial tissue. For each heart, two coronary plaque samples (plaque burden>25%) and one normal coronary sample were preserved at −80 °C for fluorescence-activated cell sorting (FACS) and qPCR analyses.

Coronary samples were finely minced and suspended in a 0.025 mg/mL Liberase TM solution (Roche), which contained collagenase I and II along with 40 µg/mL DNase (Stemcell Technologies) in pure Dulbecco's modified Eagle's medium (DMEM, Sigma). This mixture was incubated at 37°C for 45 minutes to facilitate enzymatic digestion. To enhance tissue dissociation, magnetic beads (VWR) and a magnetic stirrer were used for mechanical fragmentation. After digestion, the tissue was filtered through a 70-μm cell strainer, followed by low-speed centrifugation at 50 g for 2 minutes to remove cardiomyocytes, cell aggregates, and undigested tissue. The resulting supernatant was then passed through a 40-μm cell strainer, centrifuged at 450 g for 4 minutes, and washed with PBS. Finally, the cell pellet was resuspended in FACS buffer consisting of PBS with 1% (v/v) ultralow endotoxin fetal bovine serum (FBS; Hyclone), and 1 mM EDTA (Invitrogen).

For FACS, the cell suspension was pre-incubated with normal rat serum and an anti-CD16/32 antibody for 15 min on ice prior to antibody staining. Cells were incubated on ice for 30 minutes with a mix of fluorochrome-conjugated antibodies diluted in FACS buffer as follows: CD45-Phycoerythrin (PE) (1:300, clone 30-F11, Invitrogen), CD68-Allophycocyanin (APC) (1:300, clone 298807, Invitrogen), FABP5-Alexa Fluor 350 (AF350) (1:300, cat. no. FAB3077U, Novus Bio), and OPN-Fluorescein isothiocyanate (FITC) (1:300, cat. no. NBP2-89443F, Novus Bio). To differentiate live cells from dead cells, 4′,6-diamidino-2-phenylindole (DAPI, 1 μg/mL, Roche) was included in the staining protocol. All cells were analysed and sorted using a LSRFortessa flow cytometry instrument (BD Biosciences) and FlowJO software. After sorting, select cell populations were subjected to quantitative reverse transcription PCR (qPCR) as described in the Methods.

## Collection and Analyses of Human Carotid Plaque Specimens

Human atheromatous carotid plaque specimens were obtained from ten patients that underwent symptomatic carotid endarterectomy (CEA). Inclusion criteria were: (i) plaque presence within the internal carotid artery (ICA), common carotid artery (CCA), or carotid artery bifurcation; and (ii) stroke or transient ischemic attack (TIA) within six months prior to CEA. Exclusion criteria were: (ii) history of previous CEA, carotid stenting, carotid aneurysm, carotid kinking, malignancy, cachexia, end-stage renal disease, renal failure, multiple organ dysfunction, active infection, sepsis, autoimmune disease, or other systemic inflammatory conditions at the time of CEA. Carotid plaque specimens were preserved at −80 °C. Two specimens were randomly selected for immunofluorescent staining. Each of the remaining eight specimens were divided into three parts; these 24 plaque samples were used for *ex vivo* foamy macrophage culture and ELISA as described below.

Immunofluorescent staining was performed as previously described [[31](#_ENREF_38)]. Briefly, human carotid plaque sections were xylene-dewaxed and dehydrated by an ethanol gradient. Heat-induced epitope retrieval was followed by blocking with 5% BSA in TBS for 20 min. The sections were treated with primary antibodies for 120 min, treated with fluorescent secondary antibodies for 60 min in a darkroom, and then mounted with DAKO mounting medium (Agilent). The details regarding the primary and secondary antibodies are available in Supporting Table 3. Images were captured with a Ti Eclipse inverted fluorescence microscope (Nikon).

Foamy macrophages were isolated from human carotid plaque specimens by enzymatic digestion and discontinuous density gradient centrifugation and cultured *ex vivo* as previously described [[32](#_ENREF_39)]. After 24 hours, inverted microscopy (Nikon) was used to confirm the predominant presence of lipid-laden foamy macrophages (>95%). ELISA was performed using commercial kits for AIP (cat. no. abx384602), HM13/SPP (cat. no. abx527185), and HO-1 (cat. no. abx252635) (Abbexa).

## Construction and Characterization of Myeloid-Specific Transgenic *Hm13* Murine Models

All mice were housed in a controlled environment in individually-ventilated cages at 22°C. They were fed a standard chow diet (Keao Xieli Feed, Beijing, China) and filtered water *ad libitum.* Floxed-*Hm13* mice (*Hm13*^fl/fl^) on a C57BL/6J background (Shanghai Model Organisms) were mated with *Lyz2-2A*^Cre^ mice (Shanghai Model Organisms) to generate *Hm13*^mKO^ mice. For *Hm13* overexpression, murine *Hm13* cDNA (NM_001159553) was introduced into the ribosomal entry site pROSA26, which is loxP-flanked STOP and Frt-flanked eGFP targeting construct [[33](#_ENREF_40)]. This construct was integrated into ubiquitously-expressed *Rosa26* locus of BRUCE4 mouse ES cells of C57BL/6J origin. Southern blot was used to confirm the insertion. *Lyz2-2A*^Cre^ mice were crossed with Rosa26.*Hm13* mice to generate *Hm13*^mOE^ mice. A similar procedure was employed to generate *Hm13*^mOE^;*Hmox1*^mOE^ mice with the dual use of murine *Hm13* cDNA and murine *Hmox1* cDNA (NM_010442.2). *ApoE*^−/−^ mice were crossed with the aforedescribed transgenic mice strains (i.e., *Hm13*^fl/fl^, *Hm13*^mKO^, *Hm13*^mOE^, and *Hm13*^mOE^;*Hmox1*^mOE^) to generate the respective F1 *ApoE*^+/−^ transgenic mouse strains. Then, these F1 progeny were crossed with *ApoE*^−/−^ mice to produce the final *ApoE*^−/−^ transgenic mouse strains (i.e., *ApoE*^−/−^*Hm13*^fl/fl^, *ApoE*^−/−^*Hm13*^mKO^, *ApoE*^−/−^*Hm13*^mOE^, and *ApoE*^−/−^*Hm13*^mOE^;*Hmox1*^mOE^). PCR-based amplification of ear-clip samples was used to genotype offspring.

Blood obtained via cardiac puncture was heparinized, and blood counts were obtained on a XN-1000 hematology analyzer (Sysmex, Kobe, Japan). Sections of paraffin-embedded adipose tissue and liver samples were stained with hematoxylin and eosin (H&E) or were incubated with an anti-F4/80 antibody (1:500; sc-52664, SCBT) followed by incubation with biotinylated rabbit anti-rat antibody (1:200; ab6733, Abcam) and PE-streptavidin (Bio-Rad). These samples were mounted with Diamond Antifade Mountant with 4',6-diamidino-2-phenylindole (DAPI, Molecular Probes). Fields were captured with an Eclipse E800 microscope equipped with a DS-U1 camera and NIS Elements v. 2.34 (Nikon). Mean size of adipocytes were calculated based on 10 cells per field of view and five fields of view per mouse.

## Generation and Packaging of Lentiviral Vectors

All lentiviral vectors described in this study were constructed from pcDNA5 parent plasmids by GenePharma (Shanghai, China). Briefly, cDNA sequences were extracted from the pcDNA5 parent plasmids, and each cDNA sequence was individually inserted into a pCDH-CMV-mCherry-T2A-Puro lentiviral plasmid (Addgene plasmid #72264). After validating cDNA directionality and sequence fidelity via sequencing, HEK293T cells were co-transfected with each pCDH-CMV plasmid and the packaging vectors pMD2.G (Addgene plasmid #12259) and psPAX2 (Addgene plasmid #12260) using Lipofectamine 2000. After 48 h, lentivirus-containing HEK293T supernatants were filtered using 0.45-µm Millex-HV syringe filters and stored at -80°C.

## Isolation and Transduction of Human Monocyte-Derived Macrophages (hMDMs) and Murine Bone Marrow-Derived Macrophages (mBMDMs)

Whole blood samples were collected from 20 healthy adult volunteers (age (mean±SD): 54.7±2.9). Human CD14^+^ monocytes were isolated from whole blood samples by Ficoll-Paque PLUS (17144003, GE Healthcare) coupled density centrifugation followed by magnetic selection with CD14 Human MicroBeads (Miltenyi Biotec). For hMDM generation, these CD14^+^ monocytes were differentiated for seven days in RPMI-1640 media (Gibco), MCS-F (100 ng/ml) (PeproTech), 10% (v/v) ultralow endotoxin fetal bovine serum (FBS; Hyclone), 1 mM glutamine (Invitrogen), and 1% penicillin/streptomycin (Gibco). For mBMDM generation, bone marrow (tibiae/femurs) cells isolated from transgenic mice (male and female, 12-13 weeks old) were differentiated for five days in DMEM with 10% (v/v) L929-conditioned medium, 10% (v/v) ultralow endotoxin FBS, and penicillin (100 U/ml)/streptomycin (100 ng/ml).

mBMDMs were transduced with lentiviral vectors bearing FLAG-tagged murine wild-type (WT) *Aip* cDNA (NM_016666), FLAG-tagged murine *Aip* carboxy-terminus deletion mutant (AipΔCT) cDNA [[34](#_ENREF_41)], or murine WT *Hm13* cDNA (NM_010376) with a V5 tag. hMDMs were transduced with lentivirus-containing HEK293T supernatants bearing FLAG-tagged human WT *AIP* cDNA (NM_003977), FLAG-tagged human mutant *AIP* (AIPΔCT) cDNA [[34](#_ENREF_41)], constitutively-active human p38α^D176A/F327S^ cDNA with an HA tag [[35](#_ENREF_42)], or constitutively-active human c-JUN^S63/73D^ cDNA with a MYC tag [[36](#_ENREF_43)]. Empty lentiviral vectors were used as negative controls. Briefly, target macrophages (2 × 10^5^) were transduced with lentivirus-containing HEK293T supernatants for 12 h in the presence of 1 µg/ml polybrene. The medium was replaced and, 24 h later, transduced macrophages were selected with 2 μg/ml puromycin over four days. Following fluoroscopic confirmation of mCherry pCDH-CMV plasmid marker expression, macrophages were incubated at 37°C for 24 h with human nLDL (25 µg/ml; 5685-3204, Bio-Rad), human oxLDL (25 µg/ml; 5685-3557, Bio-Rad), or dimethyl sulfoxide (DMSO) vehicle control (Calbiochem) and subjected to experimentation.

## Flow Cytometry-Based Sorting of Bone Marrow Cells

Sorting of bone marrow cells by flow cytometry was performed as previously described [[37](#_ENREF_44)]. Briefly, bone marrow cells were pre-incubated with normal rat serum and an anti-CD16/32 antibody for 15 min on ice prior to antibody staining. Cells were incubated on ice for 30 minutes with a carefully selected mix of fluorochrome-conjugated antibodies diluted in FACS buffer. Eosinophils were sorted from bone marrow cells as FSC^lo^SSC^hi^Siglec-F^+^ cells. Neutrophils were sorted from bone marrow cells as Gr-1^hi^ cells. Basophils were isolated from bone marrow cells with a biotinylated anti-CD49b antibody/streptavidin-conjugated magnetic particle system (BD Pharmingen) followed by sorting for FSC^lo^SSC^lo^CD200R3^+^c-kit^−^ cells. To differentiate live cells from dead cells, 4′,6-diamidino-2-phenylindole (DAPI, 1 μg/mL, Roche) was included in the staining protocol. All cells were analysed and sorted using a LSRFortessa flow cytometry instrument (BD Biosciences) and FlowJO software.

## Myeloid Green Fluorescent Protein (GFP) Quantification

Percentages of GFP^+^ cells within the monocytic myeloid lineage (Cd11b^+^/Ly6c^+^) compartment [[38](#_ENREF_45)] of bone marrow (tibiae/femurs) and blood samples were determined as follows. Red blood cells (RBCs) were lysed using a RBC lysis buffer (BD Biosciences). A total of 10^6^ cells per sample were resuspended in PBS, and the dead scells were stained with NIR Zombie Fixable Viability Kit (Biolegend). The following surface-specific antibodies were used to stain the live cells at a concentration of 0.1 µg/ml in 100 µl total volume: phycoerythrin (PE)-conjugated anti-mouse Ly6c (560592, BD Biosciences) and Alexa Fluor 647-conjugated anti-mouse Cd11b (557686, BD Biosciences). The cells were sorted and analysed using a LSRFortessa flow cytometry instrument (BD Biosciences) and FlowJO software.

## ELISA Studies in mBMDMs

Prior to ELISA, mBMDMs were incubated at 37°C for 24 h with human oxLDL (25 µg/ml; 5685-3557, Bio-Rad). For the inflammatory cytokine secretion studies, murine IL-6, MCP-1, and TNF-α levels in the mBMDM culture supernatants were assessed with Quantikine ELISA kits (R&D Systems). For the membrane fraction studies, mBMDMs were subjected to membrane fractionation using the ProteoExtract Subcellular Proteome Extraction Kit. Membrane levels of murine ABCA1, ABCG1, SR-AI, and Na^+^/K^+^-ATPase α1 were determined by ELISA (LSBio).

## Transduction and Differentiation of THP-1 Cells

Mycoplasma-free THP-1 monocytes (TIB-202, ATCC) were cultured in DMEM with 10% FBS at 37°C in 5% CO_2_ incubator. THP-1 monocytes were transduced with lentiviral vectors (i.e., pCDH-CMV/HA-SPP, pCDH-CMV/HA-SPP^S265A^, pCDH-CMV/FLAG-HMOX1, pCDH-CMV/MYC-RNF139-RFM, and pCDH-CMV/V5-DERLIN1^G180V^). Empty lentiviral vectors were used as negative controls. Briefly, THP-1 monocytes (2 × 10^5^) were transduced with lentivirus-containing HEK293T supernatants for 12 h in the presence of 1 µg/ml polybrene. The medium was replaced and, 24 h later, transduced THP-1 monocytes were selected with 2 μg/ml puromycin over four days. To transiently co-express SPP-HA^S265A^ in pCDH-CMV/FLAG-HMOX1 cells, a plasmid bearing HA-HM13/SPP^S265A^ (300 ng/well) or empty control plasmid (300 ng/well) was applied to cells in a six-well plate as previously described [[39](#_ENREF_46)]. THP-1 monocytes were differentiated into THP-1 macrophages with 150 nM phorbol 12-myristate 13-acetate (PMA, Sigma Aldrich) over 48 h. Then, expression of the mCherry pCDH-CMV plasmid marker was fluoroscopically confirmed in differentiated THP-1 macrophages. Unless otherwise noted, THP-1 macrophages were harvested 24 h after differentiation for experimentation.

## Pulse-Chase Analyses for HO-1 Cleavage and Degradation

As previously described by Burr et al. [[40](#_ENREF_47)], THP-1 cells were incubated in a medium lacking methionine (Met) and cysteine (Cys) for 30 minutes to induce starvation. They were then labelled with [35S]methionine/cysteine (PerkinElmer) for 10 minutes at 37°C. The [35S]methionine/cysteine labelling was chased by adding complete medium for the specified durations. Cells were washed with PBS and lysed using a solution of 1% NP-40 in TBS, supplemented with protease inhibitors. The postnuclear lysates were separated into membrane and cytosolic fractions and were cleared using IgG-Sepharose beads before immunoprecipitation with specific antibodies and protein A–Sepharose beads. The samples were then washed with 0.1% NP-40 in TBS to remove non-specific interactions, separated from the beads, and analyzed by SDS-PAGE. The resulting radioactive proteins were detected using a Storm scanner (GE Healthcare), and the data was processed using ImageQuant TL software (GE Healthcare).

## Macrophage-VSMC Transwell Co-Culture

Murine aortic vascular smooth muscle cells (VSMCs) were extracted from wild-type C57BL/6J mice (male and female, 12-13 weeks old; Shanghai Model Organisms) and verified as an uncontaminated primary VSMC cell line as previously described [[41](#_ENREF_48)]. VSMCs were cultured in DMEM containing 10% FBS and 1% penicillin/streptomycin prior to co-culture [[41](#_ENREF_48)].

VSMCs and mBMDMs were co-cultured in Transwell chambers containing serum-free RPMI medium (4 mL) and separated by a membrane filter to prevent direct cell-to-cell contact [[42](#_ENREF_49)]. Briefly, VSMCs (5 × 10⁴ cells per well) were cultured to confluence on filter inserts with 0.4-μm pores (Falcon). The VSMC-containing inserts were positioned in the upper portion of the Transwell chamber, with mBMDMs in the lower portion of the Transwell chamber. VSMC-only control samples consisted of VSMCs cultured under the identical conditions with no mBMDMs. This co-culture system was maintained for 72 hours, after which the VSMCs were collected for qPCR, Western blotting, and MMP-9 activity analyses (Mouse MMP-9 Activity Assay, QuickZyme).

# Supporting Files

Supporting File 1 DEGs that Significantly Correlate with *HM13* in Monocyte-Derived Macrophages (MDMs) from **Coronary Artery Disease (CAD)** Patients. *In silico* analyses were performed on microarray transcriptomic data of CD14^+^ monocytes and MDMs isolated from CAD patients (*n*=18), which identified a total of 7971 differentially-expressed genes (DEGs) between MDMs and CD14^+^ monocytes. This file lists the DEGs (*n*=835/7971, 10.5%) that significantly correlated with *HM13* (FDR<0.05).

# Supporting Tables

## Supporting Table 1 Clinicodemographic Characteristics of the Human Coronary Artery Donors (n=20).

| **General characteristics** |  |  |  |  |  |
| --- | --- | --- | --- | --- | --- |
| Age (mean, SD) | 77.6 | ± 8.1 | |  |  |
| Male (n, %) | 15 | 75% | |  |  |
| Creatinine (median, range) (µM) | 69 | (40-134) | |  |  |
| BMI (mean, SD) | 26.1 | ± 5.2 | |  |  |
| **Culprit vessel** |  |  | | |  |
| LAD (n, %) | 10 | 50% |  |  |  |
| RCA (n, %) | 3 | 15% |  |  |  |
| Circumflex (n, %) | 7 | 35% |  |  |  |
| **Comorbidities** |  |  |  |  |  |
| History of CAD (n, %) | 5 | 25% |  |  |  |
| Hypercholesterolemia (all on statin) (n, %) | 7 | 35% |  |  |  |
| Hypertension (n, %) |  |  |  |  |  |
| -Diuretic | 3 | 15% |  |  |  |
| -CCB | 2 | 10% |  |  |  |
| -β-blocker | 1 | 5% |  |  |  |
| -ACEi | 1 | 5% |  |  |  |
| Diabetes (n, %) |  |  |  |  |  |
| -Oral meds | 1 | 5% |  |  |  |
| -Insulin | 0 | 0% |  |  |  |
| Smoking |  |  |  |  |  |
| -Current | 6 | 30% |  |  |  |
| -Former | 7 | 35% |  |  |  |
| Lung disease (n, %) | 2 | 10% |  |  |  |
| Peripheral vascular disease (n, %) | 0 | 0% |  |  |  |
| Cerebrovascular accident (n, %) | 0 | 0% |  |  |  |
| Valvular heart disease (n, %) | 0 | 0% |  |  |  |
| Heart failure (n, %) | 0 | 0% |  |  |  |
| CKD (n, %) | 0 | 0% |  |  |  |
| **Serum markers** |  |  |  |  |  |
| Total cholesterol (mean, SD) (mg/dL) | 208 | ± 17 | | | |
| LDL cholesterol (mean, SD) (mg/dL) | 139 | ± 21 | | | |
| HDL cholesterol (mean, SD) (mg/dL) | 38 | ± 9 | | | |
| Triglycerides (mean, SD) (mg/dL) | 154 | ± 66 | | | |
| C-reactive protein (median, range) (mg/dL) | 0.34 | (0.04-6.11) | | | |
| Serum amyloid A (median, range) (mg/dL) | 0.38 | (0.03-19.86) | | | |

**Abbreviations:** SD, standard deviation; BMI, body mass index; LAD, left anterior descending artery; RCA, right coronary artery; CAD, coronary artery disease; CCB, calcium-channel blocker; ACEi, ACE inhibitor; CKD, chronic kidney disease; LDL, low-density lipoprotein; HDL, high-density lipoprotein.

## Supporting Table 2 Clinicodemographic Characteristics of the Human Carotid Plaque Donors (n=10).

| **General characteristics** |  |  |  |
| --- | --- | --- | --- |
| Age (mean, SD) | 67.8 | ± 11.0 |  |
| Male (n, %) | 7 | 70% |  |
| BMI (mean, SD) | 26.6 | ± 6.5 |  |
| **Presentation** |  |  |  |
| Stroke | 5 | 50% |  |
| Hemispherical TIA | 4 | 40% |  |
| Retinal TIA | 1 | 10% |  |
| **Comorbidities** |  |  |  |
| CAD (n, %) | 2 | 20% |  |
| Hypercholesterolemia (all on statin) (n, %) | 5 | 50% |  |
| Hypertension (n, %) |  |  |  |
| -Diuretic | 3 | 30% |  |
| -CCB | 3 | 30% |  |
| -β-blocker | 1 | 10% |  |
| -ACEi | 1 | 10% |  |
| Diabetes (n, %) |  |  |  |
| -Oral meds | 1 | 10% |  |
| -Insulin | 0 | 0% |  |
| Smoking |  |  |  |
| -Current | 3 | 30% |  |
| -Former | 4 | 40% |  |
| Lung disease (n, %) | 1 | 10% |  |
| Peripheral vascular disease (n, %) | 0 | 0% |  |
| History of previous stroke/TIA (n, %) | 0 | 0% |  |
| Valvular heart disease (n, %) | 0 | 0% |  |
| Heart failure (n, %) | 0 | 0% |  |
| CKD (n, %) | 0 | 0% |  |
| **Serum markers** |  |  |  |
| Total cholesterol (mean, SD) (mg/dL) | 206.4 | ± 19.4 |  |
| LDL cholesterol (mean, SD) (mg/dL) | 130.7 | ± 14.3 |  |
| HDL cholesterol (mean, SD) (mg/dL) | 48.3 | ± 26.2 |  |
| Triglycerides (mean, SD) (mg/dL) | 137.0 | ± 76.1 |  |
| C-reactive protein (median, range) (mg/dL) | 0.50 | (0.04-4.40) | |
| Serum amyloid A (median, range) (mg/dL) | 0.59 | (0.05-19.21) | |

## Supporting Table 3 Primary and Secondary Antibodies Used for Immunohistochemistry.

| **Name** | **Dilution** | **Source** | **Catalog number** |
| --- | --- | --- | --- |
| **Human carotid plaque studies** |  |  |  |
| Mouse anti-FABP5 (primary) | 1:100 | Proteintech | 66299-1-Ig |
| Alexa Fluor 647 (red)-conjugated goat anti-mouse IgG (secondary) | 1:200 | Abcam | ab150115 |
| Rabbit anti-HM13/SPP (primary) | 1:100 | Bioss | BS-11178R |
| Alexa Fluor 488 (green)-conjugated goat anti-rabbit IgG (secondary) | 1:200 | Abcam | ab150077 |
| **Mouse atherosclerotic model studies** |  |  |  |
| Rabbit anti-α-SMA (primary) | 1:100 | Abcam | ab5694 |
| Alexa Fluor 555 (orange)-conjugated goat anti-rabbit IgG (secondary) | 1:200 | Abcam | ab150078 |
| Rat anti-Mac3 (primary) | 1:100 | BD Bio | 550292 |
| Biotinylated rabbit anti-rat IgG (secondary) | 1:200 | Vector Labs | BA-4000 |
| Alexa Fluor 647 (red)-conjugated mouse anti-Arg1 (primary) | 1:200 | SCB | sc-365547 |
| Alexa Fluor 488 (green)-conjugated rabbit anti-Nos2 (primary) | 1:200 | CST | 93421 |
| Rabbit anti-HO-1 (primary) | 1:100 | Abcam | ab13243 |
| Alexa Fluor 488 (green)-conjugated goat anti-rabbit IgG (secondary) | 1:200 | Abcam | ab150077 |
| Alexa Fluor 647 (red)-conjugated goat anti-rat IgG (secondary) | 1:200 | Abcam | ab150159 |

## Supporting Table 4 qPCR Primer Sequences.

| **Gene** | **Forward primer (5'→3')** | **Reverse primer (5'→3')** |
| --- | --- | --- |
| *Abca1* | GGCCAGTCTGTGTAACGGAT | TGCATCGAGCTTCTTCCTCG |
| *Abcg1* | AGGTCTCAGCCTTCTAAAGTTCCTC | TCTCTCGAAGTGAATGAAATTTATCG |
| *Acat1* | ATTTGCTGACGCTGCTGTAGA | AAGGCTTCATTTACTTCCCACATTG |
| *Adrp*/*Plin2* | GACAGGATGGAGGAAAGACTGC | GGTAGTCGTCACCACATCCTTC |
| *Ahr* | CTGGTTGTCACAGCAGATGCCT | CGGTCTTCTGTATGGATGAGCTC |
| *AHR* (human) | GTCGTCTAAGGTGTCTGCTGGA | CGCAAACAAAGCCAACTGAGGTG |
| *Aip*/*Ara9* | CAGCCTCTCATCTTCCACATCG | GTTGCCCTCTTGGTGGATGACT |
| *AIP*/*ARA9* (human) | TACTACGAGGTGCTGGACCACT | GCACTTTGGCAAAGTCAGCCTG |
| *Cd36* | ATGGGCTGTGATCGGAACTG | GTCTTCCCAATAAGCATGTCTCC |
| *FABP5* (human) | GGTGCATTGGTTCAGCATCAGG | TCATAGATCCGAGTACAGGTGAC |
| *Hm13* | CTAAGCAGCGTCGTTGGTGTCT | AGGATACAGCCAGTGCTCACGT |
| *HM13* (human) | GGACTTGGAGATGTCGTCATTCC | GCCGAAGATGTAGGCTGCAAAG |
| *Hmox1* | CACTCTGGAGATGACACCTGAG | GTGTTCCTCTGTCAGCATCACC |
| *HMOX1* (human) | CCAGGCAGAGAATGCTGAGTTC | AAGACTGGGCTCTCCTTGTTGC |
| *Il6* | TACCACTTCACAAGTCGGAGGC | CTGCAAGTGCATCATCGTTGTTC |
| *Lxra* | TCAGCATCTTCTCTGCAGACCGG | TCATTAGCATCCGTGGGAACA |
| *Mcp1* | GCTACAAGAGGATCACCAGCAG | GTCTGGACCCATTCCTTCTTGG |
| *OPN/SPP1* (human) | CGAGGTGATAGTGTGGTTTATGG | GCACCATTCAACTCCTCGCTTTC |
| *Mmp2* | CAAGGATGGACTCCTGGCACAT | TACTCGCCATCAGCGTTCCCAT |
| *Mmp9* | GCTGACTACGATAAGGACGGCA | TAGTGGTGCAGGCAGAGTAGGA |
| *Plin1* | GAGAAGGTGGTAGAGTTCCTCC | GTGTGTCGAGAAAGAGTGTTGGC |
| *Tnf* | GGTGCCTATGTCTCAGCCTCTT | GCCATAGAACTGATGAGAGGGAG |
| *Actb* (housekeeping) | GGGACCTGACAGACTACCTCATG | GTCACGCACGATTTCCCTCTCAGC |
| *ACTB* (housekeeping) | CACCATTGGCAATGAGCGGTTC | AGGTCTTTGCGGATGTCCACGT |
| *Gapdh* (housekeeping) | CATCACTGCCACCCAGAAGACTG | ATGCCAGTGAGCTTCCCGTTCAG |
| *GAPDH* (housekeeping) | GTCTCCTCTGACTTCAACAGCG | ACCACCCTGTTGCTGTAGCCAA |

## Supporting Table 5 Primary and Secondary Antibodies Used for Co-Immunoprecipitation (IP) and Immunoblotting (IB).

| **Name** | **Application: dilution** | **Source** | **Catalog number** |
| --- | --- | --- | --- |
| Rabbit anti-ABCA1 | IB: 1:500 | Abcam | NB400-105 |
| Rabbit anti-ABCG1 | IB: 1:500 | Abcam | NB400-132 |
| Rabbit anti-AIP | IB: 1:500 | Abcam | ab228684 |
| Rabbit anti-AHR | IB: 1:500 | Novus Bio | NB100-2289 |
| Rabbit anti-CANX | IB: 1:2000 | Abcam | ab13504 |
| Rabbit anti-CD36 | IB: 1:500 | Novus Bio | NB400-144 |
| Rabbit anti-phospho-c-JUN^S63^ | IB: 1:1000 | Abcam | ab32385 |
| Rabbit anti-c-JUN | IB: 1:500 | Abcam | ab31419 |
| Rabbit anti-Col I (COL1A1) | IB: 1:500 | Abcam | ab21286 |
| Rabbit anti-Col III (COL3A1) | IB: 1:500 | Abcam | ab184993 |
| Rabbit anti-DERLIN-1 | IB: 1:500 | Abcam | ab176732 |
| Rabbit anti-elastin | IB: 1:500 | Abcam | ab307150 |
| Rabbit anti-FLAG (DYKDDDDK tag) | IB: 1:500 | Sigma | F7425 |
| Rabbit anti-HA (hemagglutinin tag) | IB: 1:4000;  IP: see Methods | Abcam | ab9110 |
| Rabbit anti-HO-1 | IB: 1:2000 | Abcam | ab13243 |
| Rabbit anti-HM13 | IB: 1:500 | Bioss | BS-11178R |
| Rabbit anti-phospho-JNK^T183/Y185^ | IB: 1:500 | Abcam | ab4821 |
| Rabbit anti-JNK | IB: 1:500 | Abcam | ab112501 |
| Rabbit anti-HO-1 | IB: 1:2000 | Abcam | ab13243 |
| Rabbit anti-HM13 | IB: 1:500 | Bioss | BS-11178R |
| Rabbit anti-IL-1β | IB: 1:500 | Abcam | ab234437 |
| Rabbit anti-IL-6 | IB: 1:500 | Abcam | ab259341 |
| Rabbit anti-LDLR | IB: 1:500 | Abcam | ab52818 |
| Rabbit anti-MMP-9 | IB: 1:500 | Abcam | ab283575 |
| Rabbit anti-RNF139 | IB: 1:500 | Sigma | SAB2102019 |
| Rabbit anti-SR-A | IB: 1:500 | Abcam | NBP1-00092 |
| Rabbit anti-phospho-p38^T180/Y182^ | IB: 1:1000 | Abcam | ab4822 |
| Rabbit anti-p38 | IB: 1:1000 | Abcam | ab170099 |
| Rabbit anti-SEC61α | IB: 1:500 | ProteinTech | 24935-1-AP |
| Rabbit anti-α-tubulin (whole-cell loading control) | IB: 1:500 | Abcam | ab4074 |
| HRP-conjugated goat anti-rabbit IgG (secondary) | IB: 1:2000 | Abcam | ab6721 |

## Supporting Table 6 Results of the Differential Co-Expression Analysis.

The table depicts module size, the average (median) change in modular differential connectivity (MeDC) between the two conditions for each module, and the associated p-values. The last two columns show the top three genes (if any) with significant gain (Top_GOC) and loss (Top_LOC) in connectivity.

| **Module** | **Size** | **MeDC** | ***P*-value** | **Top_GOC** | **Top_LOC** |
| --- | --- | --- | --- | --- | --- |
| Black | 232 | -0.9992 | 0.04 | None | *NFYA, SLC12A6, SNX5* |
| Blue | 1500 | -0.2251 | 0.41 | *NDFIP1, CREBZF, EMC8* | *MED30, PHETA1, C17orf100* |
| Brown | 1108 | -0.8849 | 0.10 | *RORA, IMPACT, CDC14A* | *PLXDC2, DCAF8, NDFIP1* |
| Green | 532 | -0.1682 | 0.46 | *UBE2L6, DNAJC9* | *PDXP\|SH3BP1, LARP7, SEZ6L* |
| Grey | 2404 | -0.4769 | 0.00 | *RORA, DAB2, PDE4D* | *THAP7-AS1, SVEP1, SVEP1* |
| Magenta | 155 | -0.1463 | 0.63 | *PKN2, CRABP2, RCAN3* | None |
| Pink | 191 | -0.1687 | 0.69 | None | None |
| Red | 279 | -0.0775 | 0.92 | None | None |
| Turquoise | 1870 | -0.3943 | 0.31 | *MIF4GD, ADH1B* | *HNF4A, EIF4E2, LINC00643* |
| Yellow | 662 | -0.1033 | 0.79 | *NANS, ZMAT1, DDN* | *POLR1B, APBB2, SPNS3* |

## Supporting Table 7 Results of the Module Preservation Analysis.

NetRep-based calculation of seven module preservation parameters for each of the ten modules. Please see the Methods for detailed descriptions of these parameters. Asterisks (*) indicate modules wherein *P*<0.05 across all seven parameters.

| **Liver** | **avg.weight** | **coherence** | **cor.cor** | **cor.degree** | **cor.contrib** | **avg.cor** | **avg.contrib** |
| --- | --- | --- | --- | --- | --- | --- | --- |
| **module** |  |  |  |  |  |  |  |
| Black* | 0.001 | 0.001 | 0.001 | 0.001 | 0.001 | 0.001 | 0.001 |
| Blue* | 0.001 | 0.001 | 0.001 | 0.001 | 0.001 | 0.001 | 0.001 |
| Brown* | 0.001 | 0.001 | 0.001 | 0.001 | 0.001 | 0.001 | 0.001 |
| Green | 1.000 | 1.000 | 0.001 | 0.158 | 0.001 | 0.987 | 0.916 |
| Grey | 1.000 | 1.000 | 0.001 | 0.001 | 0.001 | 0.001 | 0.001 |
| Magenta | 0.800 | 1.000 | 0.001 | 0.012 | 0.001 | 0.060 | 0.018 |
| Pink* | 0.001 | 0.001 | 0.001 | 0.001 | 0.001 | 0.001 | 0.001 |
| Red* | 0.001 | 0.001 | 0.001 | 0.001 | 0.001 | 0.001 | 0.001 |
| Turquoise* | 0.001 | 0.001 | 0.001 | 0.001 | 0.001 | 0.001 | 0.001 |
| Yellow | 1.000 | 1.000 | 0.001 | 0.235 | 0.021 | 0.131 | 0.010 |
| **Skeletal muscle** | **avg.weight** | **coherence** | **cor.cor** | **cor.degree** | **cor.contrib** | **avg.cor** | **avg.contrib** |
| **module** |  |  |  |  |  |  |  |
| Black* | 0.001 | 0.001 | 0.001 | 0.001 | 0.001 | 0.001 | 0.001 |
| Blue* | 0.001 | 0.001 | 0.001 | 0.001 | 0.001 | 0.001 | 0.001 |
| Brown* | 0.001 | 0.001 | 0.001 | 0.001 | 0.001 | 0.001 | 0.001 |
| Green | 0.997 | 1.000 | 0.001 | 0.002 | 0.001 | 0.001 | 0.001 |
| Grey | 1.000 | 1.000 | 0.001 | 0.001 | 1.000 | 0.001 | 1.000 |
| Magenta | 0.192 | 0.326 | 0.001 | 0.673 | 0.478 | 0.001 | 0.001 |
| Pink* | 0.001 | 0.001 | 0.001 | 0.001 | 0.001 | 0.001 | 0.001 |
| Red* | 0.001 | 0.001 | 0.001 | 0.001 | 0.001 | 0.001 | 0.001 |
| Turquoise* | 0.001 | 0.001 | 0.001 | 0.001 | 0.001 | 0.001 | 0.001 |
| Yellow | 0.004 | 0.576 | 0.001 | 0.002 | 0.029 | 0.001 | 0.001 |
| **Visceral fat** | **avg.weight** | **coherence** | **cor.cor** | **cor.degree** | **cor.contrib** | **avg.cor** | **avg.contrib** |
| **module** |  |  |  |  |  |  |  |
| Black* | 0.001 | 0.001 | 0.001 | 0.001 | 0.001 | 0.001 | 0.001 |
| Blue* | 0.001 | 0.001 | 0.001 | 0.001 | 0.001 | 0.001 | 0.001 |
| Brown* | 0.001 | 0.001 | 0.001 | 0.001 | 0.001 | 0.001 | 0.001 |
| Green | 1.000 | 0.964 | 0.001 | 0.079 | 0.822 | 0.179 | 0.993 |
| Grey | 1.000 | 1.000 | 0.001 | 0.001 | 1.000 | 0.001 | 1.000 |
| Magenta | 0.022 | 0.150 | 0.001 | 0.220 | 0.639 | 0.002 | 0.702 |
| Pink* | 0.001 | 0.001 | 0.001 | 0.001 | 0.032 | 0.001 | 0.001 |
| Red* | 0.001 | 0.001 | 0.001 | 0.001 | 0.001 | 0.001 | 0.001 |
| Turquoise* | 0.001 | 0.001 | 0.001 | 0.001 | 0.001 | 0.001 | 0.001 |
| Yellow | 0.001 | 0.001 | 0.001 | 0.002 | 0.001 | 0.001 | 0.001 |

# Supporting Figure Legends

**Supporting Figure 1 Selection of the Soft Threshold for Weighted Gene Co-Expression Network Analysis (WGCNA) Network Construction.** A soft-threshold power of eight achieved approximate scale-free topology (*R*^2^>0.8).

**Supporting Figure 2 Simulation of Different Combinations of Hyperparameters.** Plot demonstrating that the WGCNA modules are relatively robust to changes in hyperparameters. A deepSplit of zero seems to be more influenced by the minimum module size parameter. Additional modules are detected with increased deepSplit regardless of module size and merge threshold. The inclusion of a pamStage detection step seems to introduce noise regardless of sensitivity and module size.

**Supporting Figure 3** **Selection of the Final Parameters for Weighted Gene Co-Expression Network Analysis (WGCNA) Network Construction.** **a)** Cluster dendrogram corresponding to the final WGCNA network. Based on the iteration results, the final WGCNA network was built with a deepSplit of 2 (for higher sensitivity) and a minimum module size of 100 (to avoid capturing potential noise with the module size of 50). PamStage was kept as FALSE and mergeCutHeight at 0.25. **b)** Number of features per module in the final WGCNA network.

**Supporting Figure 4 Enrichment Maps for the *AIP*-Associated Blue Module in Human Atherosclerotic Plaques.** **a)** BioCarta, **b)** Gene Ontology Biological Process (GO-BP), **c)** KEGG, and **d)** Reactome enrichment maps for the Blue module in the STAGE atherosclerotic plaque cohort (GSE40231).

**Supporting Figure 5 Enrichment Maps for the *AIP*-Associated Brown Module in Human Atherosclerotic Plaques.** **a)** BioCarta, **b)** Gene Ontology Biological Process (GO-BP), **c)** KEGG, and **d)** Reactome enrichment maps for the Brown module in the STAGE atherosclerotic plaque cohort (GSE40231).

**Supporting Figure 6 Enrichment Maps for the *AIP*-Associated Turquoise Module in Human Atherosclerotic Plaques.** **a)** BioCarta, **b)** Gene Ontology Biological Process (GO-BP), **c)** KEGG, and **d)** Reactome enrichment maps for the Turquoise module in the STAGE atherosclerotic plaque cohort (GSE40231).

**Supporting Figure 7 Construction and Correlation Analyses of the Macrophage Plaque Gene Signature Score. a)** The heatmap below shows the fifteen genes selected to calculate the Macrophage Plaque Gene Signature Score (grey) in the coronary atherosclerotic plaque cohort (GEO acc. no. GSE11138). The Module Hub Gene Signature Scores for the Blue, Brown, and Turquoise modules (each labelled with their respective colors) are provided for comparison. **b)** Correlation plots of the Module Hub Gene Signature Scores for the Blue, Brown, and Turquoise modules with the Macrophage Plaque Gene Signature Score in the STAGE atherosclerotic plaque cohort (GSE40231).

**Supporting Figure 8 Blue Module Gene Heatmap and Correlation Analyses between the Three Highest-Ranking Blue Gene Module Members and *AIP*. a)** Heatmap of the 47 highest-ranking Blue module genes (by module membership) along with the Macrophage Plaque Gene Signature Score and all three Module Hub Gene Signature Scores in the coronary atherosclerotic plaque cohort (GEO acc. no. GSE11138). **b)** Correlation plots demonstrating negative correlations between *AIP* and the three highest-ranking Blue module genes that negatively correlated with *AIP* in the STAGE atherosclerotic plaque cohort (GSE40231).

**Supporting Figure 9 scRNAseq Analysis of *AIP* and *HM13* Co-Expression Across Human Atherosclerotic Plaque Immune Cell Types.** scRNAseq analysis of human atherosclerotic carotid plaque scRNAseq data (*n*=6 patients; GSE224273). **a, b)** Uniform manifold approximation and projection (UMAP) visualizations depicting **a)** the plaque immune cell clusters and **b)** *AIP* and *HM13* expression across the plaque immune cell clusters. **c)** Violin plots depicting *AIP* and *HM13* expression across the plaque immune cell clusters.

**Supporting Figure 10 scRNAseq Analysis Identifies Four Distinct Myeloid Clusters in Human Atherosclerotic Plaques.** scRNAseq analysis of human atherosclerotic carotid plaque scRNAseq data (*n*=6 patients; GSE224273). **a)** Uniform manifold approximation and projection (UMAP) visualizations depicting subsetting and re-clustering of the original plaque myeloid clusters. This defined four distinct clusters of plaque myeloid cells: monocytes (70 cells), resident macrophages (61 cells), inflammatory macrophages (84 cells), and foamy macrophages (92 cells). **b)** Heatmap depicting the average expression of the top five markers for each plaque myeloid cluster. Note the presence of the dysfunctional foamy macrophage markers *FABP5* and *OPN* (*SPP1*) in the foamy macrophage cluster.

**Supporting Figure 11 scRNAseq Analysis Across the M1 (Inflammatory)-M2 (Anti-Inflammatory) Macrophage Polarization Spectrum. a)** Uniform manifold approximation and projection (UMAP) visualizations displaying the four final plaque myeloid clusters (left) and the six polarized macrophage clusters (right). **b)** Violin plots depicting *AIP* and *HM13* expression across the six polarized macrophage clusters.

**Supporting Figure 12 Validation of oxLDL-Induced Lipid Loading *In Vitro.*** Murine bone marrow-derived macrophages (mBMDMs) were analysed after incubation with DMSO vehicle, nLDL (25 µg/ml), or oxLDL (25 µg/ml) for 24 h. **a, b)** Intracellular content of **a)** total cholesterol (TC), unesterified free cholesterol (FC), and cholesteryl esters (CE) and **b)** triglycerides. **c)** Representative images and quantification of Oil Red O-staining in mBMDMs (scale bar, 50 µm). *n*=6 independent biological replicates per cohort. Data expressed as means ± SDs compared using one-way ANOVA. **P*<0.05, ***P*<0.01.

**Supporting Figure 13 Correlation Analyses in Human Coronary Plaque Foamy Macrophages. a,b** Pearson correlation analyses of *AIP* gene expression with **a)** *FABP5* gene expression in coronary plaque FABP5^+^CD45^+^CD68^+^ foamy macrophages and **b)** *OPN* gene expression in coronary plaque OPN^+^CD45^+^CD68^+^ foamy macrophages. **c,d)** Pearson correlation analyses of *HM13* gene expression with **c)** *FABP5* gene expression in coronary plaque FABP5^+^CD45^+^CD68^+^ foamy macrophages and **d)** *OPN* gene expression in coronary plaque OPN^+^CD45^+^CD68^+^ foamy macrophages.

**Supporting Figure 14 *HM13* Promoter Analyses Identify a Strongly-Conserved Binding Site for c-JUN. a)** Cartoon depicting the c-JUN binding motif (Matrix ID: MA0489.1). **b)** A strongly-conserved c-JUN binding motif (Matrix ID: MA0489.1) identified approx. 250 bp upstream of the *HM13* transcriptional start site (TSS) (indicated by black arrow).

**Supporting Figure 15 Generation of Myeloid-Specific Transgenic *Hm13* Mouse Strains. a)** We crossed *Hm13*-floxed (*Hm13*^fl/fl^) mice and *Lyz2-2A*^Cre^ mice to create *Hm13*^mKO^ (*Hm13* myeloid knockout) mice. **b)** *Hm13*^mOE^ (*Hm13* myeloid overexpression) mice were produced by the first construct. Then, excision of the STOP cassette mediated by Cre produced an *Hm13*-eGFP bicistronic transcript. Transcription from the endogenous *Rosa26* promoter indicated by a bent arrow. Abbreviations: Flippase recognition target (FRT); splice acceptor (SA); polyadenylation motif (pA); internal ribosome entry site (IRES); LacZ encoding β-galactosidase; neomycin resistance gene (Neo).

**Supporting Figure 16 Modulation of HM13/SPP expression in Myeloid-Specific Transgenic *Hm13* Mouse Strains. a, b)** In mBMDMs from *Hm13*^fl/fl^, *Hm13*^mKO^ (*Hm13* myeloid knockout), and *Hm13*^mOE^ (*Hm13* myeloid overexpression) mice, **a)** qPCR analysis of *Hm13* mRNA expression and **b)** Western blotting analysis of HM13/SPP protein expression. **c-f)** Western blotting analysis of HM13/SPP protein expression in **c)** monocytes, **d)** basophils, **e)** eosinophils, and **f)** neutrophils isolated from *Hm13*^fl/fl^, *Hm13*^mKO^, and *Hm13*^mOE^ mice. *n*=6 mice per cohort. Data expressed as means ± SDs compared using one-way ANOVA. **P*<0.05, ***P*<0.01.

**Supporting Figure 17 Characterization of Myeloid-Specific Transgenic *Hm13* Mouse Strains.**

**a)** Flow cytometric quantification of *Hm13*-GFP^+^ cells within the monocytic Cd11b^+^/Ly6c^+^ myeloid cell populations isolated from *Hm13*^fl/fl^ and *Hm13*^mOE^ murine blood samples and bone marrow samples. **b)** Blood cell counts from *Hm13*^fl/fl^, *Hm13*^mKO^, and *Hm13*^mOE^ mice. c) Representative H&E staining images of visceral adipose tissue cross-sections from 10-week old mice fed on a chow diet. The mean adipocyte area and F4/80^+^ macrophage % (as a % of all nucleated cells) values are reported. Scale bar, 20 μm. d) Representative H&E staining images of liver cross-sections from 10-week old mice fed on a chow diet. F4/80^+^ macrophage % (as a % of all nucleated cells) values are reported. Scale bar, 20 μm. *n*=6 mice per cohort. Data expressed as means ± SDs compared using **b-d)** one-way ANOVA. **P*<0.05, ***P*<0.01.

**Supporting Figure 18 Macrophage HM13/SPP Not Associated with HDL-Mediated Cholesterol Efflux.** The following studies were performed in cholesterol-loaded *Hm13*^fl/fl^, *Hm13*^mKO^, and *Hm13*^mOE^ murine bone marrow-derived macrophages (mBMDMs). **a)** Quantification of cholesterol efflux to human HDL. **b)** qPCR analyses of the cholesterol efflux genes *Abca1* and *Abcg1* as well as **c)** atherosclerosis-associated cholesterol metabolism genes *Acat1*, *Adrp* (*Plin2*), *Cd36*, *Lxra*, and *Plin1*. *n*=6 independent biological replicates per cohort. Data expressed as means ± SDs compared using **a)** two-way ANOVA; **b, c)** one-way ANOVA. **P*<0.05, ***P*<0.01.

**Supporting Figure 19 Macrophage HM13/SPP Associated with Pro-Inflammatory Properties.** *Hm13*^fl/fl^, *Hm13*^mKO^, and *Hm13*^mOE^ mBMDMs were analysed after incubation with oxLDL (25 µg/ml) for 24 h. **a)** qPCR analyses of the inflammatory cytokine genes *Il6, Mcp1*, and *Tnf*. **b)** ELISA of the secreted inflammatory cytokines IL-6, MCP-1, and TNF-α using the mBMDM culture supernatant. *n*=6 independent biological replicates per cohort. Data expressed as means ± SDs compared using one-way ANOVA. **P*<0.05, ***P*<0.01.

**Supporting Figure 20 Analysis of Plasma Lipid Levels in Western Diet-Fed *ApoE*^−/−^ Chimeric Mice.** Plasma lipid levels were analysed in *ApoE*^−/−^ chimeric mice following seven weeks of recovery and twelve weeks on Western Diet. **a)** Total cholesterol (TC), **b)** high-density lipoprotein cholesterol (HDL-C), **c)** low-density lipoprotein cholesterol (LDL-C), and **d)** triglycerides. *n*=12 mice per cohort. Data expressed as medians ± upper/lower quartiles compared using Kruskal-Wallis test. **P*<0.05, ***P*<0.01.

**Supporting Figure 21 LDLR Knockdown and Plasma Lipid Levels in Western Diet-Fed rAAV8-*Pcsk9* Model Mice. a)** Western blotting was used to quantify LDLR protein levels in liver samples from rAAV8-*Pcsk9* mice following twelve weeks on Western Diet. **b-e)** Plasma lipid levels were analysed in rAAV8-*Pcsk9* mice following twelve weeks on Western Diet. **b)** Total cholesterol (TC), **c)** high-density lipoprotein cholesterol (HDL-C), **d)** low-density lipoprotein cholesterol (LDL-C), and **e)** triglycerides. *n*=12 mice per cohort. Data expressed as means ± SDs compared using **a)** two-way ANOVA; medians ± upper/lower quartiles compared using **b-e)** Kruskal-Wallis test. **P*<0.05, ***P*<0.01.

**Supporting Figure 22 Foamy Macrophage Counts, Foamy Macrophage Sizes, and Aortic Sinus Plaque Areas Not Correlated with Total Cholesterol Levels in Western Diet-Fed Mouse Models. a, b)** Analyses performed on *ApoE*^−/−^ chimeric mice following seven weeks of recovery and twelve weeks on Western Diet. **a)** Pearson correlations between plasma total cholesterol levels (*x*-axis) and mean foamy macrophage counts (*y*-axis, black circles) and mean foamy macrophage sizes (*y*-axis, blue squares) in aortic sinus lesions. **b)** Pearson correlation between plasma total cholesterol levels (*x*-axis) and aortic sinus lesion areas (*y*-axis). **c, d)** Analyses performed on rAAV8-*Pcsk9* mice following twelve weeks on Western Diet. **c)** Pearson correlations between plasma total cholesterol levels (*x*-axis) and mean foamy macrophage counts (*y*-axis, black circles) and mean foamy macrophage sizes (*y*-axis, blue squares) in aortic sinus lesions. **d)** Pearson correlation between plasma total cholesterol levels (*x*-axis) and aortic sinus lesion areas (*y*-axis). Data shows correlation coefficient (*r*) along with *P-*value.

**Supporting Figure 23 The ERAD Proteins RNF139 and DERLIN-1 Are Necessary For HO-1 Degradation. a)** Following 24-h hemin (50 µM) stimulation, membrane fraction inputs from transduced THP-1 cells were immunoprecipitated with an anti-HA antibody (IP:HA) and then subjected to immunoblotting analysis. **b)** Whole cell lysates from transduced THP-1 cells were subjected to immunoblotting analysis with an anti-HA antibody (IB:HA) or an anti-FLAG antibody (IB:FLAG). **c)** Cycloheximide (CHX, 2 μg/mL) chase studies in transduced THP-1 cells following 24-h hemin (50 µM) stimulation. **d)** Pulse-chase analysis of HO-1 cleavage in transduced THP-1 cells by HO-1 immunoprecipitation with an anti-FLAG antibody (IP:FLAG) without or with epoxomicin (1 μM). At 0 h and 1 h, post-nuclear lysates were separated into membrane (Mem) fractions and cytosolic (Cyto) fractions prior to FLAG immunoprecipitation and SDS-PAGE autoradiography. *n*=6 independent biological replicates per cohort. Data expressed as means ± SDs compared using two-way ANOVA. **P*<0.05, ***P*<0.01.

**Supporting Figure 24 The Macrophage HM13/SPP-HO-1 Axis Does Not Impact mRNA Expression of *Abca1* or *Abcg1*.** *Hm13*^fl/fl^, *Hm13*^mKO^, *Hm13*^mOE^, and *Hm13*^mOE^;*Hmox1*^mOE^ murine bone marrow-derived macrophages (mBMDMs) were subjected to qPCR analyses of the cholesterol efflux genes **a)** *Abca1* and **b)** *Abcg1* after incubation with oxLDL (25 µg/ml) without or with ZnPP (10 µM) for 24 h. *n*=6 independent biological replicates per cohort. Data expressed as means ± SDs compared using one-way ANOVA. **P*<0.05, ***P*<0.01.

**Supporting Figure 25 Macrophage HM13/SPP Enhances Pro-Inflammatory Properties Through HO-1 Degradation.** *Hm13*^fl/fl^, *Hm13*^mKO^, *Hm13*^mOE^, and *Hm13*^mOE^;*Hmox1*^mOE^ murine bone marrow-derived macrophages (mBMDMs) were analysed after incubation with oxLDL (25 µg/ml) without or with ZnPP (10 µM) for 24 h. ELISA of the secreted inflammatory cytokines **a)** IL-6, **b)** MCP-1, and **c)** TNF-α using the mBMDM culture supernatant. *n*=6 independent biological replicates per cohort. Data expressed as means ± SDs compared using one-way ANOVA. **P*<0.05, ***P*<0.01.

**Supporting Figure 26 Macrophage HM13/SPP Induces Phenotypic Changes in Co-Cultured Vascular Smooth Muscle Cells Through HO-1 Degradation.** *Hm13*^fl/fl^, *Hm13*^mKO^, *Hm13*^mOE^, and *Hm13*^mOE^;*Hmox1*^mOE^ murine bone marrow-derived macrophages (mBMDMs) were incubated with oxLDL (25 µg/ml) without or with ZnPP (10 µM) for 24 h. These oxLDL-treated mBMDMs and murine VSMCs were then co-cultured (∪) for 72 h in a macrophage-VSMC Transwell co-culture system without or with ZnPP (10 µM) as indicated. **a)** qPCR analyses of the matrix metalloproteinase genes *Mmp2* and *Mmp9*. **b)** Western blotting analysis of MMP-9 protein expression. **c)** MMP-9 activity assay utilizing a cleavable, chromogenic peptide substrate that emits a 405-nm signal. **d)** Western blotting analysis of the inflammatory cytokines IL-1β and IL-6. **e)** Western blotting analysis of the extracellular matrix proteins collagen I, III, and elastin. *n*=6 independent biological replicates per cohort. Data expressed as means ± SDs compared using one-way ANOVA. **P*<0.05, ***P*<0.01.

# Supporting Figures

## Supporting Figure 1


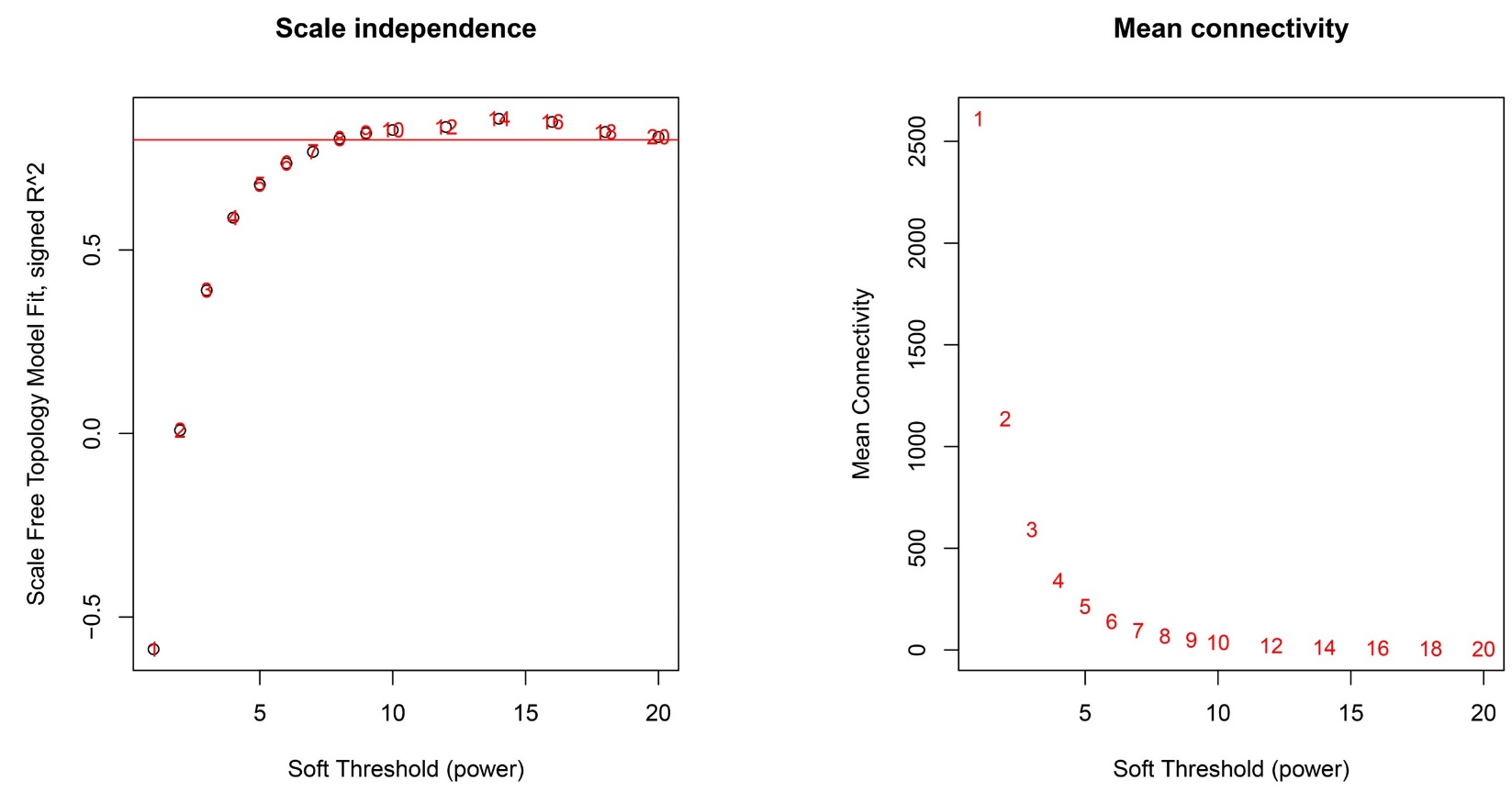


Supporting Figure 2

**
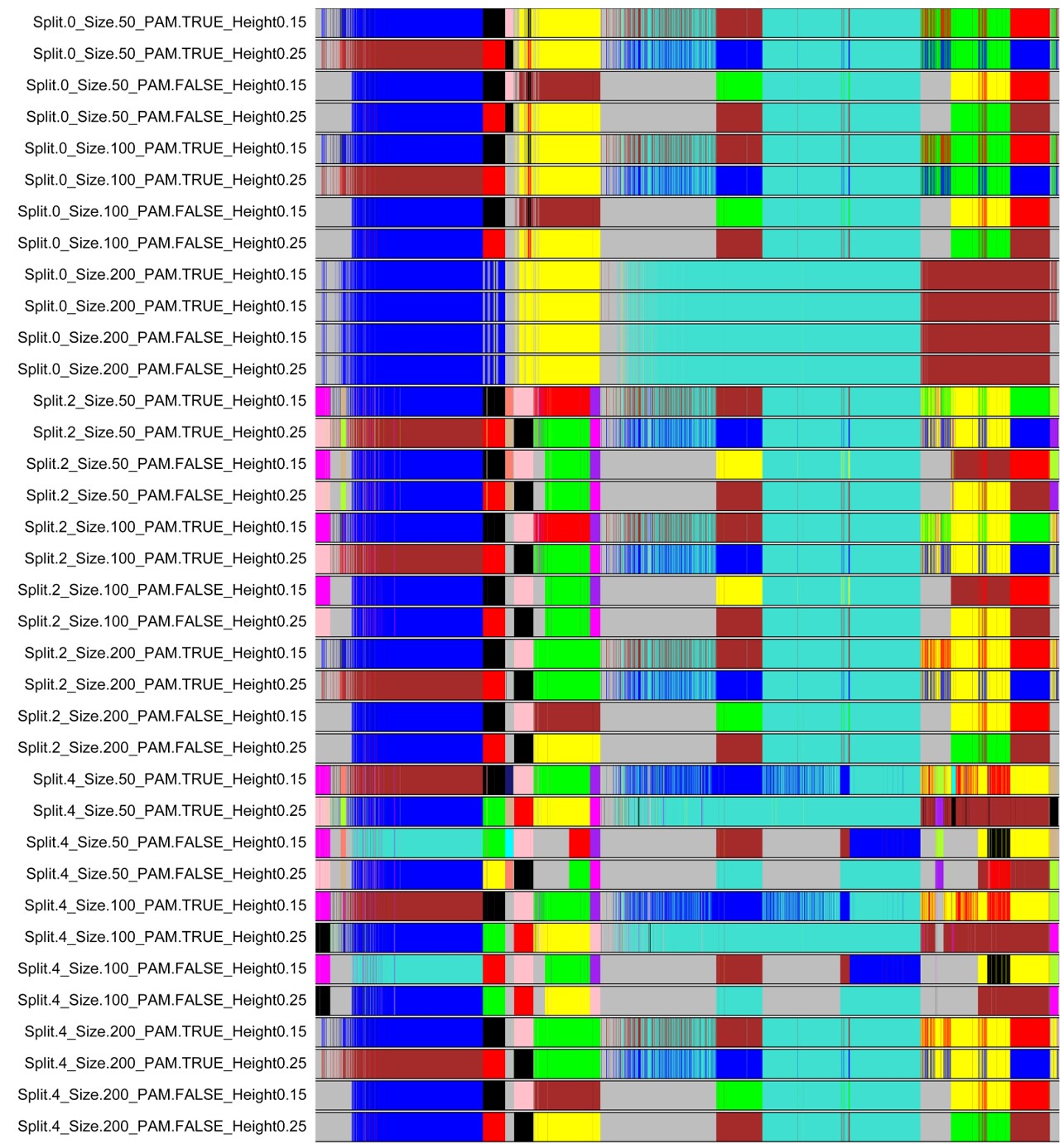
**

Supporting Figure 3


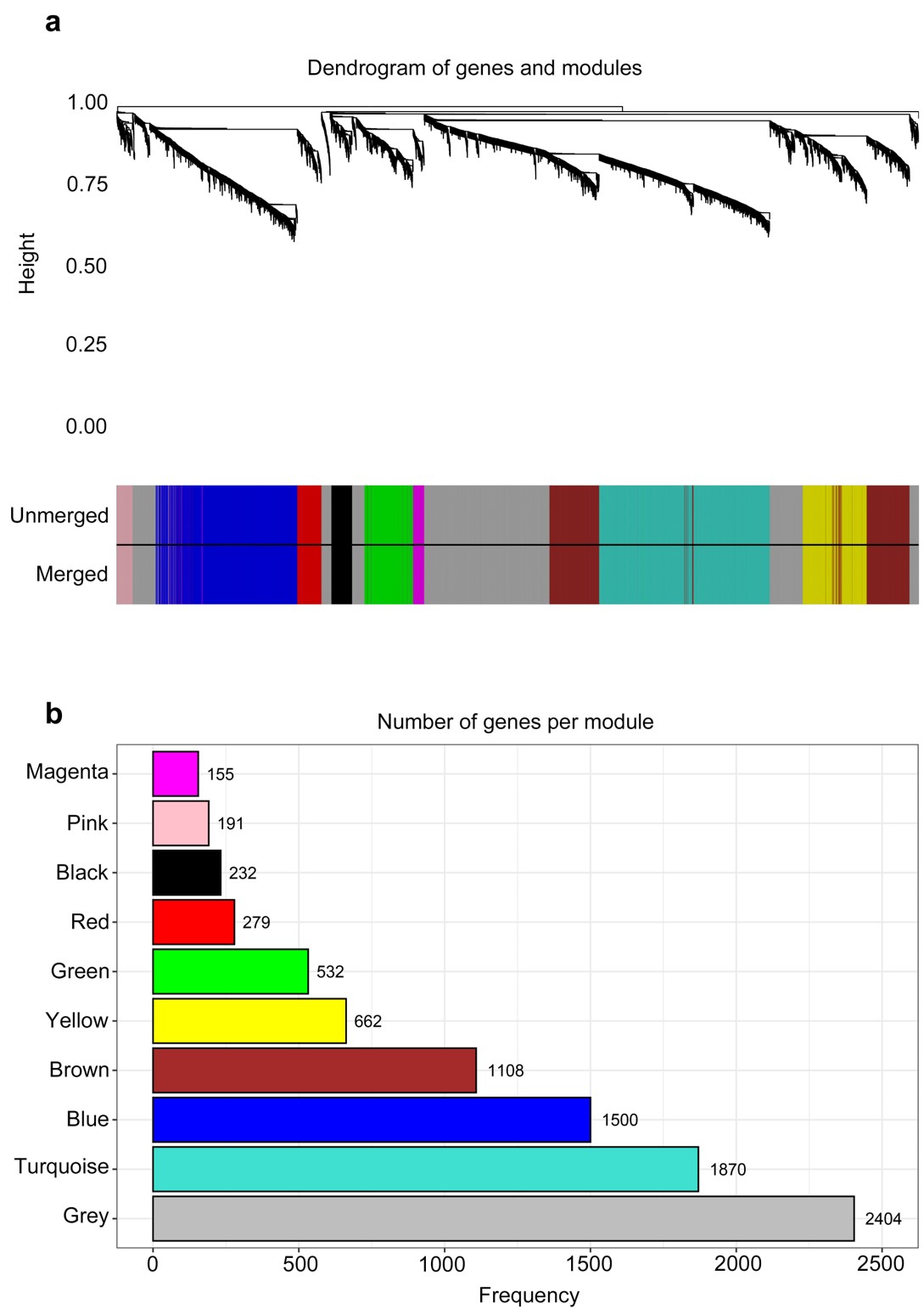


## Supporting Figure 4

**
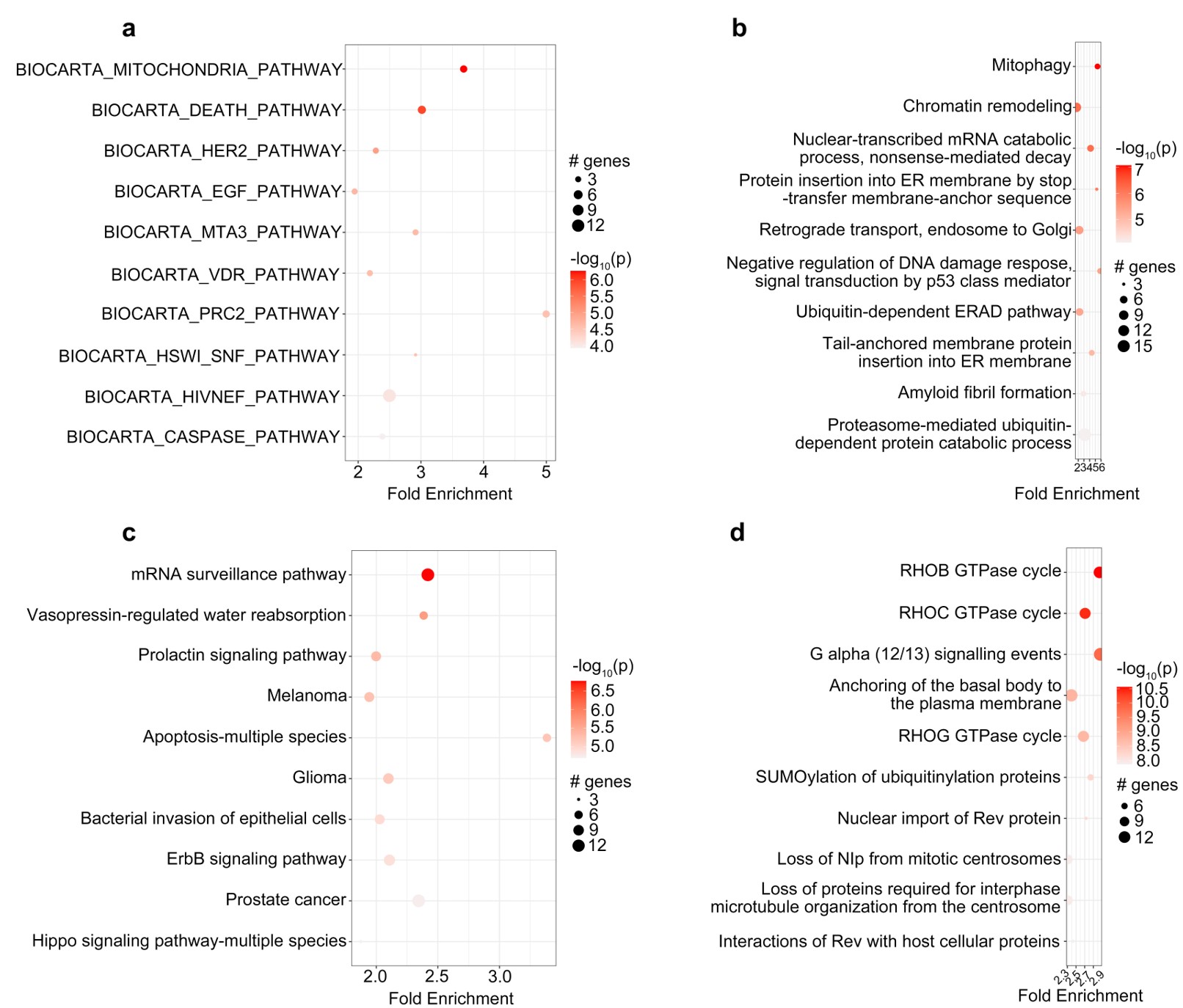
**

## Supporting Figure 5


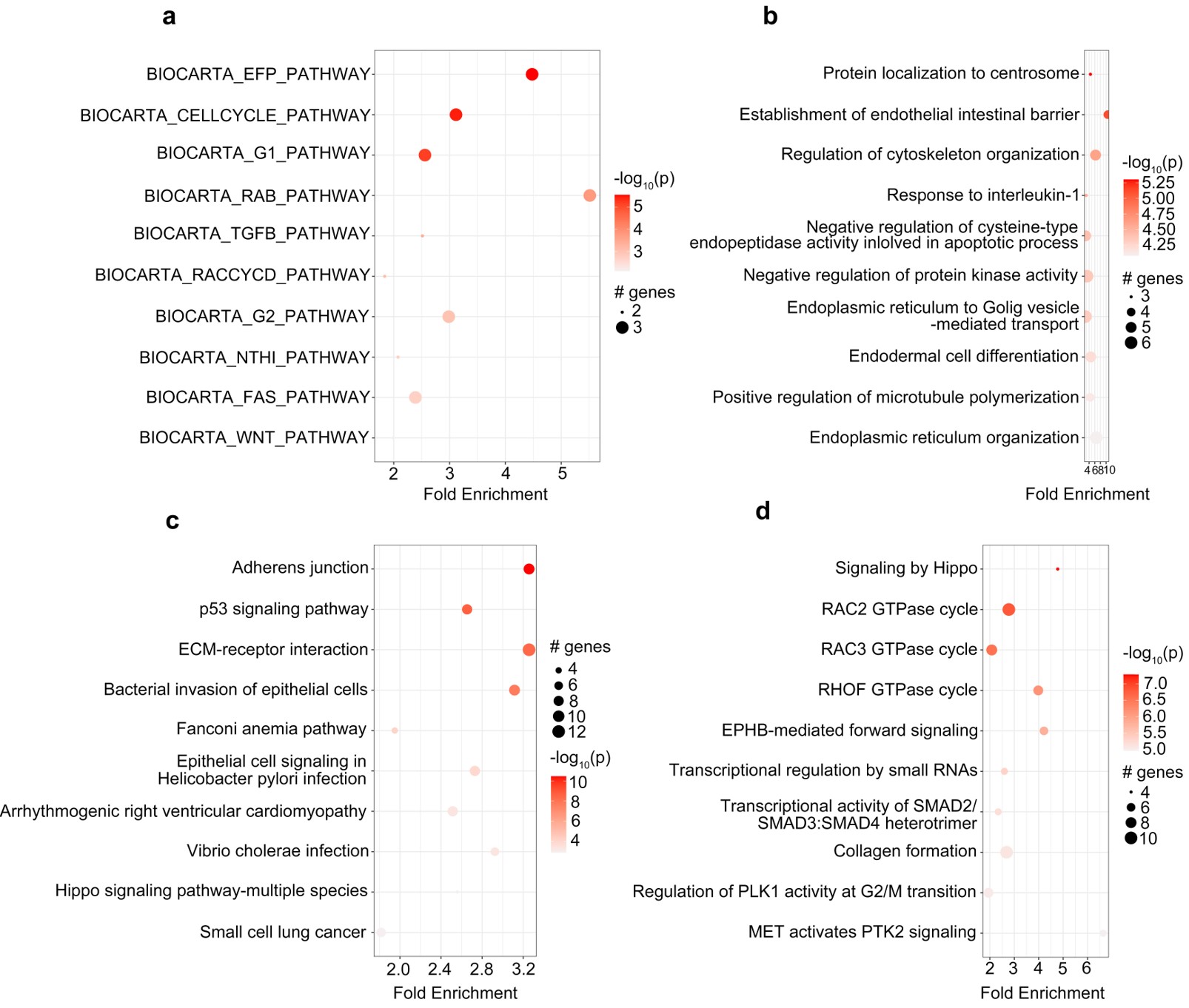


## Supporting Figure 6


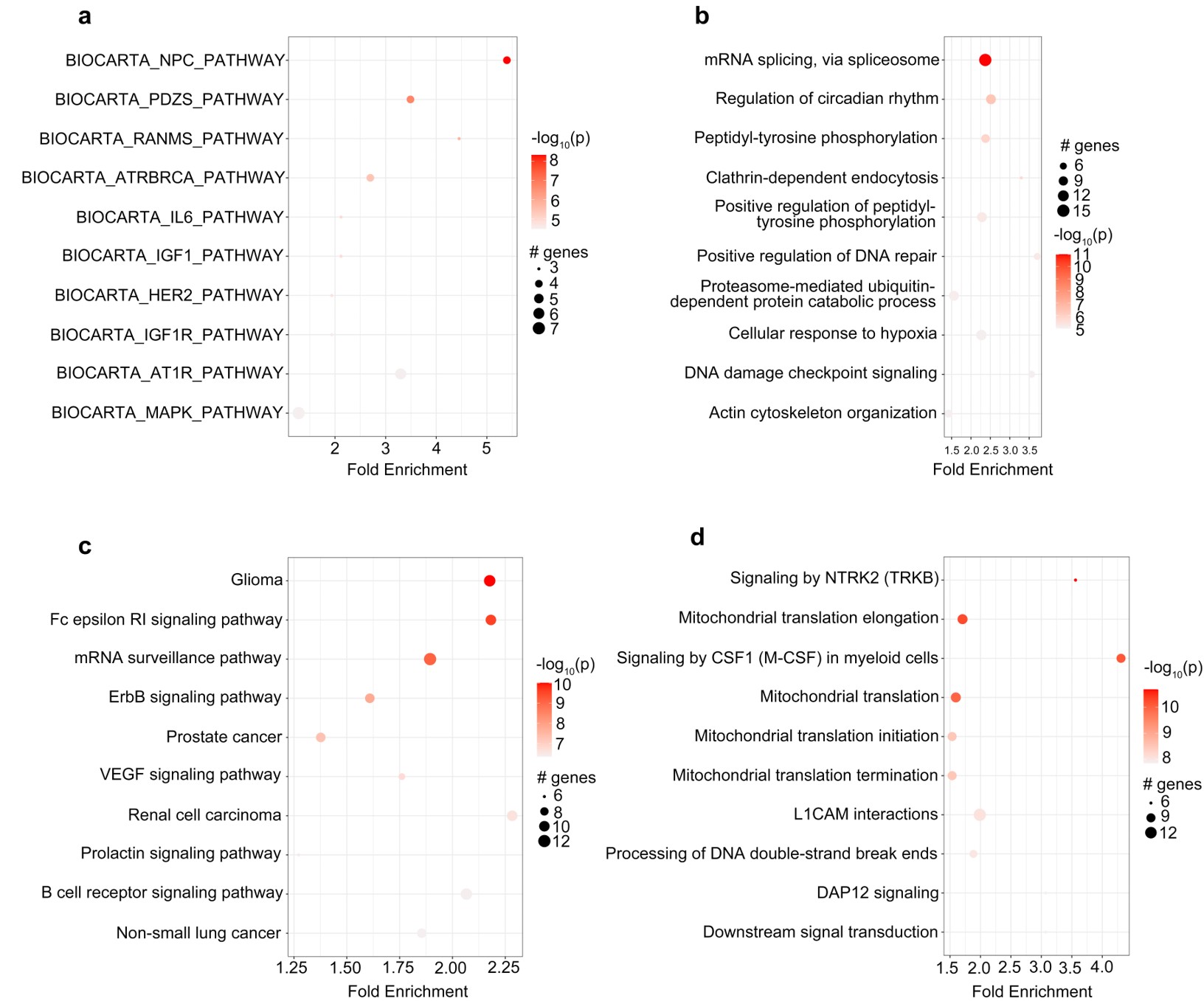


## Supporting Figure 7


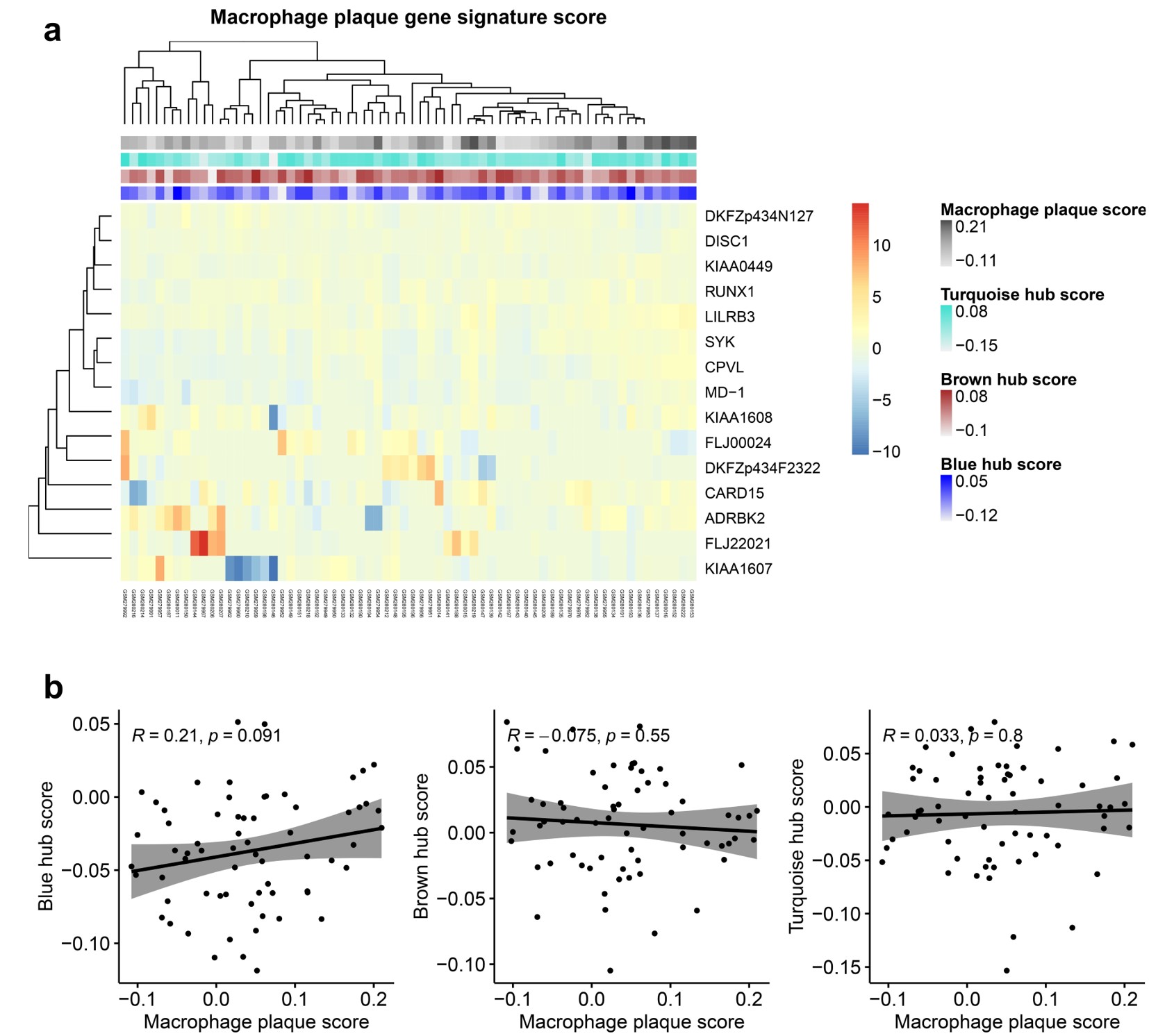


## Supporting Figure 8


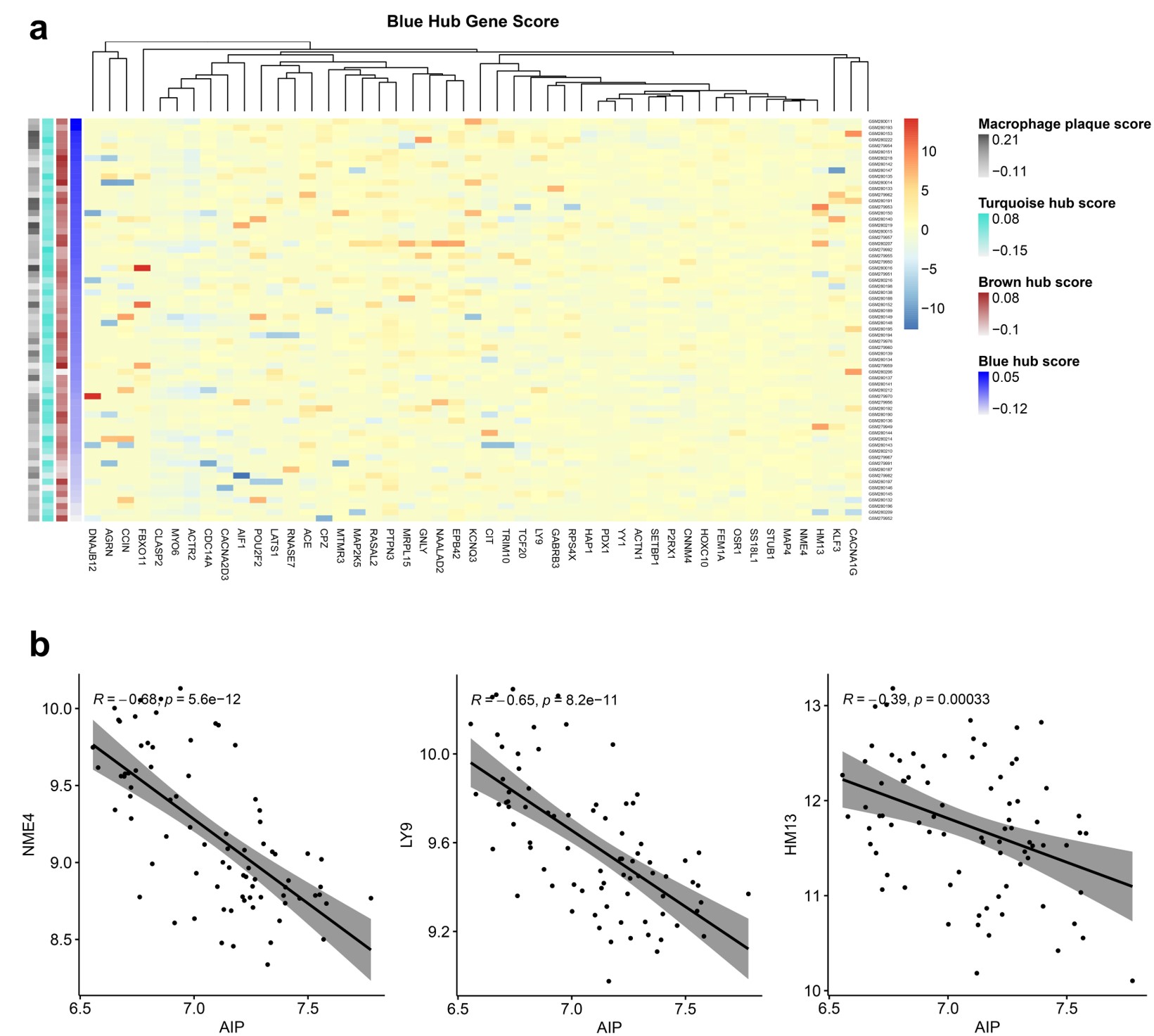


## Supporting Figure 9


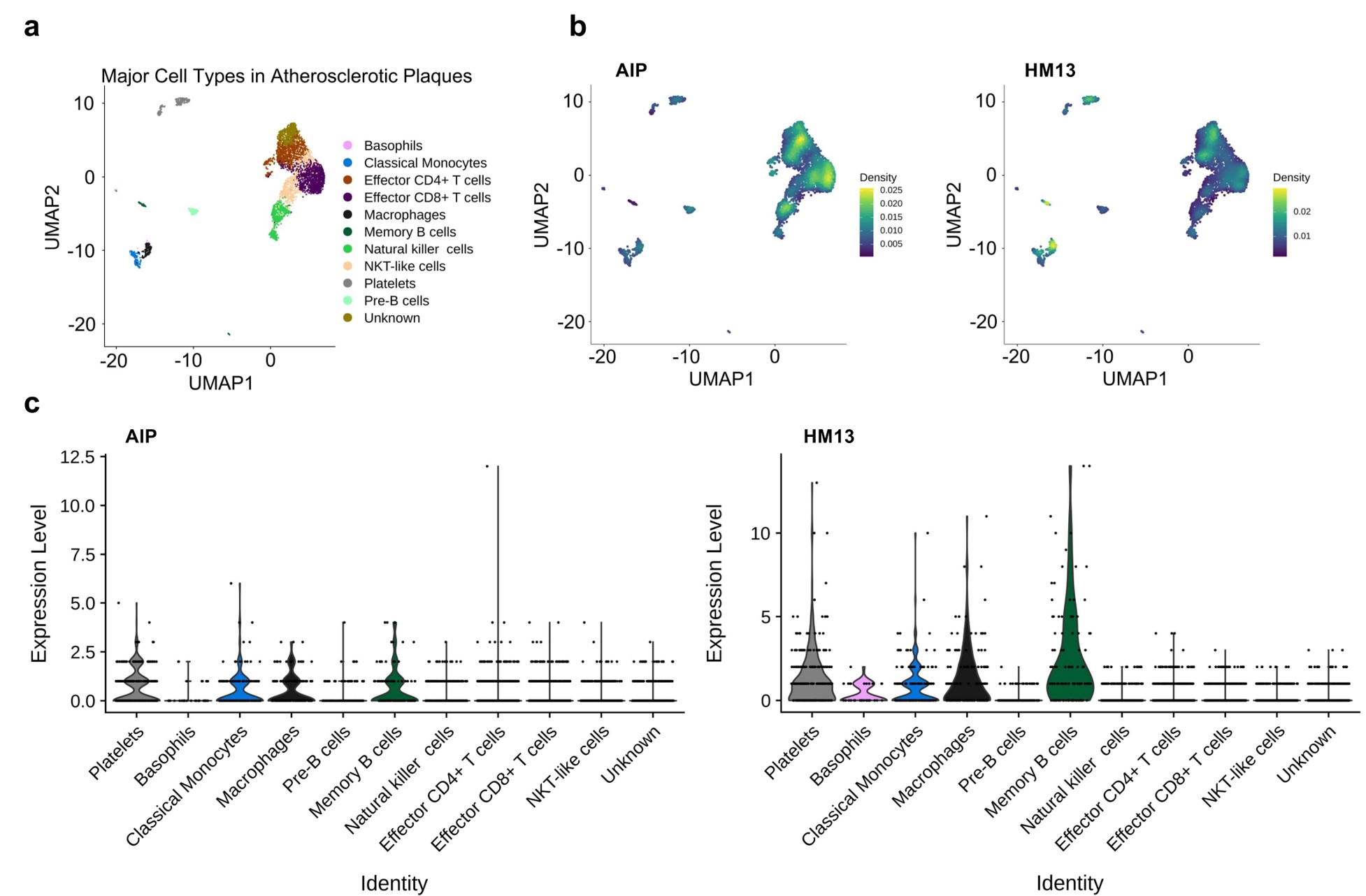


## Supporting Figure 10


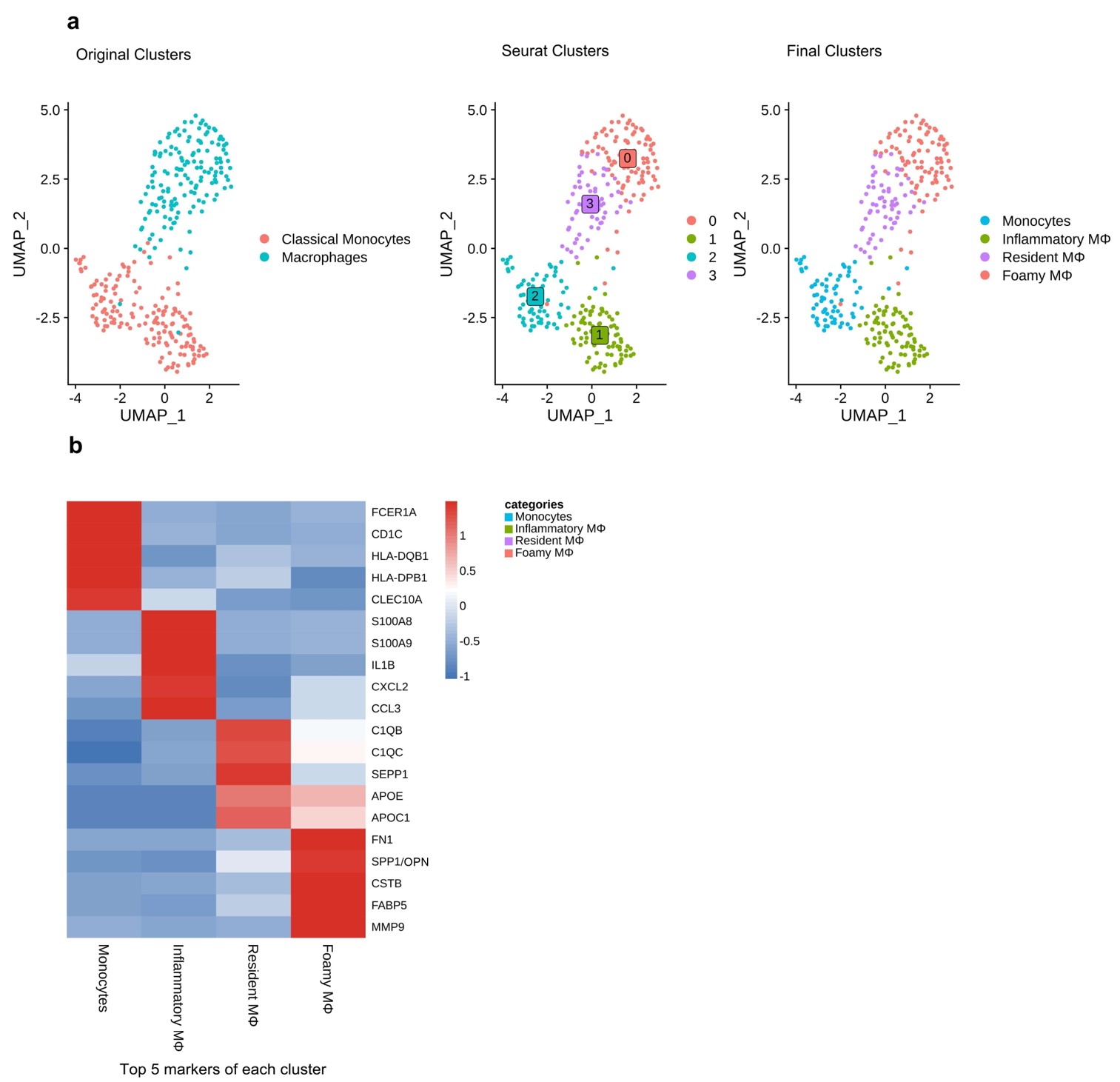


## Supporting Figure 11


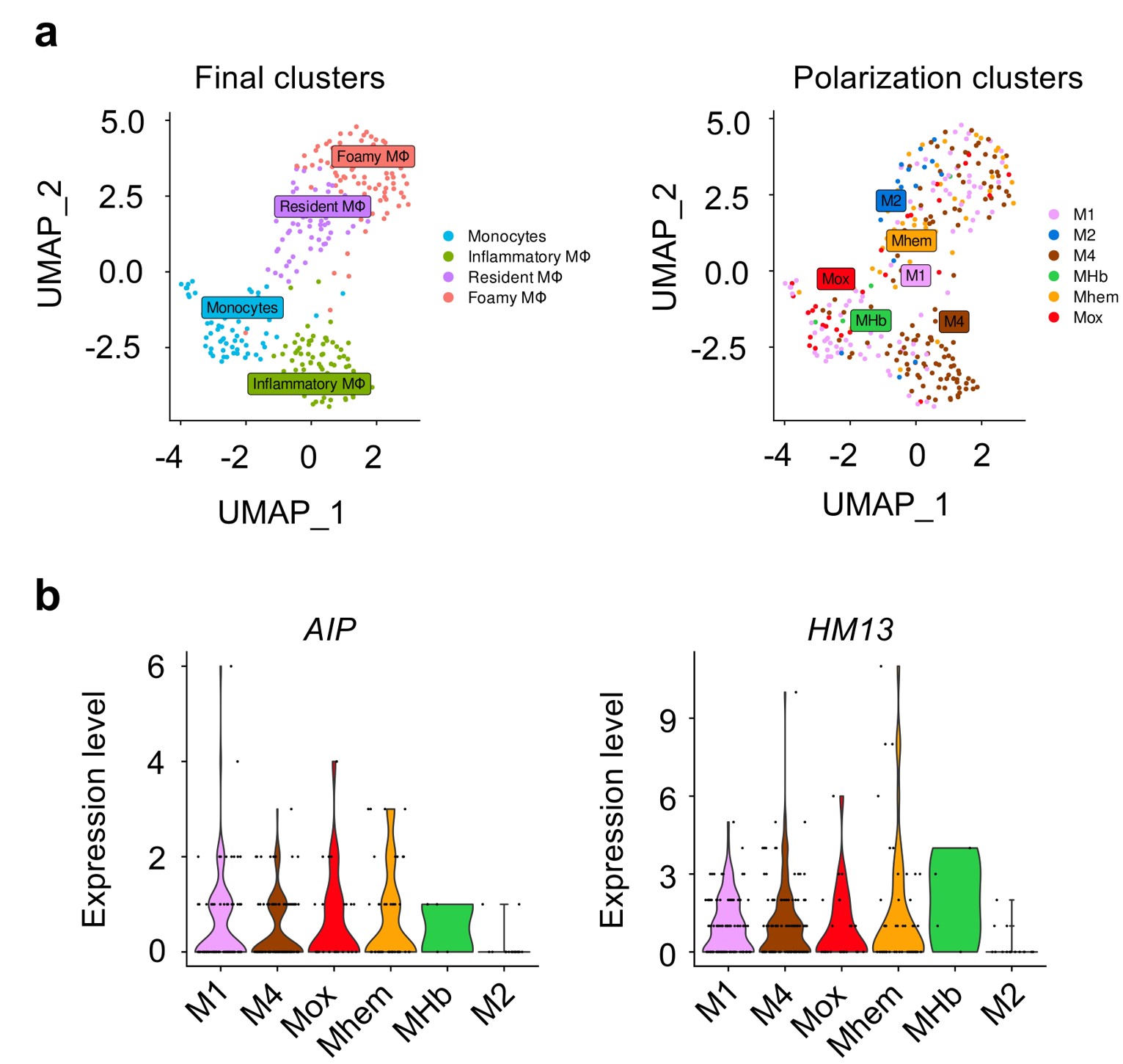


## Supporting Figure 12


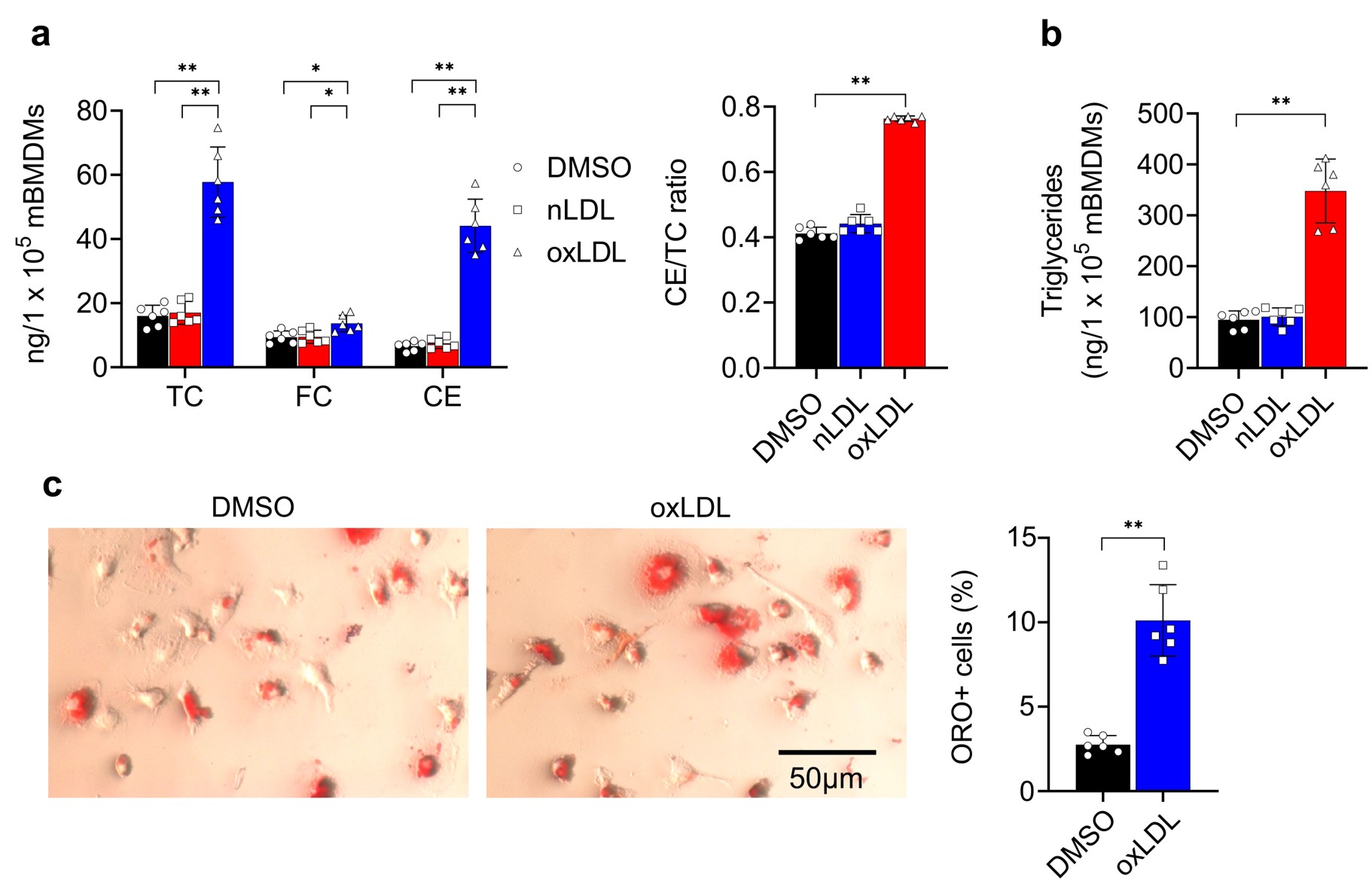


## Supporting Figure 13


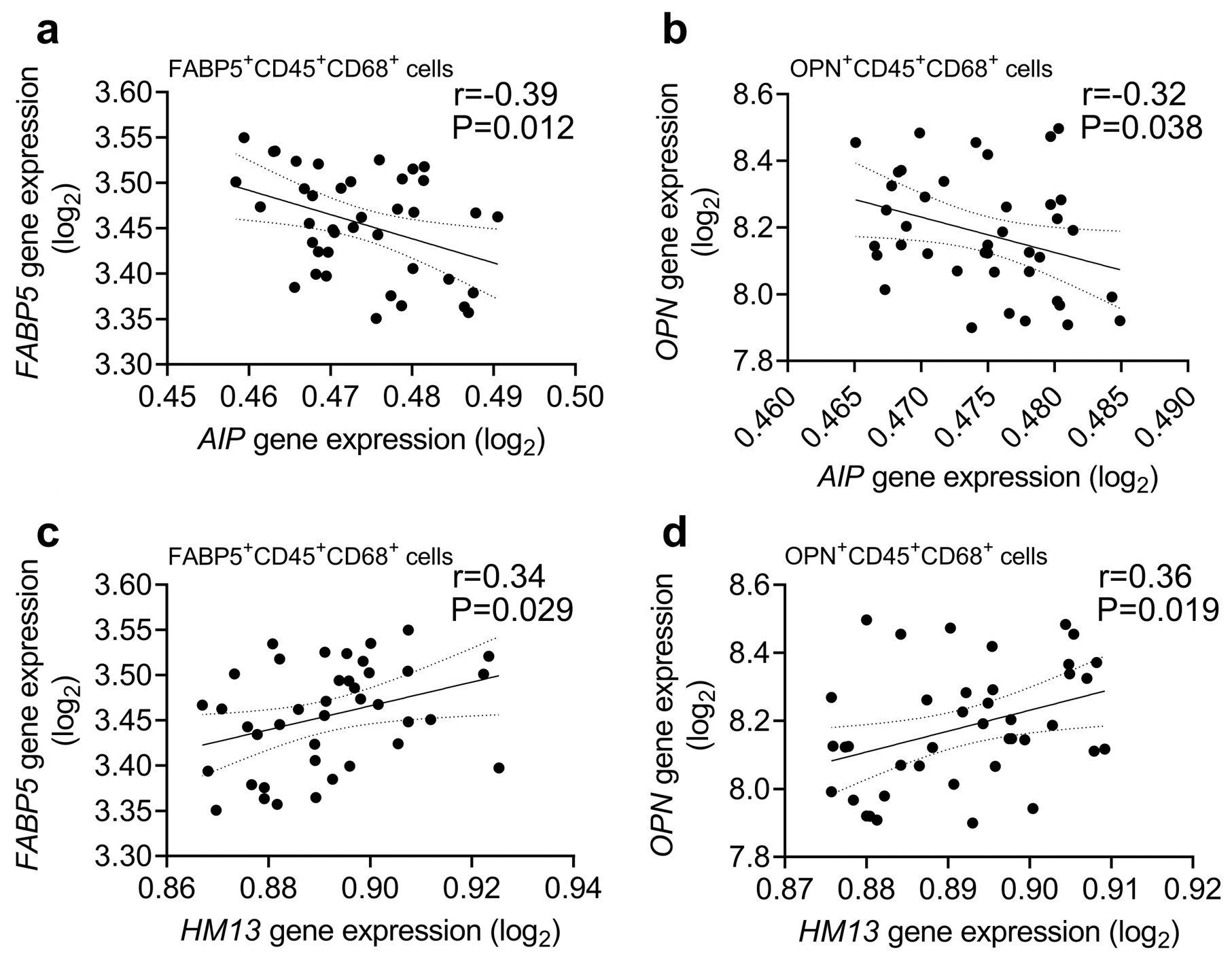


## Supporting Figure 14


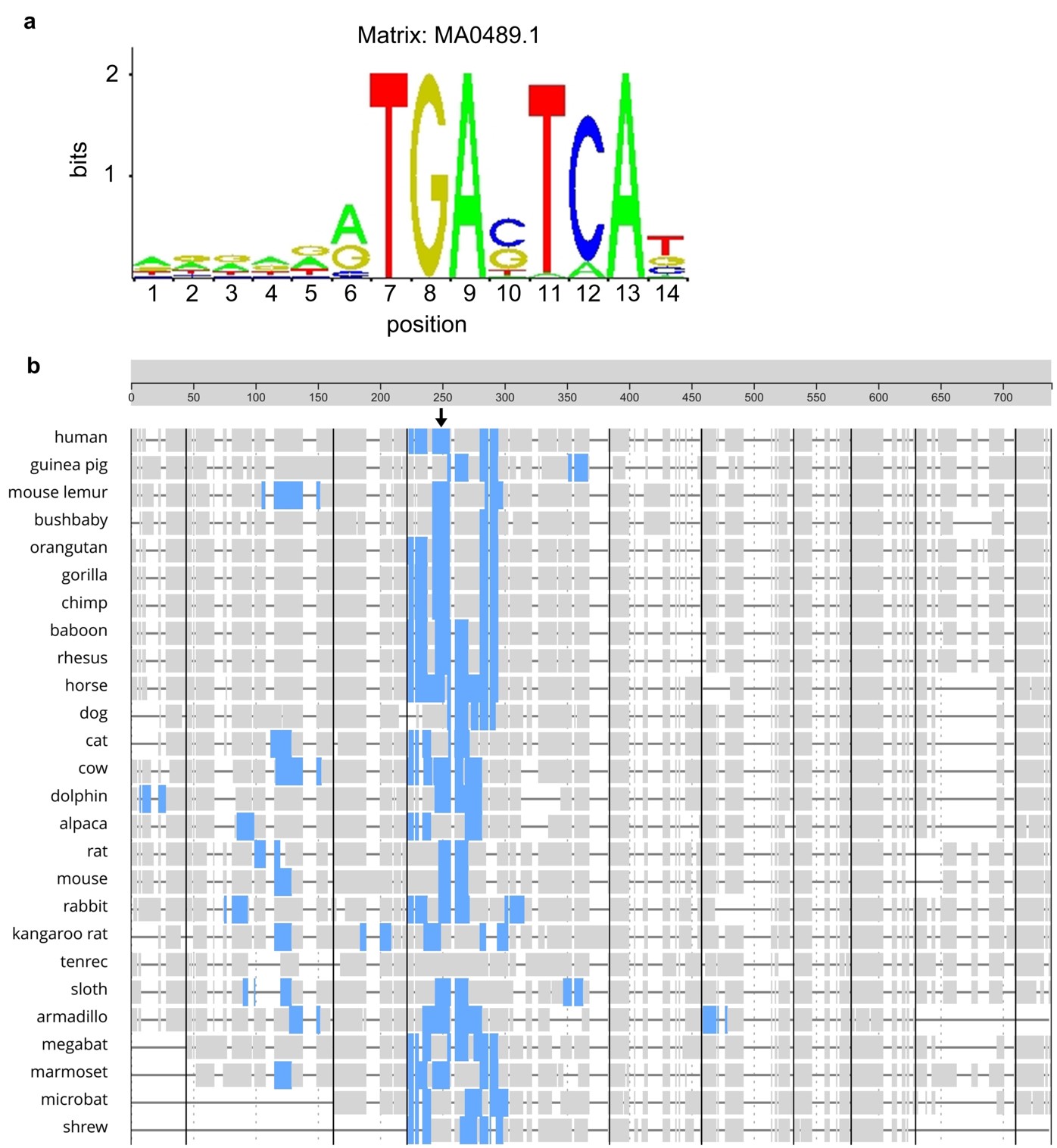


## Supporting Figure 15


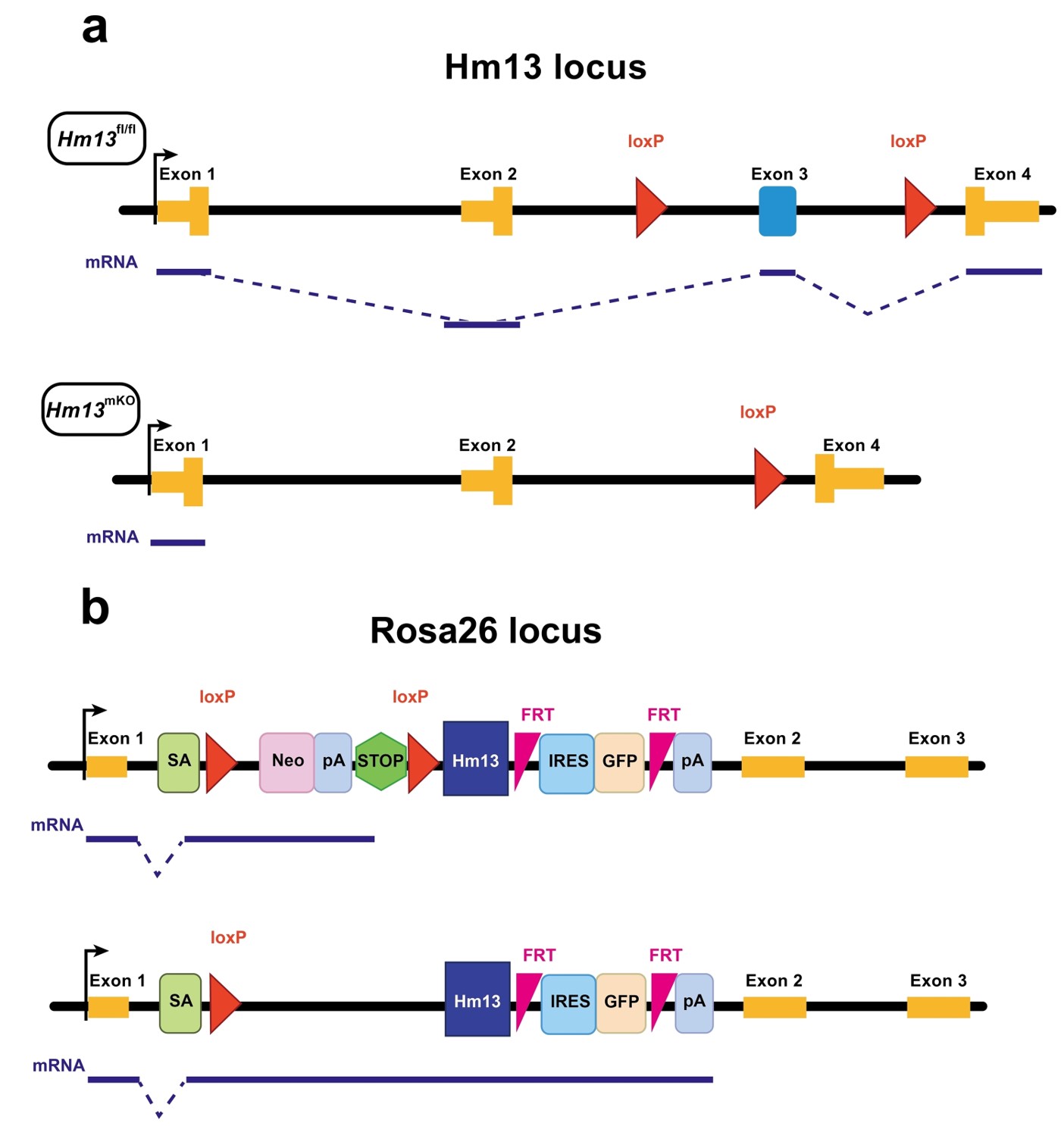


## Supporting Figure 16


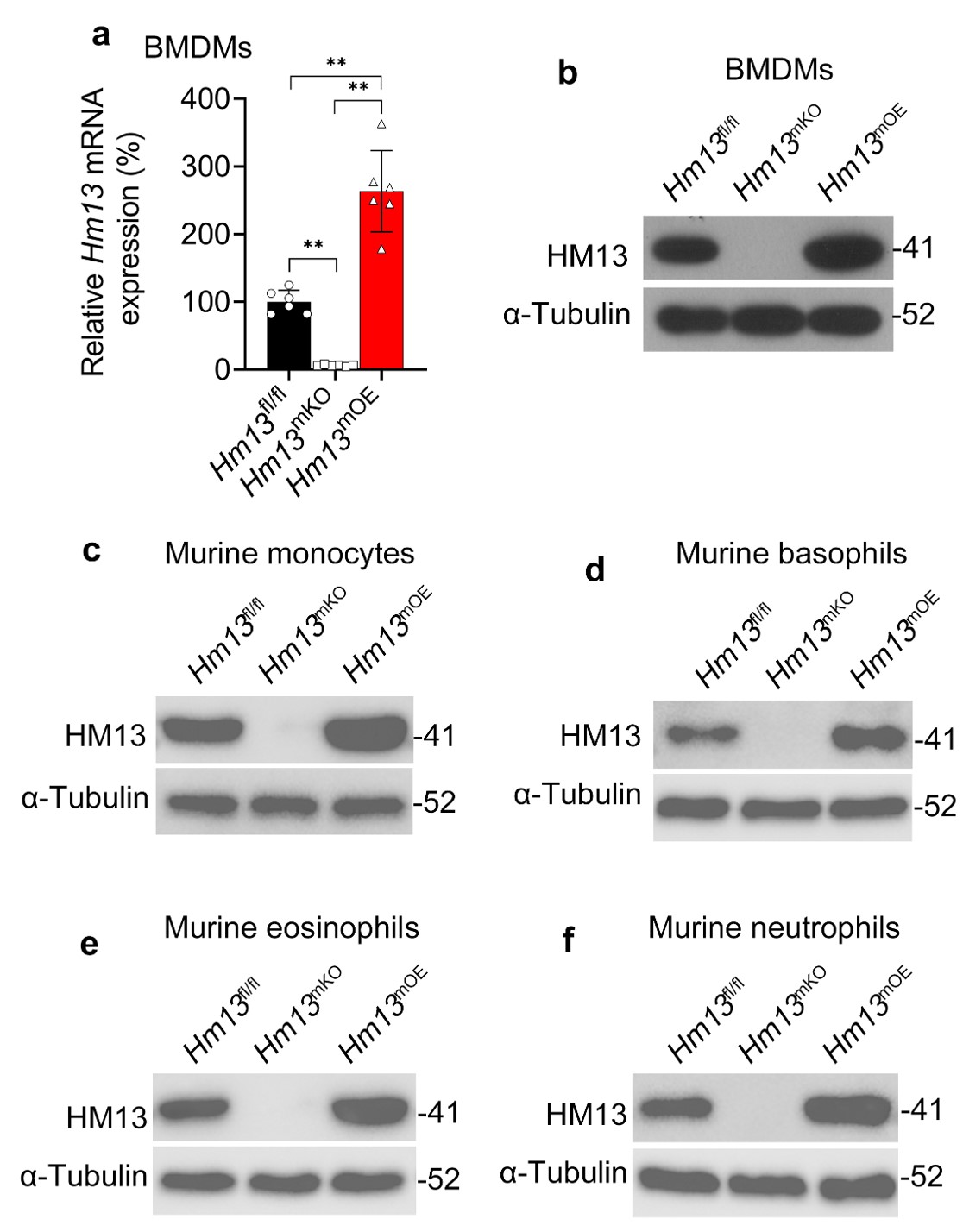


## Supporting Figure 17


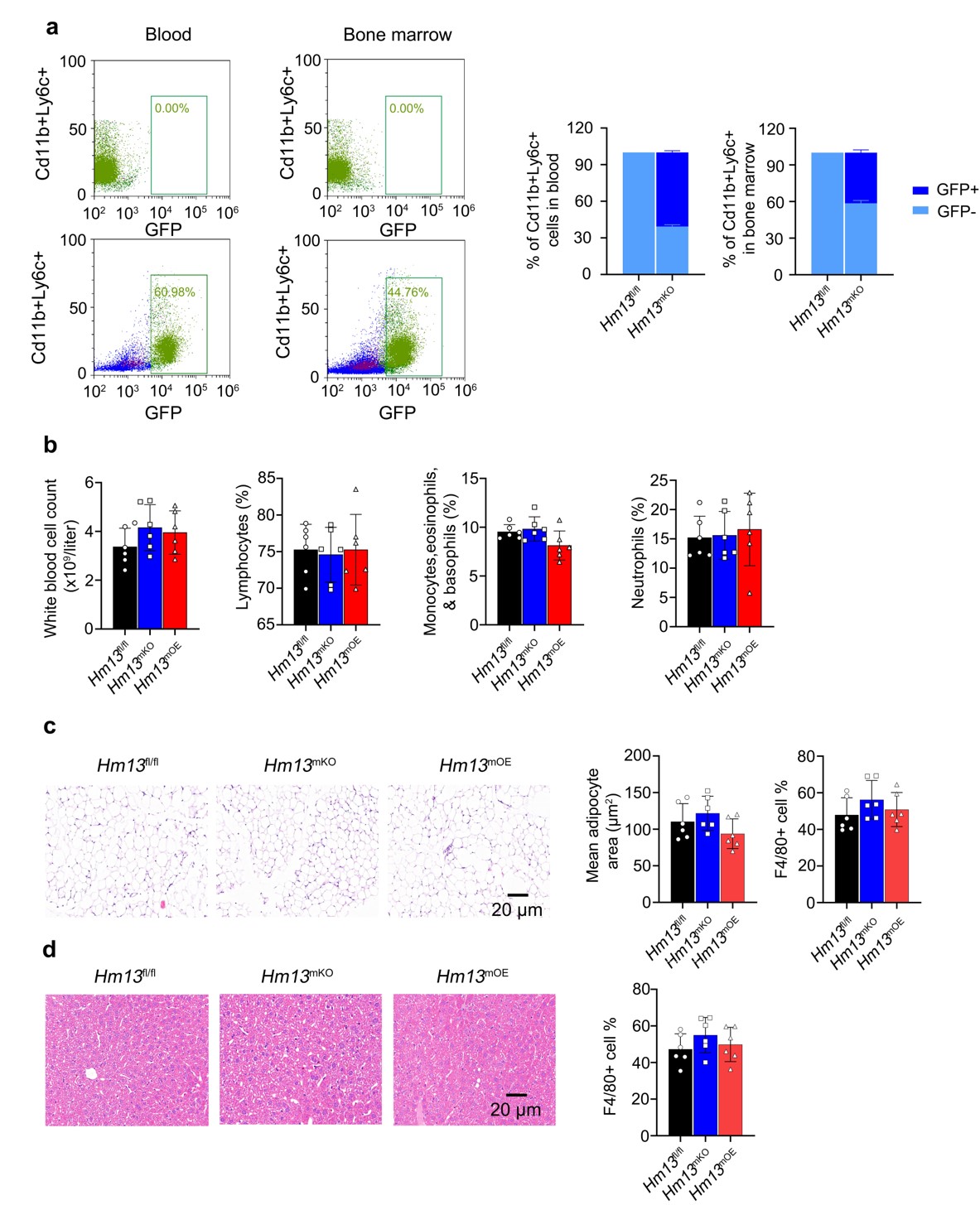


## Supporting Figure 18


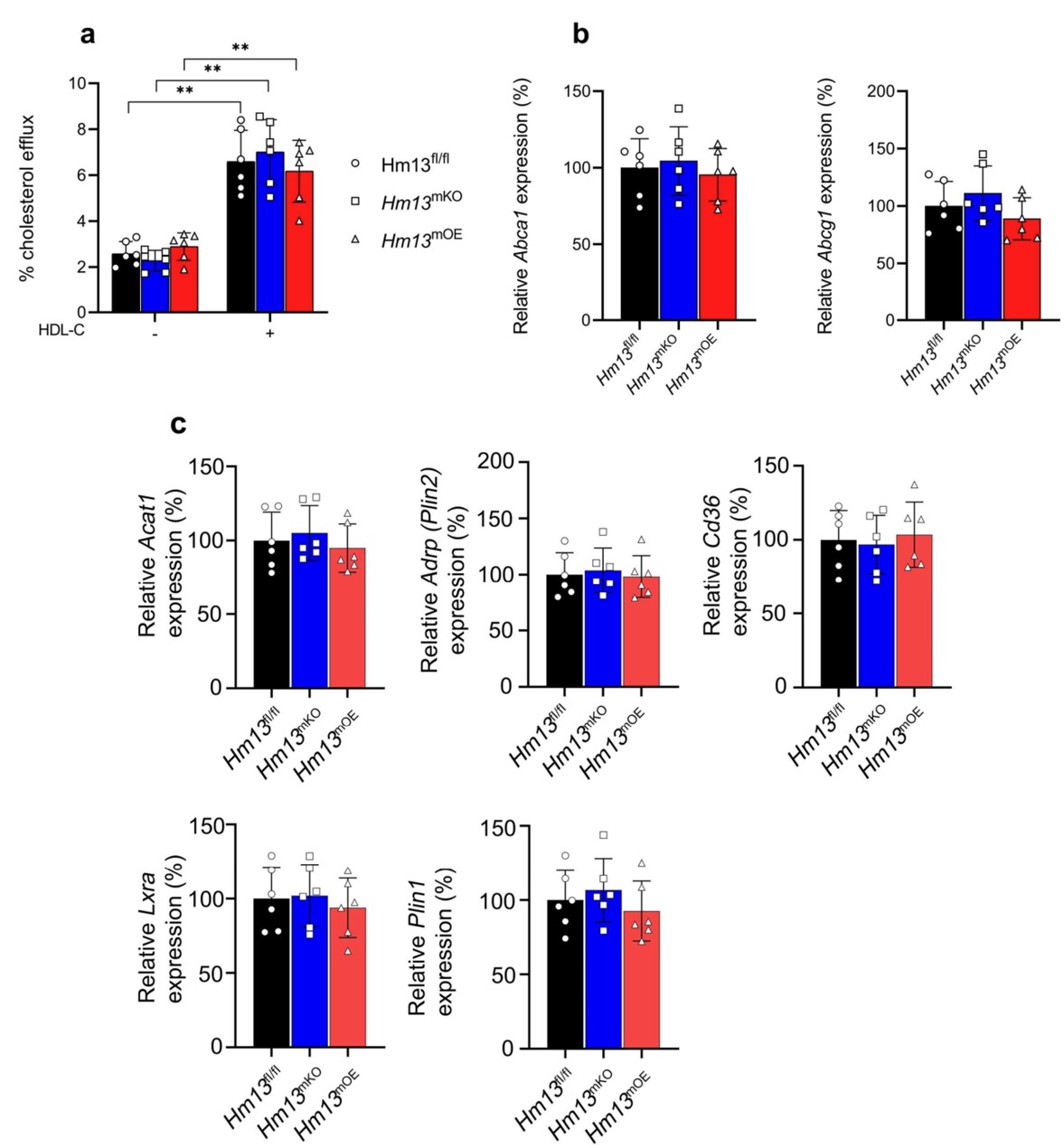


## Supporting Figure 19


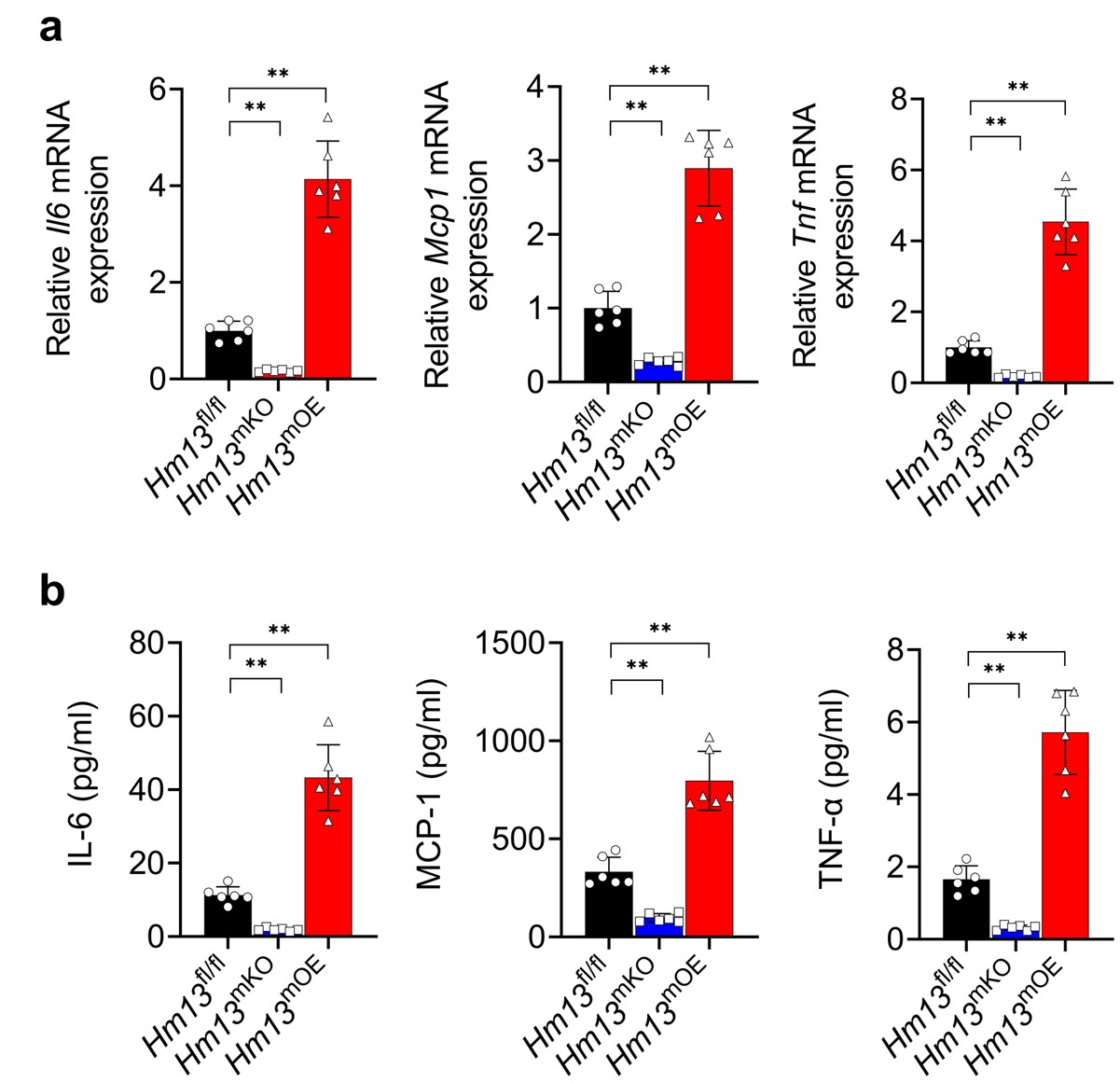


## Supporting Figure 20


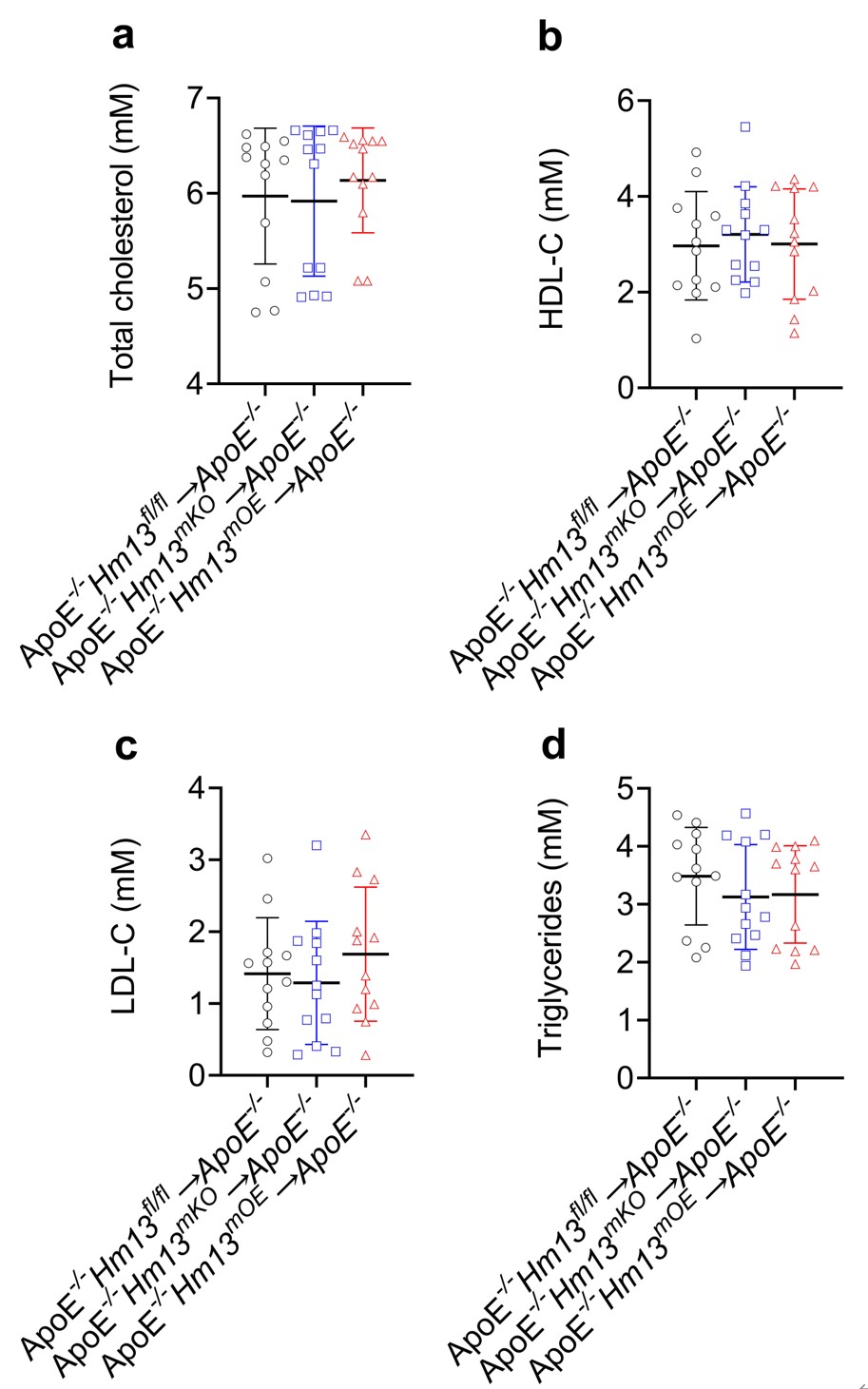


## Supporting Figure 21


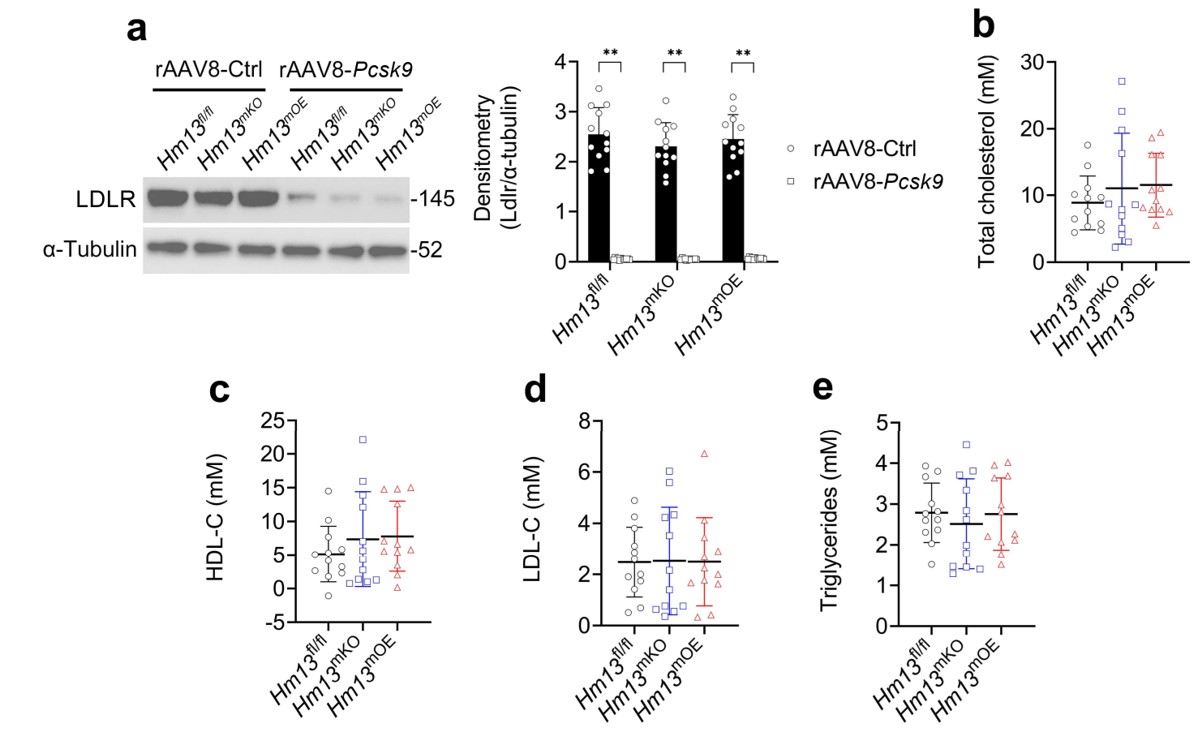


## Supporting Figure 22


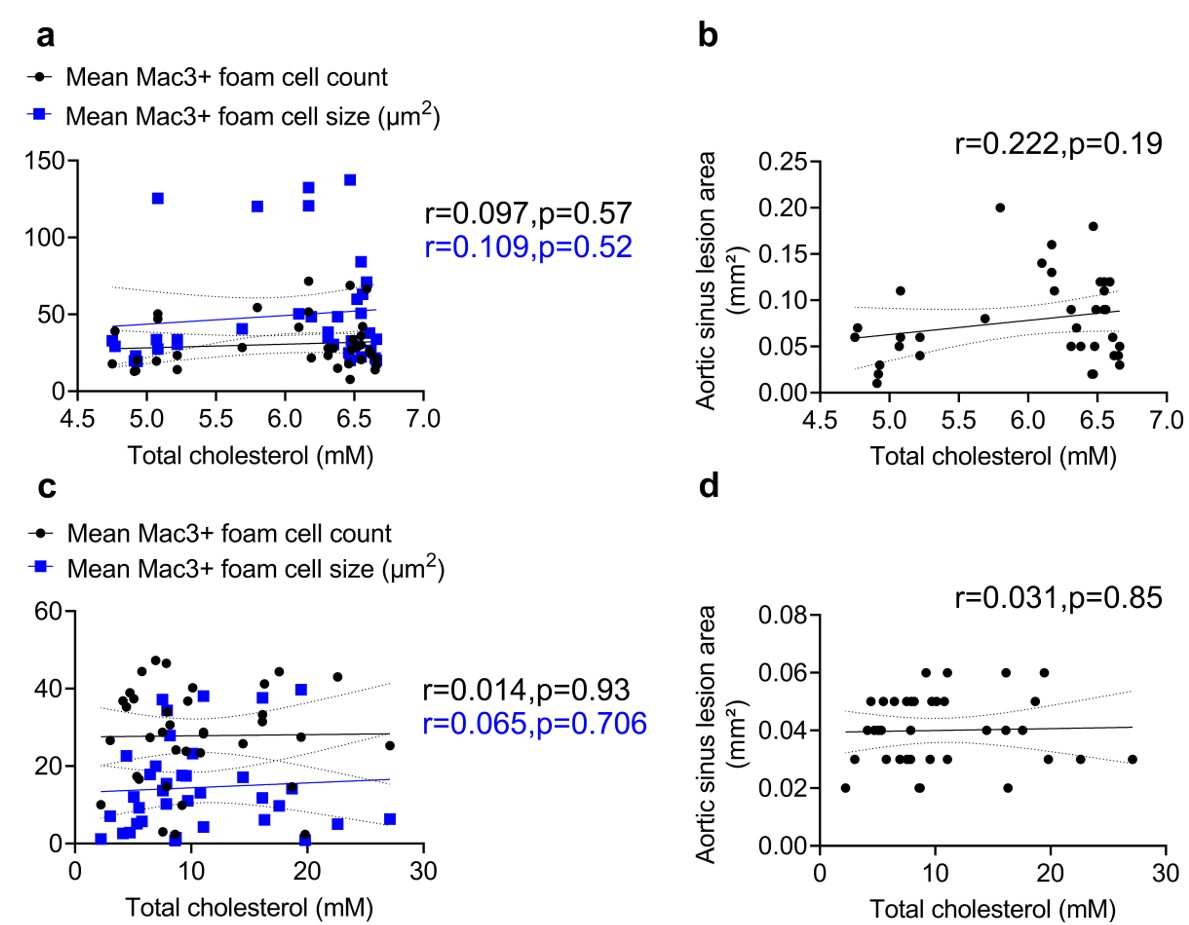


## Supporting Figure 23


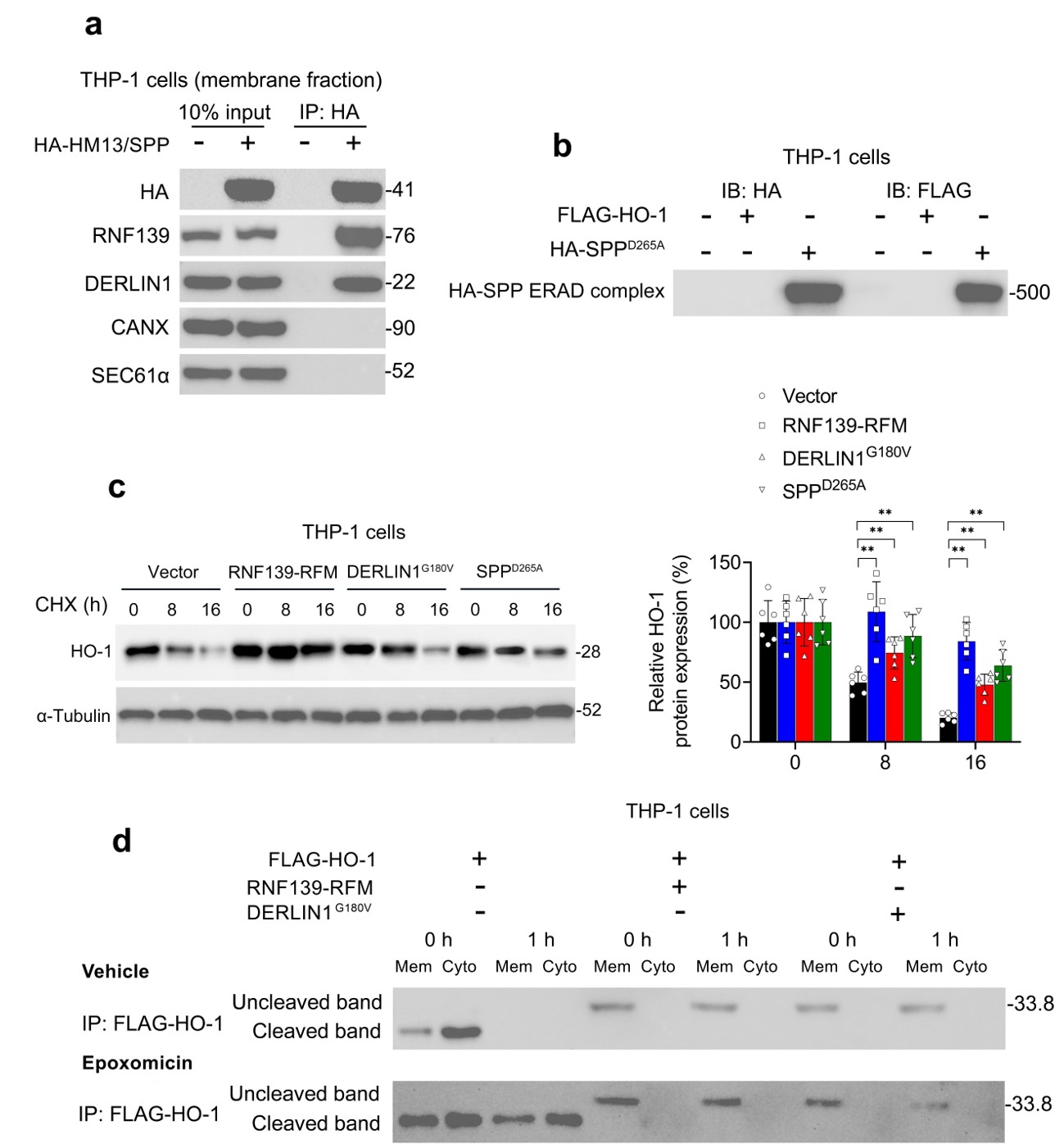


## Supporting Figure 24


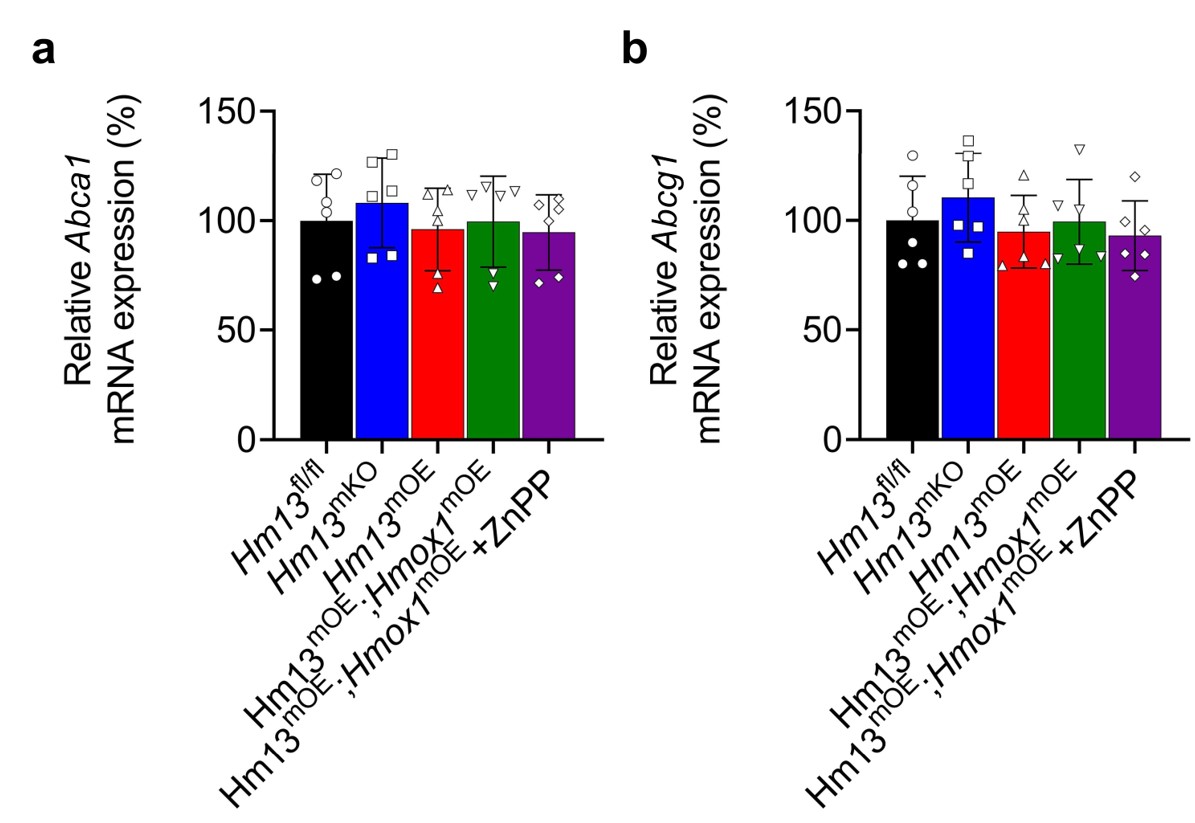


## Supporting Figure 25


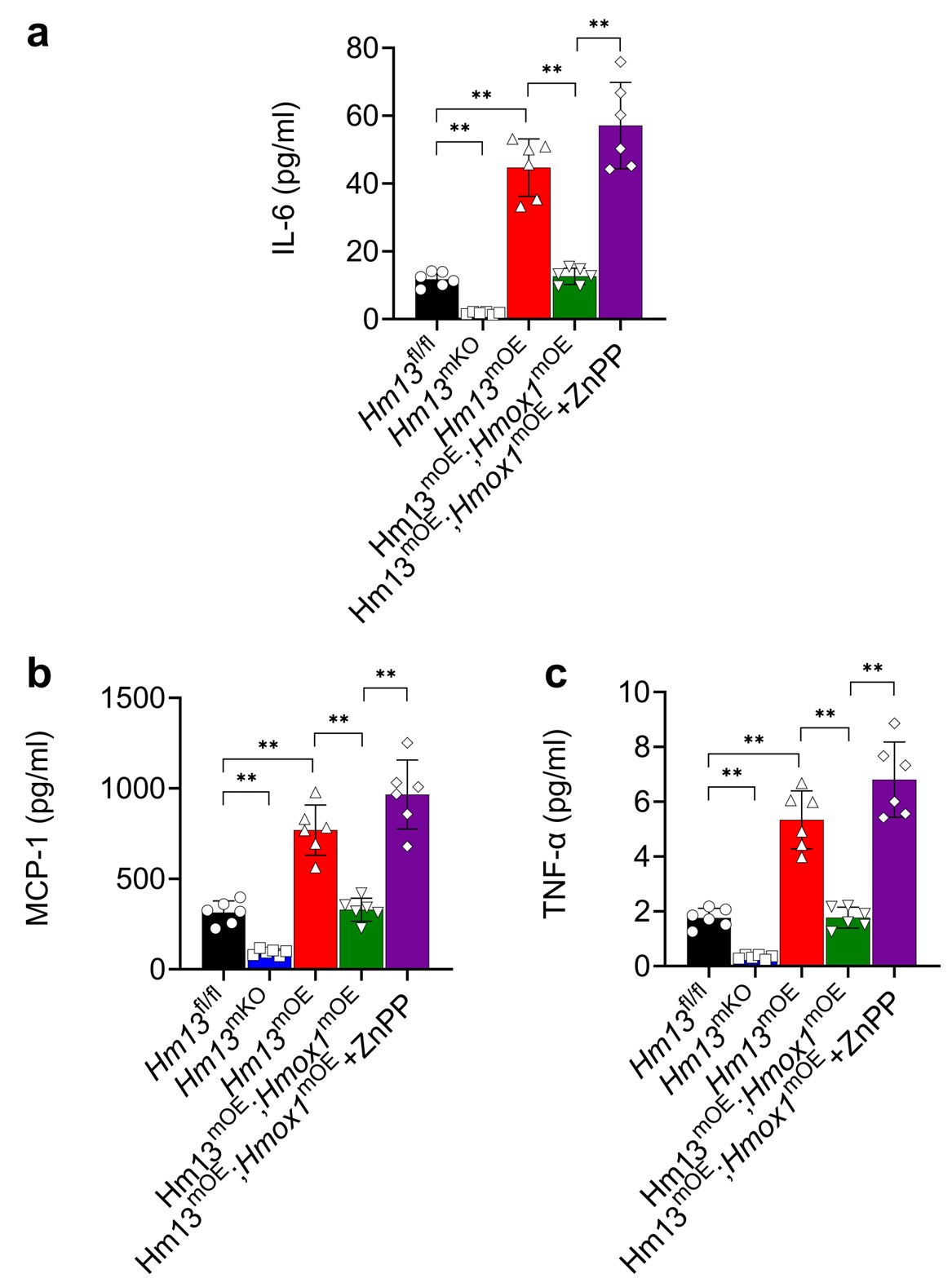


## Supporting Figure 26


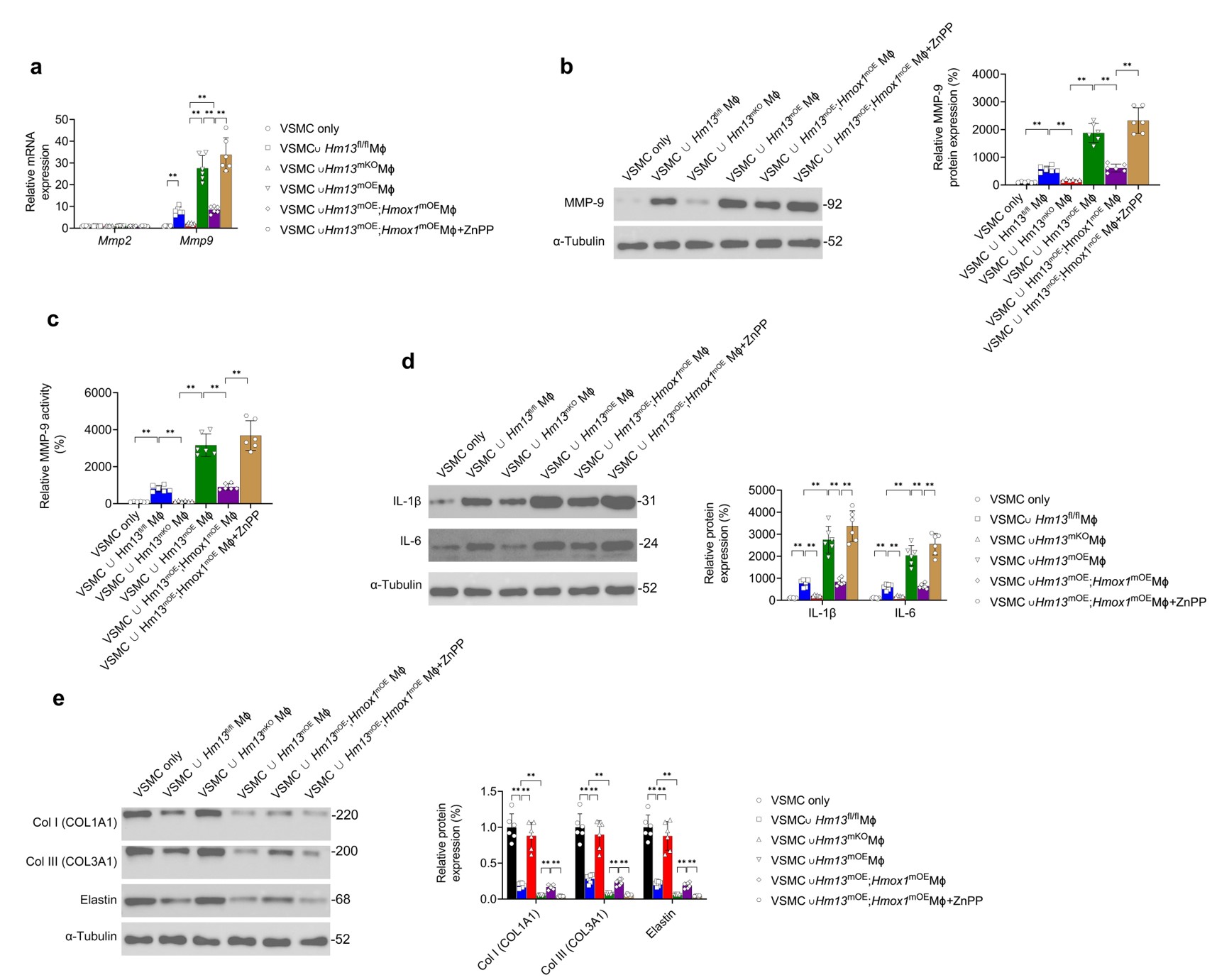


# Supporting References

[1] a) L. Kolberg, N. Kerimov, H. Peterson, K. Alasoo, *Elife* **2020**, *9*, e58705; b) Q. Liao, C. Liu, X. Yuan, S. Kang, R. Miao, H. Xiao, G. Zhao, H. Luo, D. Bu, H. Zhao, *Nucleic acids research* **2011**, *39* (9), 3864; c) T. Wang, J. Zhang, K. Huang, *BMC bioinformatics* **2019**, *20* (7), 17.

[2] M. B. Eisen, P. T. Spellman, P. O. Brown, D. Botstein, *Proceedings of the National Academy of Sciences* **1998**, *95* (25), 14863.

[3] A.-L. Barabási, N. Gulbahce, J. Loscalzo, *Nature reviews genetics* **2011**, *12* (1), 56.

[4] J. M. Stuart, E. Segal, D. Koller, S. K. Kim, *science* **2003**, *302* (5643), 249.

[5] O. Troyanskaya, M. Cantor, G. Sherlock, P. Brown, T. Hastie, R. Tibshirani, D. Botstein, R. B. Altman, *Bioinformatics* **2001**, *17* (6), 520.

[6] D. M. Ribeiro, C. Ziyani, O. Delaneau, *Communications Biology* **2022**, *5* (1), 876.

[7] J. T. Gaublomme, N. Yosef, Y. Lee, R. S. Gertner, L. V. Yang, C. Wu, P. P. Pandolfi, T. Mak, R. Satija, A. K. Shalek, *Cell* **2015**, *163* (6), 1400.

[8] S. Hägg, J. Skogsberg, J. Lundström, P. Noori, R. Nilsson, H. Zhong, S. Maleki, M.-M. Shang, B. Brinne, M. Bradshaw, *PLoS genetics* **2009**, *5* (12), e1000754.

[9] a) J. Fan, T. Watanabe, *Pathology international* **2022**, *72* (3), 151; b) B. E. Veseli, P. Perrotta, G. R. De Meyer, L. Roth, C. Van der Donckt, W. Martinet, G. R. De Meyer, *European journal of pharmacology* **2017**, *816*, 3.

[10] S. Kraler, P. Libby, P. C. Evans, A. Akhmedov, M. O. Schmiady, M. Reinehr, G. G. Camici, T. F. Lüscher, *Arteriosclerosis, thrombosis, and vascular biology* **2021**, *41* (8), 2237.

[11] P. Langfelder, S. Horvath, *BMC bioinformatics* **2008**, *9* (1), 1.

[12] J. Hardin, A. Mitani, L. Hicks, B. VanKoten, *BMC bioinformatics* **2007**, *8*, 1.

[13] M. J. Gandal, J. R. Haney, N. N. Parikshak, V. Leppa, G. Ramaswami, C. Hartl, A. J. Schork, V. Appadurai, A. Buil, T. M. Werge, *Science* **2018**, *359* (6376), 693.

[14] B. Zhang, C. Gaiteri, L.-G. Bodea, Z. Wang, J. McElwee, A. A. Podtelezhnikov, C. Zhang, T. Xie, L. Tran, R. Dobrin, *Cell* **2013**, *153* (3), 707.

[15] A. T. McKenzie, I. Katsyv, W.-M. Song, M. Wang, B. Zhang, *BMC systems biology* **2016**, *10*, 1.

[16] H. A. Talukdar, H. F. Asl, R. K. Jain, R. Ermel, A. Ruusalepp, O. Franzén, B. A. Kidd, B. Readhead, C. Giannarelli, J. C. Kovacic, *Cell systems* **2016**, *2* (3), 196.

[17] S. C. Ritchie, S. Watts, L. G. Fearnley, K. E. Holt, G. Abraham, M. Inouye, *Cell systems* **2016**, *3* (1), 71.

[18] S. Cagnin, M. Biscuola, C. Patuzzo, E. Trabetti, A. Pasquali, P. Laveder, G. Faggian, M. Iafrancesco, A. Mazzucco, P. F. Pignatti, *BMC genomics* **2009**, *10*, 1.

[19] O. Puig, J. Yuan, S. Stepaniants, R. Zieba, E. Zycband, M. Morris, S. Coulter, X. Yu, J. Menke, J. Woods, *Circulation: Cardiovascular Genetics* **2011**, *4* (6), 595.

[20] D. M. Fernandez, A. H. Rahman, N. F. Fernandez, A. Chudnovskiy, E.-a. D. Amir, L. Amadori, N. S. Khan, C. K. Wong, R. Shamailova, C. A. Hill, *Nature medicine* **2019**, *25* (10), 1576.

[21] K. Mulder, A. A. Patel, W. T. Kong, C. Piot, E. Halitzki, G. Dunsmore, S. Khalilnezhad, S. E. Irac, A. Dubuisson, M. Chevrier, *Immunity* **2021**, *54* (8), 1883.

[22] A. Ianevski, A. K. Giri, T. Aittokallio, *Nature communications* **2022**, *13* (1), 1246.

[23] L. Willemsen, M. P. de Winther, *The Journal of pathology* **2020**, *250* (5), 705.

[24] I. Gianopoulos, S. S. Daskalopoulou, *Basic Research in Cardiology* **2024**, *119* (1), 35.

[25] a) R. Chen, H. Zhang, B. Tang, Y. Luo, Y. Yang, X. Zhong, S. Chen, X. Xu, S. Huang, C. Liu, *Signal Transduction and Targeted Therapy* **2024**, *9* (1), 130; b) P. Theofilis, E. Oikonomou, K. Tsioufis, D. Tousoulis, *International Journal of Molecular Sciences* **2023**, *24* (11), 9568; c) M. Fu, S. Jia, L. Xu, X. Li, Y. Lv, Y. Zhong, S. Ai, *The Journal of Clinical Investigation* **2024**, *134* (19).

[26] Ł. Kreft, A. Soete, P. Hulpiau, A. Botzki, Y. Saeys, P. De Bleser, *Nucleic acids research* **2017**, *45* (W1), W490.

[27] J. Xiong, Z. Li, H. Tang, Y. Duan, X. Ban, K. Xu, Y. Guo, Y. Tu, *BMC biology* **2023**, *21* (1), 46.

[28] a) M. Piollet, F. Porsch, G. Rizzo, F. Kapser, D. J. Schulz, M. G. Kiss, K. Schlepckow, E. Morenas-Rodriguez, M. O. Sen, J. Gropper, *Nature cardiovascular research* **2024**, *3* (3), 269; b) M. T. Patterson, Y. Xu, H. Hillman, V. Osinski, P. R. Schrank, A. E. Kennedy, F. Barrow, A. Zhu, S. Tollison, S. Shekhar, *Arteriosclerosis, Thrombosis, and Vascular Biology* **2024**.

[29] a) V. R. Babaev, R. P. Runner, D. Fan, L. Ding, Y. Zhang, H. Tao, E. Erbay, C. Z. Görgün, S. Fazio, G. S. Hotamisligil, *Arteriosclerosis, thrombosis, and vascular biology* **2011**, *31* (6), 1283; b) M. Furuhashi, M. Ogura, M. Matsumoto, S. Yuda, A. Muranaka, M. Kawamukai, A. Omori, M. Tanaka, N. Moniwa, H. Ohnishi, *Scientific reports* **2017**, *7* (1), 217.

[30] H. Ito, T. Wakatsuki, K. Yamaguchi, D. Fukuda, Y. Kawabata, T. Matsuura, K. Kusunose, T. Ise, T. Tobiume, S. Yagi, *Circulation Journal* **2020**, *84* (5), 769.

[31] K. Poels, J. G. Schnitzler, F. Waissi, J. H. Levels, E. S. Stroes, M. J. Daemen, E. Lutgens, A.-M. Pennekamp, D. P. De Kleijn, T. T. Seijkens, *Frontiers in Cell and Developmental Biology* **2020**, *8*, 581641.

[32] M. E. Rosenfeld, J. C. Khoo, E. Miller, S. Parthasarathy, W. Palinski, J. L. Witztum, *The Journal of clinical investigation* **1991**, *87* (1), 90.

[33] Y. Sasaki, E. Derudder, E. Hobeika, R. Pelanda, M. Reth, K. Rajewsky, M. Schmidt-Supprian, *Immunity* **2006**, *24* (6), 729.

[34] A. F. Daly, J.-F. Vanbellinghen, S. K. Khoo, M.-L. Jaffrain-Rea, L. A. Naves, M. A. Guitelman, A. Murat, P. Emy, A.-P. Gimenez-Roqueplo, G. Tamburrano, *The Journal of Clinical Endocrinology & Metabolism* **2007**, *92* (5), 1891.

[35] I. Darlyuk-Saadon, C. Bai, C. K. M. Heng, N. Gilad, W.-P. Yu, P. Y. Lim, A. Cazenave-Gassiot, Y. Zhang, W. F. Wong, D. Engelberg, *Proceedings of the National Academy of Sciences* **2021**, *118* (14), e2018069118.

[36] C. Dunn, C. Wiltshire, A. MacLaren, D. A. Gillespie, *Cellular signalling* **2002**, *14* (7), 585.

[37] Y. Nei, K. Obata-Ninomiya, H. Tsutsui, K. Ishiwata, M. Miyasaka, K. Matsumoto, S. Nakae, H. Kanuka, N. Inase, H. Karasuyama, *Proceedings of the National Academy of Sciences* **2013**, *110* (46), 18620.

[38] S. Rose, A. Misharin, H. Perlman, *Cytometry Part A* **2012**, *81* (4), 343.

[39] C. y. Chen, N. S. Malchus, B. Hehn, W. Stelzer, D. Avci, D. Langosch, M. K. Lemberg, *The EMBO journal* **2014**, *33* (21), 2492.

[40] M. L. Burr, F. Cano, S. Svobodova, L. H. Boyle, J. M. Boname, P. J. Lehner, *Proceedings of the National Academy of Sciences* **2011**, *108* (5), 2034.

[41] N. Adhikari, K. C. Shekar, R. Staggs, Z. Win, K. Steucke, Y.-W. Lin, L.-N. Wei, P. Alford, J. L. Hall, *Journal of cardiovascular translational research* **2015**, *8*, 158.

[42] E. Butoi, A. Gan, M. Tucureanu, D. Stan, R. Macarie, C. Constantinescu, M. Calin, M. Simionescu, I. Manduteanu, *Biochimica et Biophysica Acta (BBA)-Molecular Cell Research* **2016**, *1863* (7), 1568.
